# Supplementary material for: NME3 is a gatekeeper for DRP1-dependent mitophagy in hypoxia
Source: Nat Commun. 2024 Mar 13;15:2264. doi: 10.1038/s41467-024-46385-7 (PMC10938004; doi:10.1038/s41467-024-46385-7)
Supplement: Supplementary file 1 — Supplementary Information [file 41467_2024_46385_MOESM1_ESM.pdf]

Supplementary Information

NME3 is a gatekeeper for DRP1-dependent mitophagy in hypoxia

Chih-Wei Chen, Chi Su, Chang-Yu Huang, Xuan-Rong Huang, Xiaojing Cuili, Tung Chao, Chun-Hsiang Fan, Cheng-Wei Ting, Yi-Wei Tsai, Kai-Chien Yang, Ti-Yen Yeh, Sung-Tsang Hsieh, Yi-Ju Chen, Yuxi Feng, Tony Hunter, and Zee-Fen Chang*

*Correspondence to: Zee-Fen Chang (zfchang@ntu.edu.tw)

This includes:
Supplementary Figure S1 to S7
Supplementary Table 1

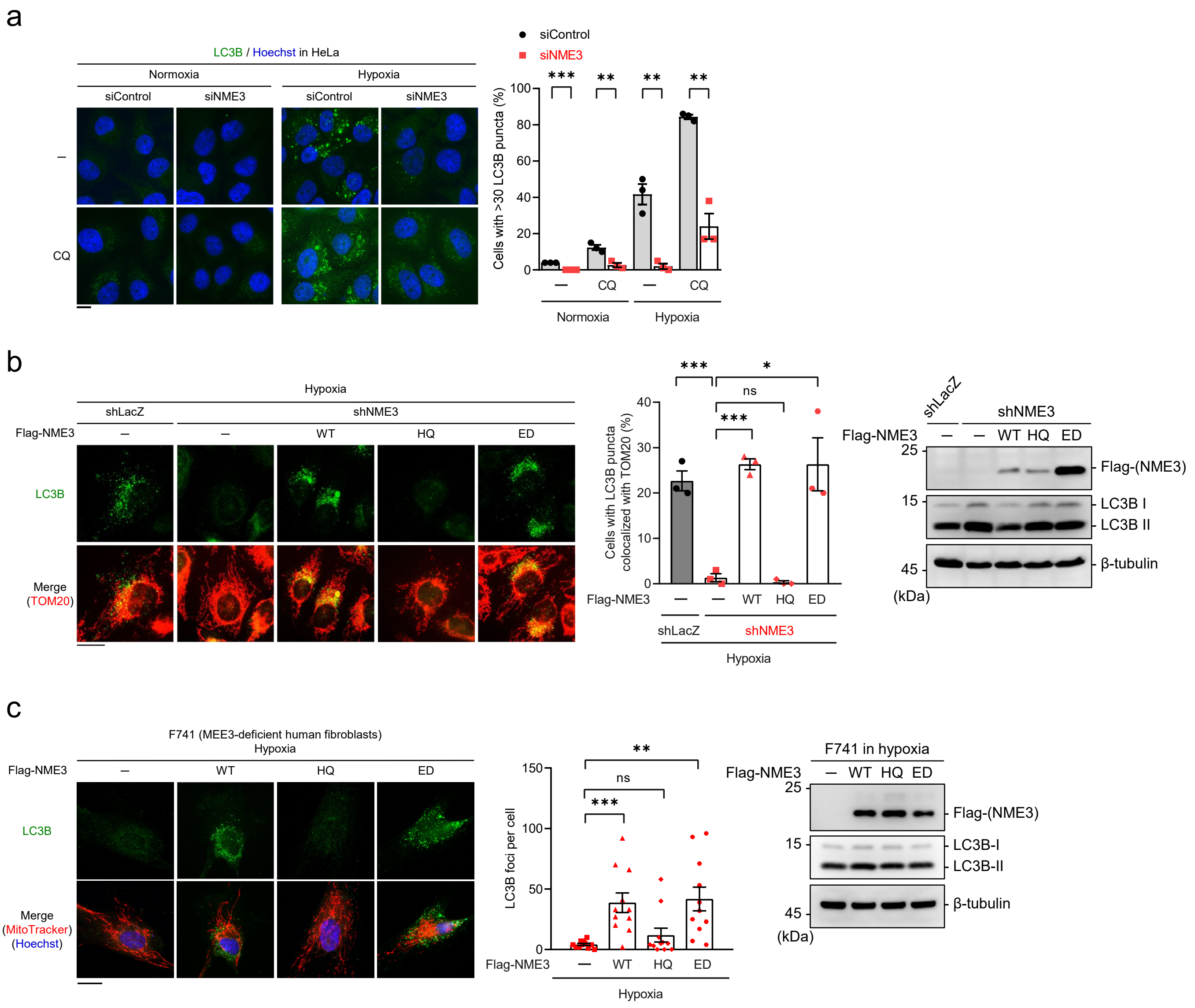

Supplementary Figure S1. NME3 knockdown impairs hypoxia-induced puncta independent of lysosomal flux.
(a) HeLa cells transfected with control and NME3 siRNA were incubated in normoxia and hypoxia for 20 h followed by treatment with and without chloroquine for 4 h prior to fixation and IF stained with antibody of LC3B. (*Left*) Representative images are shown and (*Right*) the percentage of cells with prominent LC3B puncta are shown as mean ± SEM (n = 3 independent experiments). Scale bar, 20 µm. **(b)** Expression of H135 phosphorylable NME3 rescues Hypoxia-induced mitophagy in NME3 depleted cells. Effect of NME3 variants on restoring hypoxia-induced mitophagy. HeLa cells infected with lentivirus of shRNA of LacZ and NME3 were transfected with expression vector of wild-type (WT), H135Q (HQ) and E40/46D (ED) mutant of Flag-NME3 resistant to shRNA of NME3. After hypoxia for 24 h, cells were fixed for immunofluorescence (IF) staining of LC3 and TOM20, scale bar, 20 µm. Percentage of cells with prominent LC3B puncta colocalized with TOM20 were shown as mean ± SEM (n = 3 independent experiments). Western blot analysis using antibodies against Flag, LC3B, and β-tubulin. **(c)** F741 fibroblasts derived from a patient carrying homozygous mutation at the initiation codon of *NME3* were transfected by modified mRNA of wild-type (WT), H135Q (HQ), and E40/46D (ED) mutants of NME3. These cells were incubated in hypoxia chamber (0.5% oxygen) for 24 h followed by fixation of LC3B IF staining together with MitoTracker Red for mitochondrial and Hoechst for DNA staining. Scale bar, 20 µm. (*Left*) Representative images are shown and (*Middle*) the percentage of cells containing prominent LC3B puncta associated with MitoTracker and Hoechst are shown (11 cells were analyzed). (*Right*) shows Western blot of the expression of NME3 variants. NS means no significant, *$p < 0.05$, **$p < 0.01$, ***$p < 0.001$; two-tailed t test.

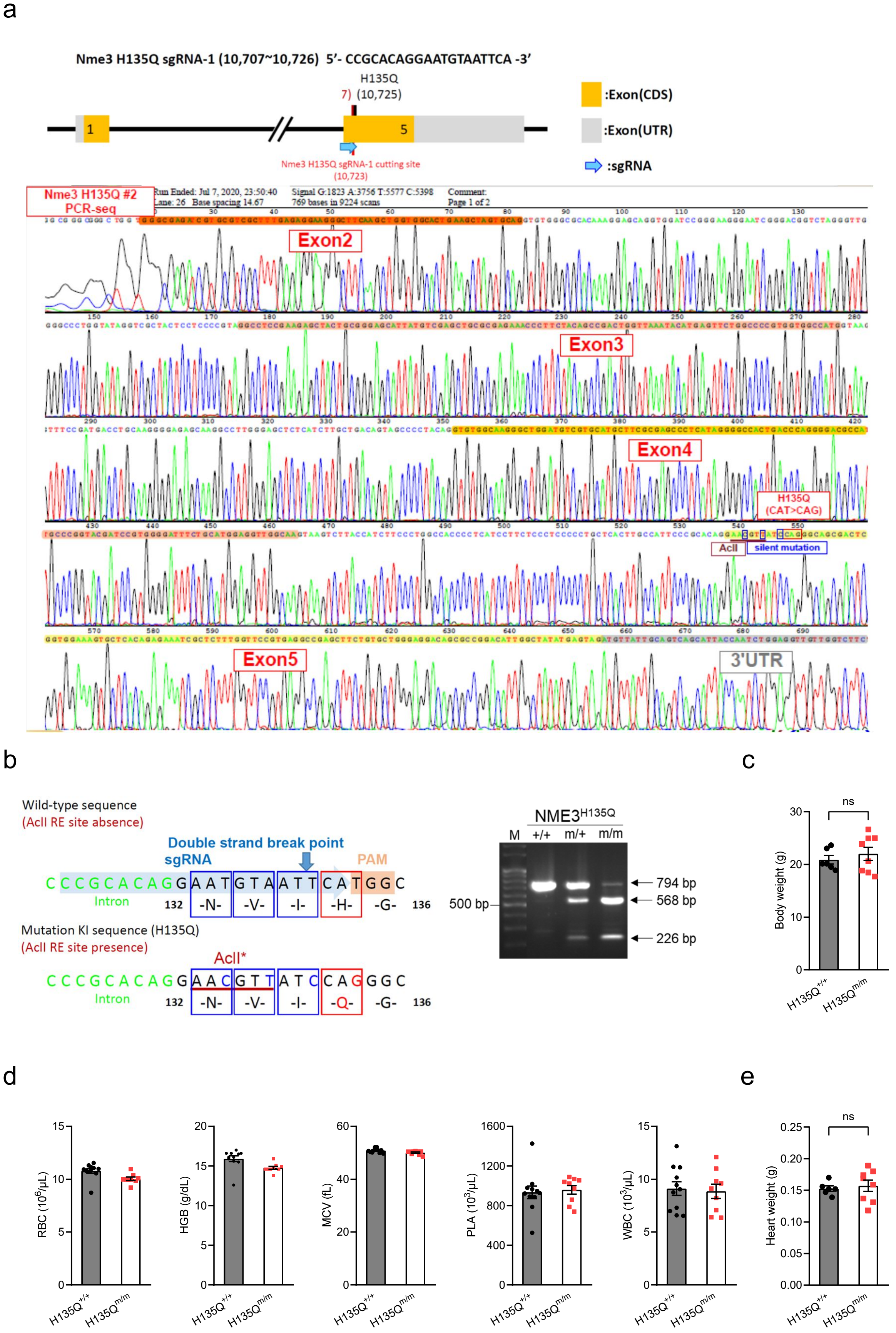
a
Nme3 H135Q sgRNA-1 (10,707~10,726) 5'- CCGCACAGGAATGTAATTCA -3'
H135Q
(10,725)
1
5
Nme3 H135Q sgRNA-1 cutting site
(10,723)
:Exon(CDS)
:Exon(UTR)
:sgRNA
Nme3 H135Q #2
PCR-seq
Exon2
Exon3
Exon4
H135Q
(CAT>CAG)
AclI
silent mutation
Exon5
3'UTR
b
Wild-type sequence
(AclI RE site absence)
Double strand break point
sgRNA
PAM
C C C G C A C A G G A A T G T A A T T C A T G G C
Intron
132 -N- -V- -I- -H- -G- 136
Mutation KI sequence (H135Q)
(AclI RE site presence)
AclI*
C C C G C A C A G G A A C G T T A T C C A G G G C
Intron
132 -N- -V- -I- -Q- -G- 136
NME3H135Q
M +/+ m/+ m/m
500 bp
794 bp
568 bp
226 bp
c
ns
Body weight (g)
H135Q+/+
H135Qm/m
d
RBC (10^6/μL)
HGB (g/dL)
MCV (fL)
PLA (10^3/μL)
WBC (10^3/μL)
H135Q+/+
H135Qm/m
e
ns
Heart weight (g)
H135Q+/+
H135Qm/m

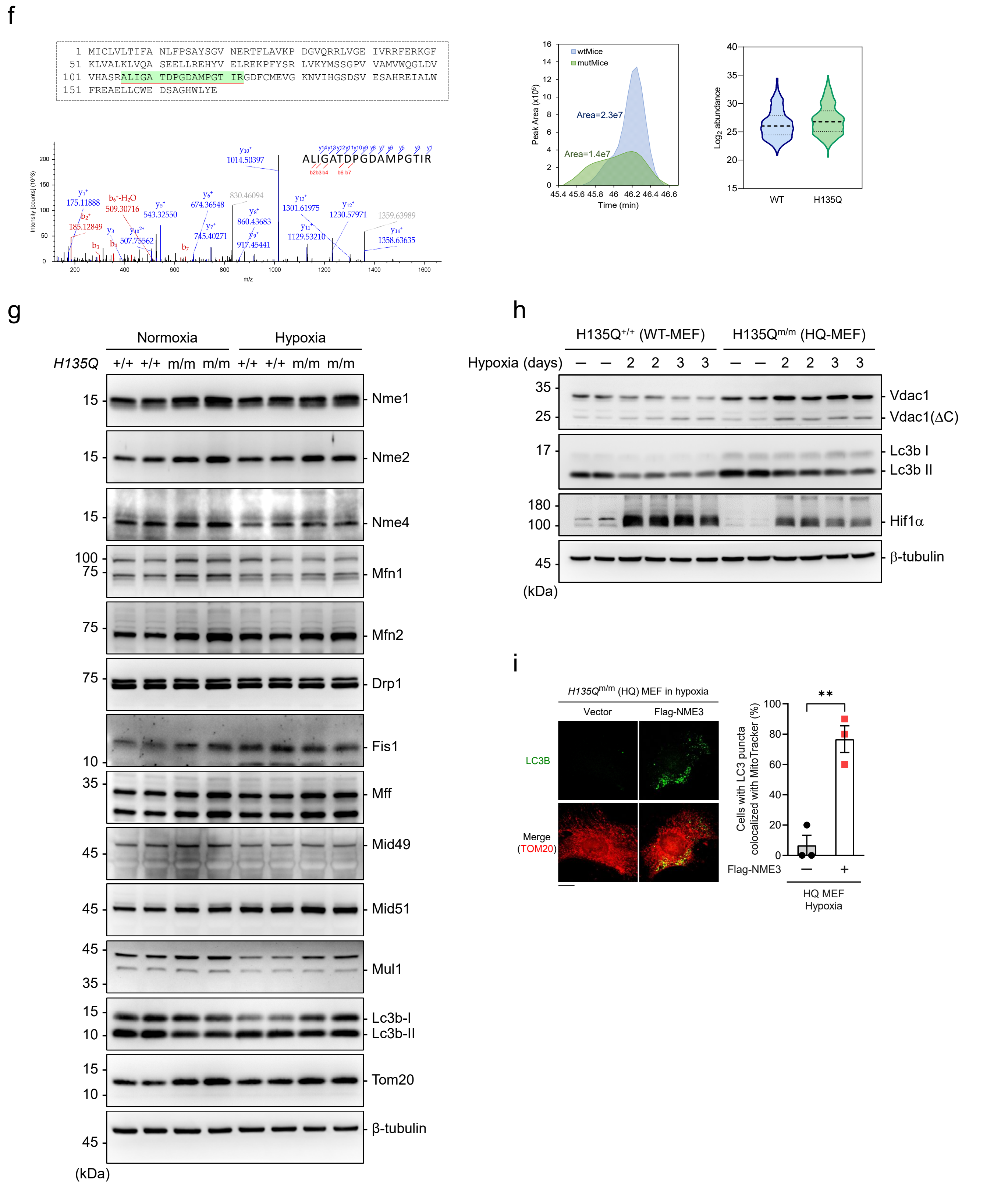

Supplementary Figure S2. The generation of mice harboring H135Q mutation at *Nme3* and characterization.
(a) The design of *Nme3 H135Q* knock-in mutation in mice using CRISPR-Cas9 and the mouse sequencing result. **(b)** Genotyping results of *Nme3 H135Q* mutant mice: *Nme3* wild-type (WT) (+/+), *Nme3 H135Q* heterozygous knock-in (m/+), and homozygous knock-in (m/m). **(c)** The body weights and **(e)** the heart weight of sacrificed mice after I/R experiment. n = 6 for $H135Q^{+/+}$ I/R mice; and n = 8 for $H135Q^{m/m}$ I/R mice. NS means no significant; two-tailed *t* test. **(d)** Blood test in 11 WT and 9 *H135Q* mice at ages between 14-to-18-week-old. Red blood cell (RBC) counts, hemoglobin (HGB), mean corpuscular volume (MCV), platelet (PLA), and white blood (WBC). **(f)** Mass spectrometer for Nme3 protein in mice cerebellar of *Nme3* $H135Q^{+/+}$ and $H135Q^{m/m}$ mice. *Left* shows the unique mouse NME3 peptide (green) identified by LC/MS/MS. *Right* shows the extracted ion peaks of the peptide from WT and H135Q cerebellum with the peak area calculated and below shows the protein abundance of two samples. **(g, h)** MEFs from $H135Q^{+/+}$ and $H135Q^{m/m}$ mice were incubated in normoxia vs hypoxia for **(g)** 24 h and **(h)** 48-72 h. Two different sets of cells were harvested for Western blot analysis using antibodies as indicated. **(i)** $H135Q^{m/m}$ MEFs were transfected with vector and Flag-NME3. After incubation in hypoxia for 24 h, cells were fixed for IF staining of LC3B together with mitoTracker Red (*Left*), scale bar, 20 μm. (*Right*) the percentage of cells with prominent LC3B puncta colocalized with MitoTracker Red are shown as mean ± SEM (n = 3 independent experiments). NS means no significant, $^{**}p < 0.01$; two-tailed *t* test.

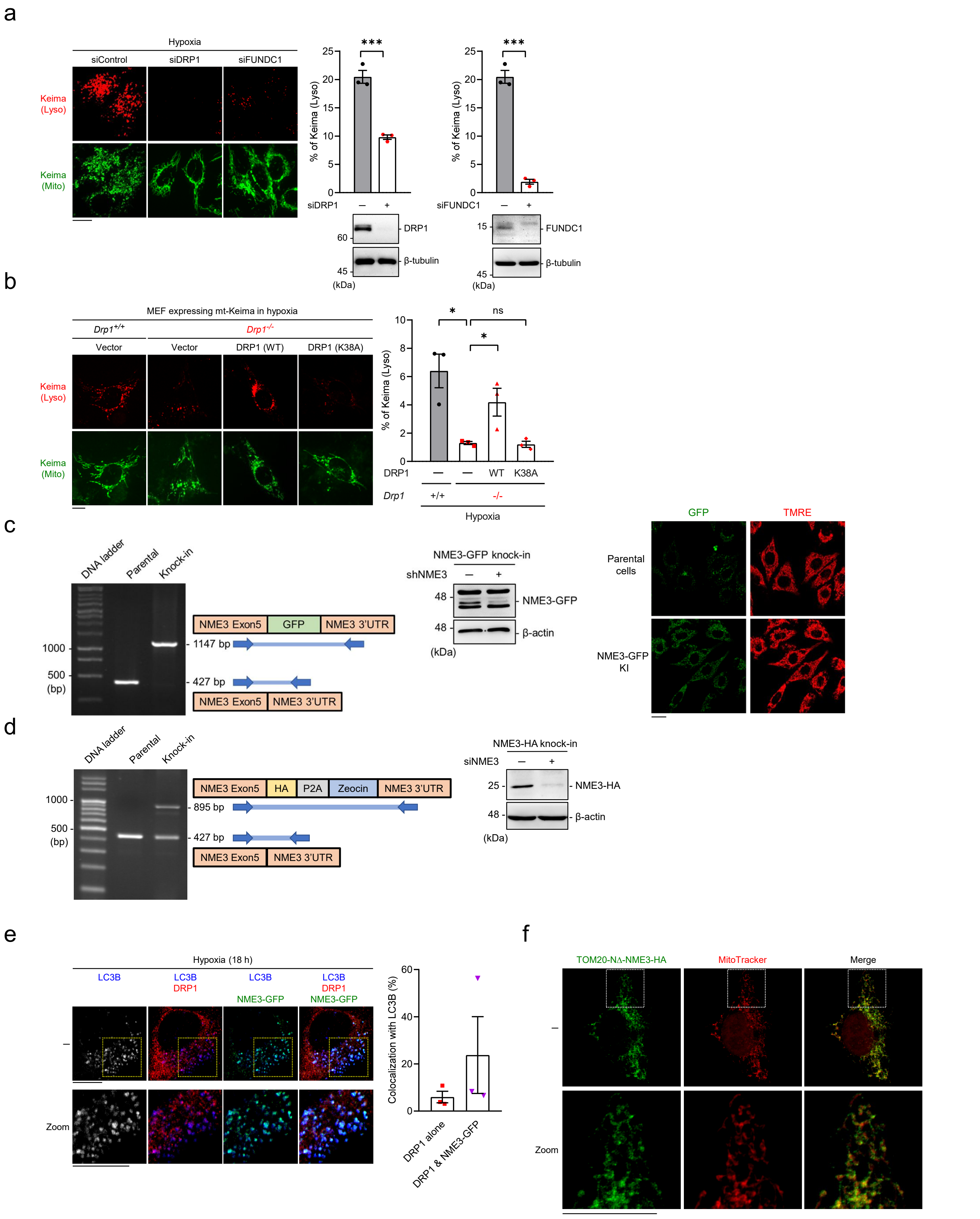

Supplementary Figure S3. DRP1 and NME3 cooperates in hypoxia-induced mitophagy.
(a) HeLa cells were transfected with control, DRP1, and FUNDC1 siRNA for 2 d and then incubated in hypoxia 24 h for mt-Keima analysis. Images, quantitation of the percentages of lysosomal Keima (≥75 cells were counted, n = 3) and Western blots are shown. Scale bar, 20 μm. **(b)** *Drp1*$^{+/+}$ and *Drp1*$^{-/-}$ MEFs stably expressing mt-Keima were transfected with WT or K38A catalytic dead mutant of DRP1 before incubated in hypoxia for 24 h. Images and quantitation of lysosomal Keima and total Keima fluorescence are shown (≥30 cells were counted, n = 3). Scale bar, 20 μm. *Right* shows the Western blot of the cells. NS means no significant, **p* < 0.05, ***p* < 0.01, ****p* < 0.001; two-tailed *t* test. **(c)** Schematic diagrams for the establishment of NME3-GFP knock-in clone with DNA analysis of NME3-GFP knock-in by PCR in HeLa, and the detection of endogenous NME3-GFP on mitochondria by fluorescence microcopy, scale bar, 20 μm. **(d)** The establishment of NME3-HA knock-in HeLa validated by PCR of genomic DNA and the detection of endogenous NME3-HA by Western blot using anti-HA antibody. **(e)** NME3-GFP cells incubated in hypoxia for 18 h were fixed for IF staining with antibody of LC3B and DRP1 for AiryScan super-resolution microscopy analysis, scale bar, 10 μm. Representative images are shown and the percentage of LC3B puncta colocalized DRP1 alone, or NME3-GFP-DRP1 together are shown as mean ± SEM (n = 3 cells). **(f)** Cells were transfected with TOM20-NΔ-NME3-HA vector and fixed for AiryScan super-resolution microscopy, scale bar, 10 μm.

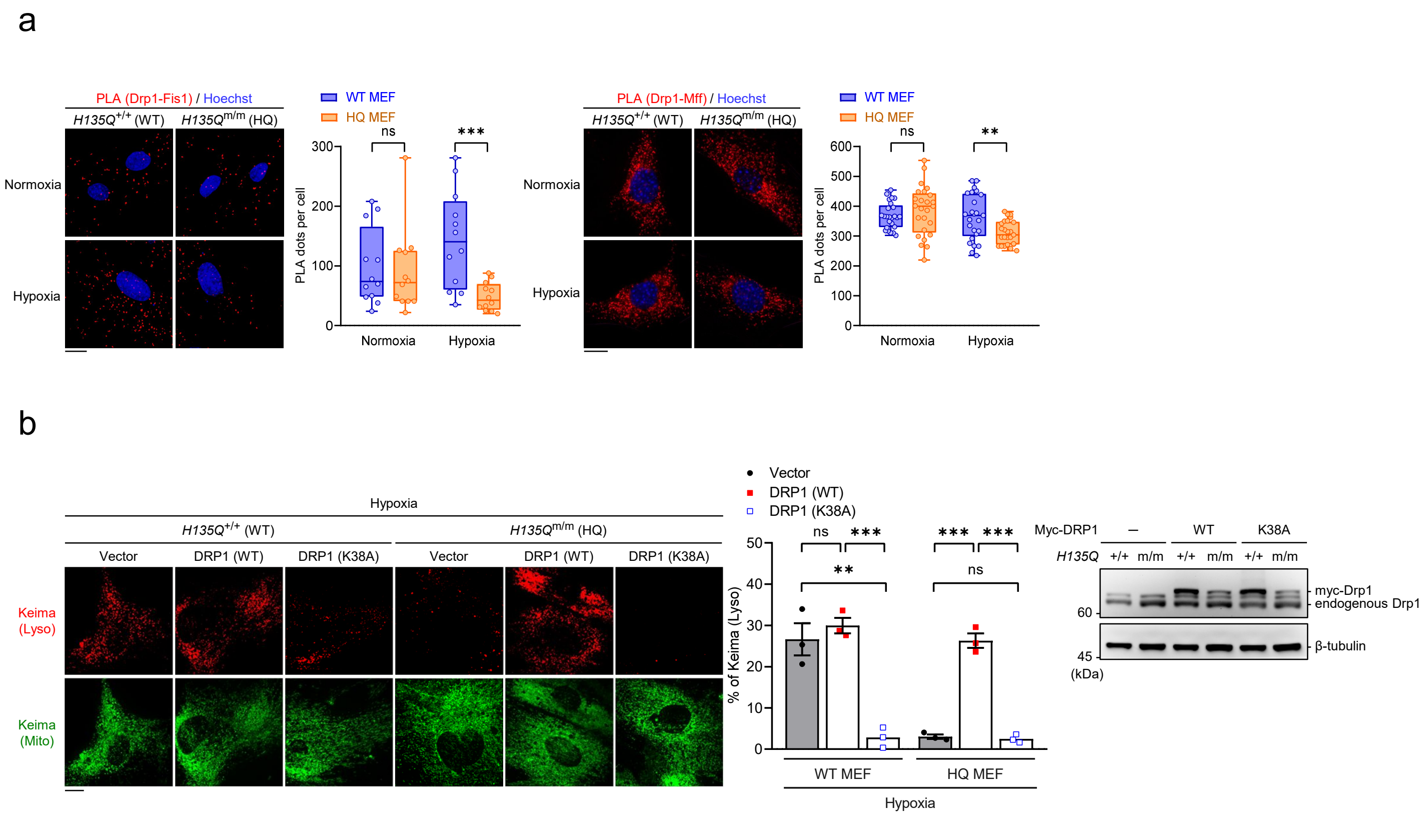

Supplementary Figure S4. DRP1-fission receptor interaction and DRP1 overexpression effect in NME3-defective cells. (a) *H135Q*$^{+/+}$ (WT) and *H135Q*$^{m/m}$ (HQ) MEFs were incubated in normoxia and hypoxia 24 h for PLA of Drp1-Fis1 and Drp1-Mff interaction. Representative images, scale bar, 20 μm and quantitation data of PLA dots per cell are shown (For Drp1-Fis1 interaction, 12 cells were counted, n = 3; for Drp1-Mff1 interaction, 25 cells were counted, n = 3). **(b)** *H135Q*$^{+/+}$ (WT) and *H135Q*$^{m/m}$ (HQ) MEFs stably expressing mt-Keima were transfected with control, Myc-DRP1 and DRP1 (K38A) vectors. Cells were incubated in hypoxia for 24 h before mt-Keima analysis. Representative images and quantification of lysosomal Keima are shown (30 cells were counted, n = 3). Scale bar, 20 μm. *Right* shows the Western blot for the expression of Myc-DRP1 plasmids. NS means no significant, *p < 0.05, **p < 0.01, ***p < 0.001; two-tailed t test.

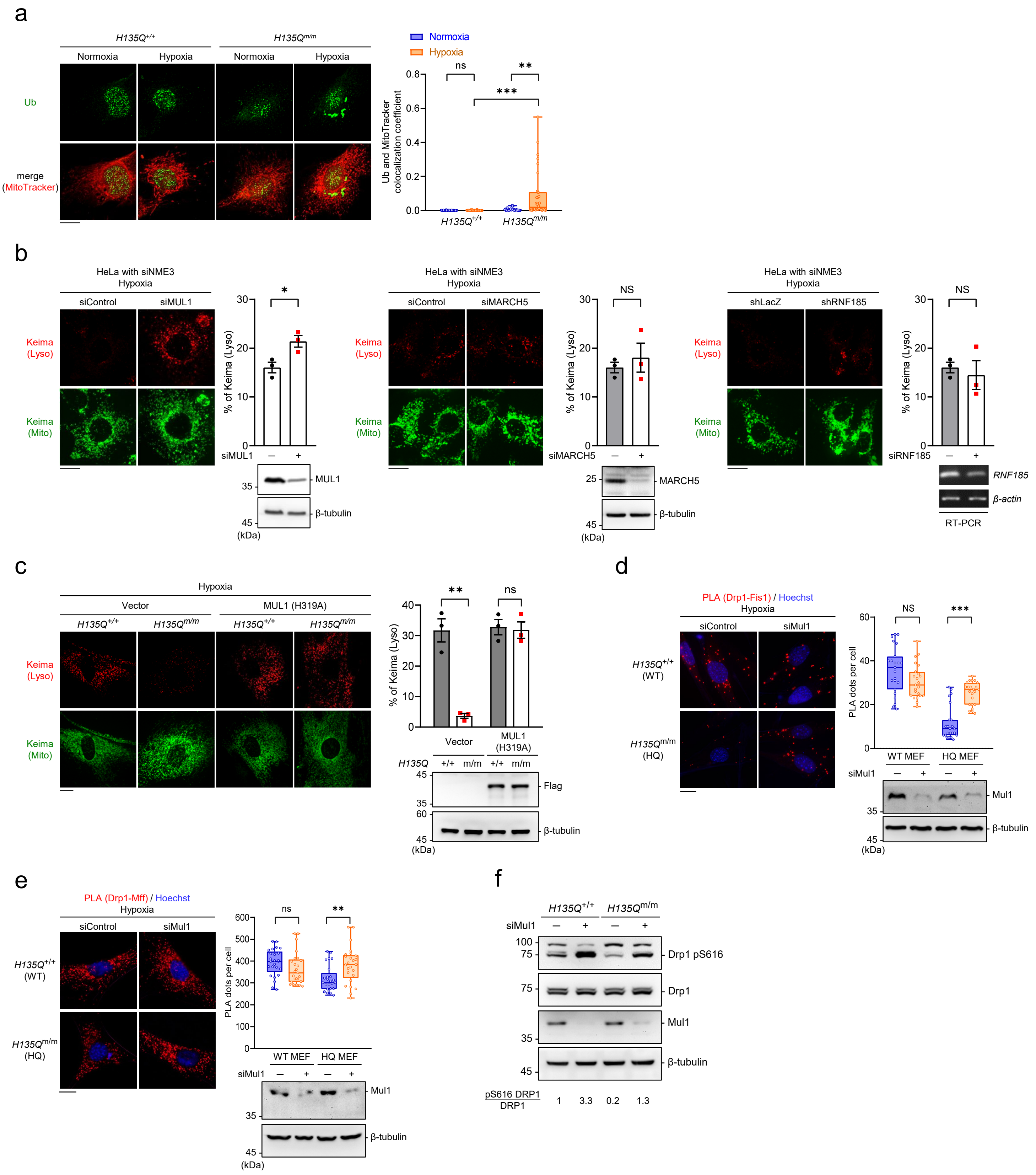

Supplementary Figure S5. Mitophagy and active DRP1 in NME3-defective cells are regulated by MUL1 in hypoxia.
(a) MEFs were incubated in normoxia vs hypoxia for 24 h. Cells were stained with MitoTracker Red and fixed for immunofluorescence staining of endogenous ubiquitin (Ub) by anti-ubiquitin FK2 monoclonal antibody followed by confocal microscopy analysis. (*Left*) Representative images are shown, scale bar, 20 μm. (*Right*) Colocalization coefficient of Ub and MitoTracker Red were shown as mean ± SEM (≥100 cells were analyzed). **(b)** HeLa cells expressing mt-Keima were transfected with NME3 siRNA in combination with MUL1 siRNA, MARCH5 siRNA, or RNF185 siRNA. These cells were incubated in hypoxia chamber with 0.5% oxygen 24 h for confocal analysis of mt-Keima. Scale bar, 20 μm. The percentages of lysosomal Keima of cells are shown (n = 3 independent experiments). **(c)** *H135Q*$^{+/+}$ and *H135Q*$^{m/m}$ MEFs expressing mt-Keima were transfected with control vector and Flag-MUL1 (H319A) catalytic dead mutant. Following hypoxia for 24 h, lysosomal and total Keima fluorescence were analyzed. Representative images and quantitation data are shown (30 cells were counted, n = 3). Scale bar, 20 μm. **(d-e)** WT- and HQ-MEFs transfected with control and Mul1 siRNA were used for performing PLA of **(d)** Drp1-Fis1, and **(e)** Drp1-Mff interaction. Scale bar, 20 μm. Representative images and quantitation data of PLA dots per cell are shown (n = 23 cells for Drp1-Fis1, and n = 25 for Drp1-Mff). **(f)** Western blot of pS616-Drp1, total Drp1, Mul1 and β-tubulin. NS means no significant, *$p < 0.05$, **$p < 0.01$, ***$p < 0.001$; two-tailed *t* test.

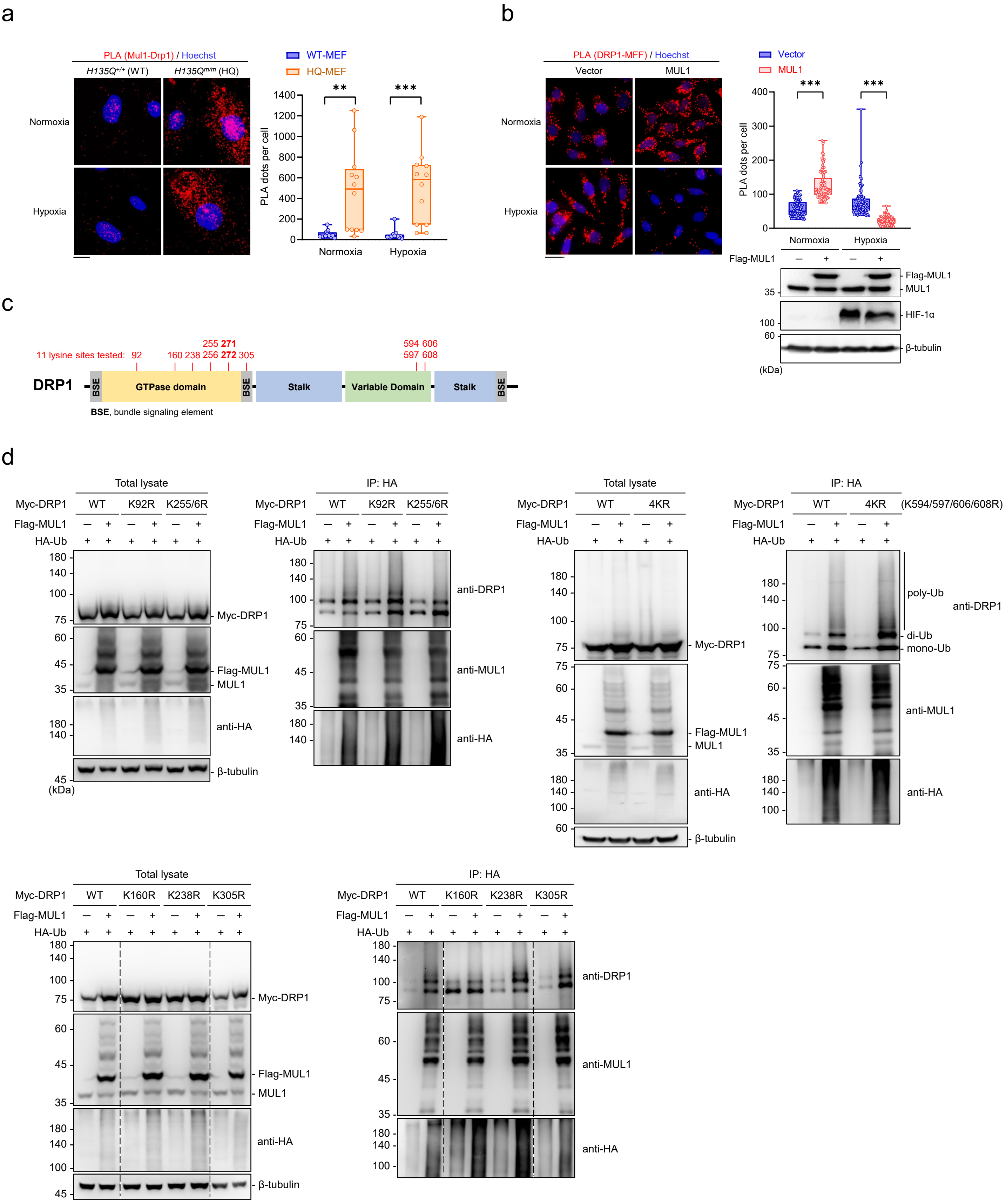

a
PLA (Mul1-Drp1) / Hoechst
H135Q+/+ (WT)
H135Qm/m (HQ)
Normoxia
Hypoxia
WT-MEF
HQ-MEF
PLA dots per cell
0
200
400
600
800
1000
1200
1400
**
***
Normoxia
Hypoxia
b
PLA (DRP1-MFF) / Hoechst
Vector
MUL1
Normoxia
Hypoxia
Vector
MUL1
PLA dots per cell
0
100
200
300
400
***
***
Normoxia
Hypoxia
Flag-MUL1
Flag-MUL1
MUL1
35
HIF-1α
100
60
β-tubulin
(kDa)
c
11 lysine sites tested: 92
160 238 255 256 271 272 305
594 597 606 608
DRP1
BSE
GTPase domain
BSE
Stalk
Variable Domain
Stalk
BSE
BSE, bundle signaling element
d
Total lysate
IP: HA
Myc-DRP1
WT
K92R
K255/6R
Flag-MUL1
HA-Ub
180
140
100
75
60
45
35
Myc-DRP1
Flag-MUL1
MUL1
anti-HA
β-tubulin
(kDa)
anti-DRP1
anti-MUL1
anti-HA
4KR
(K594/597/606/608R)
poly-Ub
di-Ub
mono-Ub
anti-DRP1
K160R
K238R
K305R

e

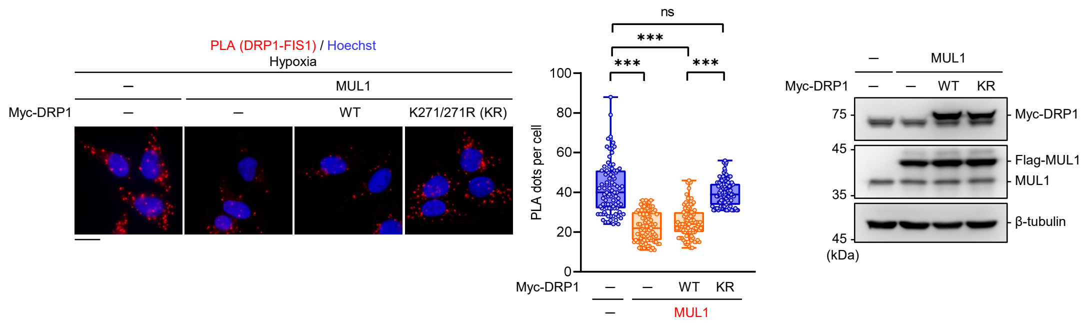

Supplementary Figure S6. MUL1 ubiquitinates wild-type and lysine-mutated DRP1 variants.

(a) *H135Q*$^{+/+}$ (WT) and *H135Q*$^{m/m}$ (HQ) MEFs were incubated in hypoxia for 24 h, followed by PLA using Mul1 and Drp1 antibodies. Representative images and the number of PLA dots per cell were quantified (12 cells were analyzed). Scale bar, 20 µm. **(b)** PLA signal of DRP1-MFF interaction. HeLa cells transfected with Flag-MUL1 or empty vector were treated normoxia and hypoxia for 24 h, scale bar, 20 µm. Quantitation of PLA foci in each cell are shown (n = 100 cells). **(c)** Diagram of human DRP1 domains and the lysine mutation sites of DRP1 variants generated in this study. **(d)** 293T cells were co-transfected with Flag-MUL1, DRP1 variants and HA-ubiquitin (Ub). Cell lysates were harvested in SDS-PAGE loading buffer. After heating at 95ºC, lysates were 10-fold diluted with TE buffer for HA-beads pulldown analysis. Western blots of total lysates and HA-pulldown using antibodies of DRP1, MUL1 and HA are shown. **(e)** HeLa cells were transfected with MUL1 and WT or K271/272R (KR) mutant of Myc-DRP1 for 1 d. After incubated in hypoxia for 24 h, cells were fixed for PLA of DRP1-FIS1 interaction. Representative images and the number of PLA dots per cell were shown as mean ± SEM (90 cells were counted from 3 independent experiments). Scale bar, 20 µm. *Right* shows the Western blot for the expression of Flag-MUL1 and Myc-DRP1. NS means no significant, **p < 0.01, ***p < 0.001; two-tailed t test.

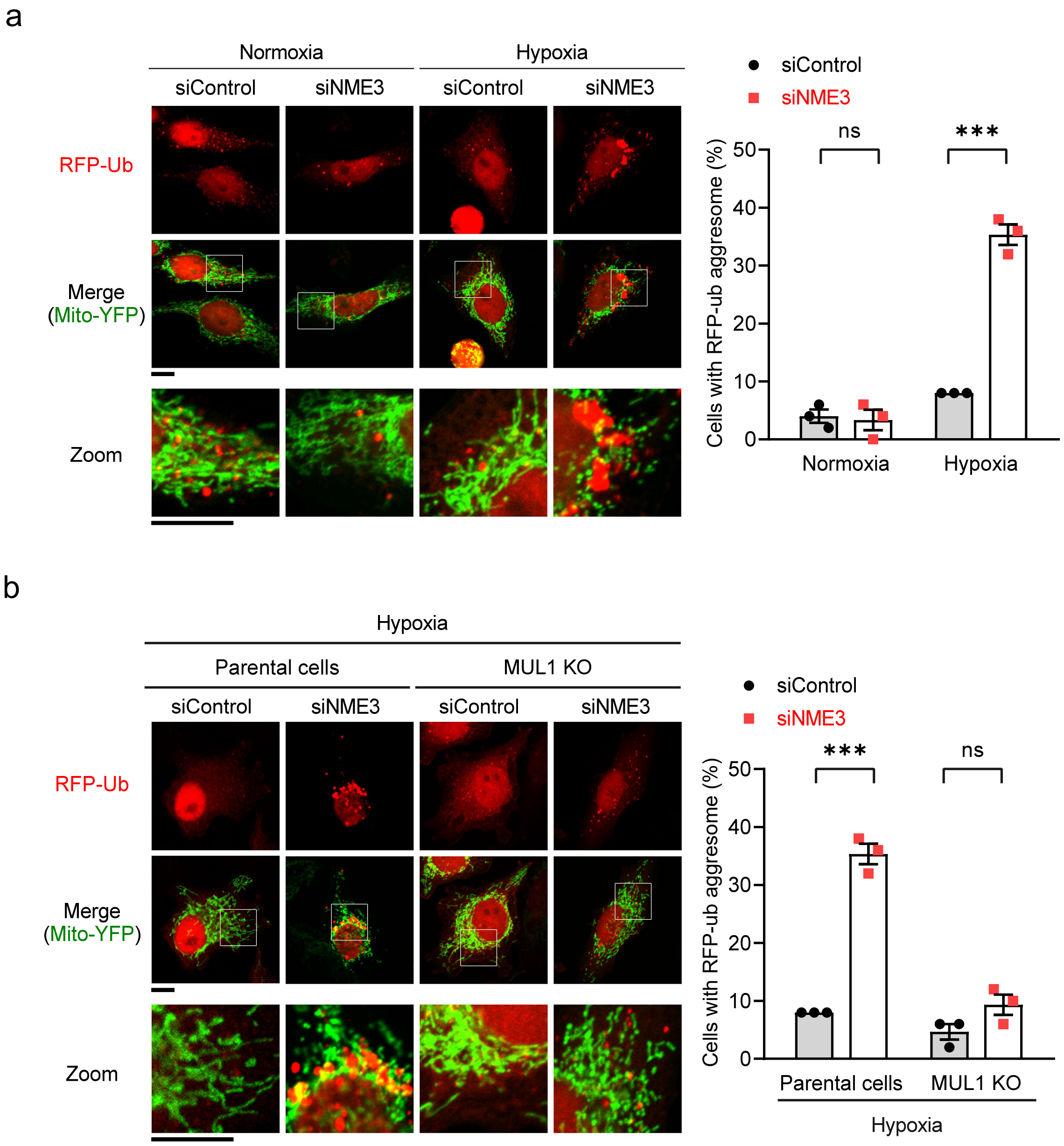

Supplementary Figure S7. MUL1 contributes to mitochondrial ubiquitination in NME3 knockdown cells.
(a) HeLa cells were transfected with control and NME3 siRNA together with RFP-ubiquitin (Ub) and Mito-YFP plasmids. **(b)** Control and MUL1 knockout (KO) HeLa cells were transfected with control and NME3 siRNA together with RFP-Ub and Mito-YFP plasmids. **(a, b)** After incubation in normoxia and hypoxia (0.5% oxygen) for 24 h, cells were fixed for confocal microscopy analysis. Scale bar, 10 µm. (*Left*) Representative images are shown and (*Right*) the percentage of cells containing RFP-Ub aggregates with mitochondria are shown (≥100 cells, n = 3). NS means no significant, ****p* < 0.001; two-tailed *t* test.

Supplementary Table 1

REAGENT	SOURCE	IDENTIFIER & DILUTION
**Antibodies**		
Beta-actin	Sigma-Aldrich	Cat# A5441<br>AB_476744<br>1:2000
Beta-tubulin	Sigma-Aldrich	Cat# T4026<br>AB_477577<br>1:2000
BNIP3	Genetex	Cat# gtx10433<br>AB_381039<br>1:1000
DRP1	Cell Signaling Technology	Cat# 8570<br>AB_10950498<br>1:1000
DRP1	Abcam	Cat# ab56788<br>AB_941306<br>1:1000
FIS1	Proteintech	Cat# 10956-1-AP<br>AB_2102532<br>1:1000
Flag tag	Sigma-Aldrich	Cat# F3165<br>AB_259529<br>1:2000
FUNDC1	Genetex	Cat# gtx45570<br>AB_11167069<br>1:1000
GAPDH	Genetex	Cat# gtx28245<br>AB_370675<br>1:1000
GFP	Abclonal	Cat# AE012<br>AB_2770402<br>1:1000
HA tag	Biolegend	Cat# 902301<br>AB_2565018<br>1:1000
HIF-1α	Cell Signaling Technology	Cat# 36169<br>AB_2799095<br>1:1000
His tag	Clontech	Cat# 631212<br>AB_2721905<br>1:1000
LC3B	Cell Signaling Technology	Cat# 3868<br>AB_2137707<br>1:1000
LC3B	Cell Signaling Technology	Cat# 83506<br>AB_2800018<br>1:1000

MARCH5	Millipore	Cat# 06-1036<br>AB_10807027<br>1:1000
MFF	Proteintech	Cat# 17090-1-AP<br>AB_2142463<br>1:1000
MFN1	Proteintech	Cat# 13798-1-AP<br>AB_2266318<br>1:1000
MFN2	Abcam	Cat# ab56889<br>AB_2142629<br>1:1000
MID49	Proteintech	Cat# 16413-1-AP<br>AB_2714217<br>1:2000
MID51	Proteintech	Cat# 20164-1-AP<br>AB_10639522<br>1:2000
MUL1	Abcam	Cat# ab209263<br>N/A<br>1:1000
MUL1	Proteintech	Cat# 16133-1-AP<br>AB_2147111<br>1:1000
N1-Phosphohistidine (1-pHis)	Millipore	Cat# MABS1341<br>N/A<br>1:1000
N3-Phosphohistidine (3-pHis)	Salk Institute	clone SC44-1<br>N/A<br>1:1000
NIX (BNIP3L)	Proteintech	Cat# 12986-1-AP<br>AB_2877901<br>1:1000
NME1	Santa Cruz Biotechnology	Cat# sc-343<br>AB_632060<br>1:1000
NME2	Santa Cruz Biotechnology	Cat# sc-14790<br>AB_2267261<br>1:1000
NME4	Genetex	Cat# gtx55728<br>N/A<br>1:1000
TOM20	Cell Signaling Technology	Cat# 42406<br>AB_2687663<br>1:1000
TOM20	Abcam	Cat# ab56783<br>AB_945896<br>1:1000
Ubiquitin	Millipore	Cat# 04-263<br>AB_612093<br>1:1000

Western blot secondary antibody: anti-mouse IgG-HRP	Millipore	Cat# AP-124P<br>AB_90456<br>1:7000-1:10000
Western blot secondary antibody: anti-rabbit IgG-HRP	Millipore	Cat# AP-132P<br>AB_90264<br>1:7000-1:10000
Western blot secondary antibody: anti-goat IgG-HRP	Santa Cruz Biotechnology	Cat# sc-2020<br>AB_631728<br>1:7000-1:10000
Immunofluorescence (IF) staining: LC3B	Cell Signaling Technology	Cat# 3868<br>AB_2137707<br>1:800
IF staining: TOM20	Abcam	Cat# ab56783<br>AB_945896<br>1:400
IF staining: Ubiquitin	Millipore	Cat# 04-263<br>AB_612093<br>1:400
FITC-conjugated goat anti-mouse IgG	Sigma-Aldrich	Cat# F9006<br>AB_259787<br>1:100
FITC-conjugated goat anti-rabbit IgG	Sigma-Aldrich	Cat# F9887<br>AB_259816<br>1:100
TRITC-conjugated goat anti-mouse IgG	Sigma-Aldrich	Cat# T5393<br>AB_261699<br>1:200
TRITC-conjugated goat anti-rabbit IgG	Sigma-Aldrich	Cat# T5268<br>AB_261693<br>1:200
(Immunoprecipitation antibody) Myc tag	Millipore	Cat#05-419<br>AB_309725
(Immunoprecipitation antibody) Normonal mouse IgG	Santa Cruz Biotechnology	Cat#sc2025<br>AB_737182
(Proximity ligation assay antibody) DRP1	Abcam	Cat# ab56788<br>AB_941306<br>1:200-1:400
(Proximity ligation assay antibody) FIS1	Proteintech	Cat# 10956-1-AP<br>AB_2102532<br>1:200-1:400
(Proximity ligation assay antibody) MFF	Proteintech	Cat# 17090-1-AP<br>AB_2142463<br>1:200
(Proximity ligation assay antibody) HA	Genetex	Cat# gtx628489<br>AB_2888042<br>1:200
(Proximity ligation assay antibody) MUL1	Abcam	Cat# ab209263<br>N/A<br>1:200-1:400

**Chemicals, peptides, and recombinant proteins**		
2,3,5-triphenyl-tetrazolium chloride (TTC Red)	Sigma-Aldrich	T8877
Alexa Fluor 488 Phalloidin	ThermoFisher Scientific	A12379
Chloroquine diphosphate salt (CQ)	Sigma-Aldrich	C6628
Crystal Violet	Sigma-Aldrich	C6158
Evans blue	Sigma-Aldrich	E2129
Hoechst 33342	Invitrogen	H1399
mitoTEMPO	Sigma-Aldrich	SML0737
MitoTracker Red CMXRos	ThermoFisher Scientific	M7512
Myc peptide	ChromoTek	Yp-1
N-Acetyl-L-cysteine (NAC)	Sigma-Aldrich	A9165
Tetramethylrhodamine ethyl ester perchlorate (TMRE)	Sigma-Aldrich	87917
z-Leu-Leu-Leu-al (MG132)	Sigma-Aldrich	C2211
**Critical commercial assays**		
Duolink In Situ PLA Detection Reagent Red	Sigma-Aldrich	DUO92008
Duolink In Situ PLA Probe anti-mouse PLUS	Sigma-Aldrich	DUO92001 (RRID:AB_2810939)
Duolink In Situ PLA Probe anti-rabbit MINUS	Sigma-Aldrich	DUO92005 (RRID:AB_2810942)
GFP-Trap Magnetic Agarose	ChromoTek	gtma-20 (RRID: AB_2631358)
Glutathione Sepharose 4B	GE Healthcare	17-0756-01
Mitochondria isolation kit	ThermoFisher Scientific	89874
Mitochondria isolation kit for mouse tissue	Miltenyi Biotec (MACS)	130-096-946
Mouse anti-Tom22 microbeads	Miltenyi Biotec (MACS)	130-127-693
Mitochondria isolation kit, human	Miltenyi Biotec (MACS)	130-094-532
Monoclonal Anti-HA–Agarose	Sigma-Aldrich	A2095 (mouse mAb, clone HA-7, RRID:AB_257974)
Myc-Trap Magnetic Agarose	ChromoTek	ytma-20 (RRID: AB_2631370)
Ni-NTA Agarose	Qiagen	1018244
QuikChange II XL Site-Directed Mutagenesis Kit	Agilent	200522

**Experimental models: Cell lines**		
Human: 293T cells	ATCC	CRL-3216
Human: HeLa cells	ATCC	CCL-2
Human: F741 frbroblasts	Hanna Mandel (Institute of Human Genetics, Galilee Medical Center, Nahariya, Israel)	Proc Natl Acad Sci USA. 2019 Jan 8;116(2):566-574.
Mouse: MEF cells DRP1 +/+	Gift from Michael Ryan (Monash University, Melbourne, Victoria, AU)	N/A
Mouse: MEF cells DRP1 -/-	Gift from Michael Ryan (Monash University, Melbourne, Victoria, AU)	N/A
Mouse: MEF cells Nme3 H135Q +/+	This article	N/A
Mouse: MEF cells Nme3 H135Q m/m	This article	N/A
Mouse: MEF cells Nme3 +/+	This article	PLoS Genet. 2012;8(3):e1002567.
Mouse: MEF cells Nme3 -/-	This article	PLoS Genet. 2012;8(3):e1002567.
**Experimental models: Organisms/strains**		
Mouse: C57Bl/6J	National Taiwan University College of Medicine Laboratory Animal Center	N/A
Mice: Nme3 H135Q	Transgenic Mouse Models Core Facility (National Taiwan University, Taiwan)	"This article
**Oligonucleotides**		
Human NME3 (NM_002513.2) RT-PCR primers<br>5'-GCTGGTGGGCGAGATTGT-3'<br>5'-TTCAGCGCCACCAACTTGA-3'	Published reference	Int J Mol Sci. 2020 Jul 17;21(14):5048.
Human GAPDH (NM_002046.7) RT-PCR primers<br>5'-CATGGCACCGTCAAGG-3'<br>5'-CACCATGGGGGCATCAGC-3'	Published reference	Biochem J. 2016 May 1;473(9):1237-45.

Human RNF185 (NM_152267.4) RT-PCR primers<br>CTGTCACGCCTCTTCCTATTTGT<br>GCCCAGCATTAGGCAATCAG	This article	N/A
Human beta-actin (NM_001101.3) RT-PCR primers<br>GCACCACACCTTCTACAATGA<br>GTAGCACAGCTTCTCCTTAAT	This article	N/A
**Plasmids**		
pHAGE-mt-mKeima	Addgene	131626
pMRX-GFP-LC3-RFP-DLC3	Addgene	84572
MitoYFP	Gift from Wei Yuan Yang (Academia Sinica, Taiwan)	N/A
RFP-ubiquitin	Gift from Ruey-Hwa Chen (Academia Sinica, Taiwan)	N/A
HA-ubiquitin	Gift from Ruey-Hwa Chen (Academia Sinica, Taiwan)	N/A
pCMV2-Flag vector	Published reference	Biochem J. 2016 May 1;473(9):1237-45.
pCMV2-Flag-NME3	Published reference	Biochem J. 2016 May 1;473(9):1237-45.
pET-28m-6xHis-thrombin-NME3 (WT)	Published reference	Proc Natl Acad Sci USA. 2019 Jan 8;116(2):566-574.
pET-28m-6xHis-thrombin-NME3 (H135Q)	Published reference	Proc Natl Acad Sci USA. 2019 Jan 8;116(2):566-574.
pET-28m-6xHis-thrombin-NME3 (E40/46D)	Published reference	Proc Natl Acad Sci USA. 2019 Jan 8;116(2):566-574.
pLAS-pPuro-pTK vector	This article	N/A
pLAS-pPuro-pTK-Flag-hNME3 (WT) (Resistant to NME3 shRNA, TRCN0000037747)	This article	N/A
pLAS-pPuro-pTK-Flag-hNME3 (H135Q) (Resistant to NME3 shRNA, TRCN0000037747)	This article	N/A
pLAS-pPuro-pTK-Flag-hNME3 (E40/46D) (Resistant to NME3 shRNA, TRCN0000037747)	This article	N/A

pLAS2w.pPuro.pTK-Flag-WT-NME3-HA	This article	N/A
pLAS2w.pPuro.pTK-Flag-TOM20-NΔ-NME3-HA	This article	N/A
pRK5-Flag-wild-type lipid 1	Addgene	32005
pRK5-Flag- catalytic-dead lipid 1	Addgene	32006
pEGFP-C1-Raf1-PABD	Published reference	J Biol Chem. 2000 Aug 4;275(31):23911-8.
pET30a-Myc-DRP1	This article	N/A
pCMV2-Flag-MUL1 (WT)	This article	N/A
pCMV2-Flag-MUL1 (H319A)	This article	N/A
pGEX-4T-1-MUL1 (WT)	This article	N/A
pGEX-4T-1-MUL1 (H319A)	This article	N/A
pGW1-Myc-DRP1 (WT)	Gift from Chuang-Rung Chang (National Tsing Hua University, Taiwan)	J Biol Chem. 2004 Aug 20;279(34):35967-74.
pGW1-Myc-DRP1 (K38A)	This article	N/A
pGW1-Myc-DRP1 (K92R)	This article	N/A
pGW1-Myc-DRP1 (K160R)	This article	N/A
pGW1-Myc-DRP1 (K238R)	This article	N/A
pGW1-Myc-DRP1 (K255/256R)	This article	N/A
pGW1-Myc-DRP1 (K271/272R)	This article	N/A
pGW1-Myc-DRP1 (K305R)	This article	N/A
pGW1-Myc-DRP1 (K594/597/606/608R)	This article	N/A
pCMVdeltaR8.91	RNA Technology Platforms and Gene Manipulation Core Facility (Taipei, Taiwan)	C6-6-1
pMD.G	RNA Technology Platforms and Gene Manipulation Core Facility (Taipei, Taiwan)	C6-6-1
Control shRNA to LacZ	RNA Technology Platforms and Gene Manipulation Core Facility (Taipei, Taiwan)	TRCN0000072224
Human NEM3 shRNA	RNA Technology Platforms and Gene Manipulation Core Facility (Taipei, Taiwan)	TRCN0000037747
Control siRNA	Dharmacon	D-001210-02-20
Human NME3 siRNA	Dharmacon	L-006753-00-0005
Human DRP1 siRNA	Dharmacon	M-012092-01-0005
Human FUNDC1 siRNA	Dharmacon	M-018480-01-0005
Human MARCH5 siRNA	Dharmacon	M-007001-01-0005
Human RNF185 siRNA	Dharmacon	M-007107-01-0005

Human MUL1 siRNA	Dharmacon	M-007062-02-0005
Mouse Mul1 siRNA	Dharmacon	M-050675-00-0010
mCherry-Cas9	This article	Gene Knockout/in Cell Line Modeling Core (College of Medicine, National Taiwan University, Taiwan)
pUC19-hNME3-EGFP-KI	This article	Gene Knockout/in Cell Line Modeling Core (College of Medicine, National Taiwan University, Taiwan)
pUC19-NME3-HA-P2A-ZEO-KI	This article	Gene Knockout/in Cell Line Modeling Core (College of Medicine, National Taiwan University, Taiwan)
lentiCRISPR v2-hMUL1 sgRNA2 (5'-GTACTCCGTGTACCGGCAGA-3')	This article	Gene Knockout/in Cell Line Modeling Core (College of Medicine, National Taiwan University, Taiwan)
lentiCRISPR v2-hNME3 sgRNA2 (5'-CTT CGC TAA CCT CTT CCC CG-3')	This article	Gene Knockout/in Cell Line Modeling Core (College of Medicine, National Taiwan University, Taiwan)
**Software and algorithms**		
GraphPad Prism 8	GraphPad Software	https://www.graphpad.com/scientificsoftware/prism/
ZEN	Carl Zeiss	ZEN BLUE, v2009
AxioVision	Carl Zeiss	Rel. 4.8
AxioObserver	Carl Zeiss	A1
Image J	National Institutes Health (NIH) software	1.52p
Proteome Discoverer	Thermo Fisher Scientific	v2.5
SEQUEST	SwissProt database	v2022-10-12
Imaris software	Bitplane, United Kingdom	
