# Supplementary material for: NME3 is a gatekeeper for DRP1-dependent mitophagy in hypoxia
Source: Nat Commun. 2024 Mar 13;15:2264. doi: 10.1038/s41467-024-46385-7 (PMC10938004; doi:10.1038/s41467-024-46385-7)
Supplement: Supplementary file 3 — Description of Additional Supplementary Files [file 41467_2024_46385_MOESM3_ESM.pdf]

File name: Supplementary Movie 1
Description: A 9 month-old *Nme3* WT mouse walked along the cage’s ledge.

File name: Supplementary Movie 2
Description: A 9 month-old *Nme3* H135Q mouse walked along the cage’s ledge.
