# Supplementary material for: NME3 is a gatekeeper for DRP1-dependent mitophagy in hypoxia
Source: Nat Commun. 2024 Mar 13;15:2264. doi: 10.1038/s41467-024-46385-7 (PMC10938004; doi:10.1038/s41467-024-46385-7)
Supplement: Supplementary file 6 — Reporting Summary [file 41467_2024_46385_MOESM6_ESM.pdf]

Corresponding author(s): Zee-Fen CHANG

Last updated by author(s): 2024-2-7

Reporting Summary

Nature Portfolio wishes to improve the reproducibility of the work that we publish. This form provides structure for consistency and transparency in reporting. For further information on Nature Portfolio policies, see our Editorial Policies and the Editorial Policy Checklist.

Statistics

For all statistical analyses, confirm that the following items are present in the figure legend, table legend, main text, or Methods section.

n/a	Confirmed	
☐	☒	The exact sample size (*n*) for each experimental group/condition, given as a discrete number and unit of measurement
☐	☒	A statement on whether measurements were taken from distinct samples or whether the same sample was measured repeatedly
☐	☒	The statistical test(s) used AND whether they are one- or two-sided<br>*Only common tests should be described solely by name; describe more complex techniques in the Methods section.*
☒	☐	A description of all covariates tested
☒	☐	A description of any assumptions or corrections, such as tests of normality and adjustment for multiple comparisons
☐	☒	A full description of the statistical parameters including central tendency (e.g. means) or other basic estimates (e.g. regression coefficient) AND variation (e.g. standard deviation) or associated estimates of uncertainty (e.g. confidence intervals)
☐	☒	For null hypothesis testing, the test statistic (e.g. F, t, r) with confidence intervals, effect sizes, degrees of freedom and P value noted<br>*Give P values as exact values whenever suitable.*
☒	☐	For Bayesian analysis, information on the choice of priors and Markov chain Monte Carlo settings
☒	☐	For hierarchical and complex designs, identification of the appropriate level for tests and full reporting of outcomes
☒	☐	Estimates of effect sizes (e.g. Cohen's *d*, Pearson's *r*), indicating how they were calculated

Our web collection on statistics for biologists contains articles on many of the points above.

Software and code

Policy information about availability of computer code

Data collection	Software used for data collection: Immunoblot (VisionWorks, v8.20.17096.9551; Analytik Jena), Real-Time PCR (QuantStudio Real-Time PCR software v1.3; Applied Biosystems), Flow Cytometer (BD FACSDiva, v8.0.1, BD Bioscience), Confocal microscopy LSM880 with AiryScan detetector, LSM780, and Spinning Disc (ZEN BLUE, v2009; Carl Zeiss), Fluorescence microscopy (AxioVision, Rel. 4.8; Carl Zeiss), and Fluorescence microscopy (AxioObserver, v.A1; Carl Zeiss).
Data analysis	Software used for data analysis: GraphPad Prism 8 (GraphPad Software), Image J (1.52p; National Institutes Health (NIH) software), Proteome Discoverer (v2.5; Thermo Fisher Scientific), SEQUEST (v2022-10-12; SwissProt database).

For manuscripts utilizing custom algorithms or software that are central to the research but not yet described in published literature, software must be made available to editors and reviewers. We strongly encourage code deposition in a community repository (e.g. GitHub). See the Nature Portfolio guidelines for submitting code & software for further information.

Data

Policy information about availability of data

All manuscripts must include a data availability statement. This statement should provide the following information, where applicable:

- Accession codes, unique identifiers, or web links for publicly available datasets
- A description of any restrictions on data availability
- For clinical datasets or third party data, please ensure that the statement adheres to our policy

The Data Availability section is provided with this paper. Materials and their source are provided Supplementary Table 1. The unprocessed images of Western blots are provided in Supplementary Data file. Source data, statistic results, and exact p values of all Figures and Supplementary Figures are provided in Supplementary Data File.

Research involving human participants, their data, or biological material

Policy information about studies with human participants or human data. See also policy information about sex, gender (identity/presentation), and sexual orientation and race, ethnicity and racism.

Reporting on sex and gender	Primary fibroblast (F741) from 1 female patient was analyzed.
Reporting on race, ethnicity, or other socially relevant groupings	Human F741 fibroblast was a kindly gift from Hanna Mandel (Institute of Human Genetics, Galilee Medical Center, Nahariya, Israel) published on Proc Natl Acad Sci USA. 2019 Jan 8;116(2):566-574). The NME3 gene DNA sequence of the patient was checked.
Population characteristics	Age at 3-month-old.
Recruitment	The patient fibroblast was received from the source without any biased selection.
Ethics oversight	The use of patient fibroblast were approved by Galilee Medical Center and the Israeli Ministry of Health.

Note that full information on the approval of the study protocol must also be provided in the manuscript.

Field-specific reporting

Please select the one below that is the best fit for your research. If you are not sure, read the appropriate sections before making your selection.

☒ Life sciences ☐ Behavioural & social sciences ☐ Ecological, evolutionary & environmental sciences

For a reference copy of the document with all sections, see nature.com/documents/nr-reporting-summary-flat.pdf

Life sciences study design

All studies must disclose on these points even when the disclosure is negative.

Sample size	The sample size for animal experiments were estimated to ensure statistical analysis analysis according to published reference (Malays J Med Sci. 2017;24:101-105. Sample Size Calculation in Animal Studies Using Resource Equation Approach) and reproducibility of the results. The sample size for other cell experiments was estimated to ensure statistical analysis and reproducibility of the results. Exact sample sizes of each figure are provided in figure legends and the Supplementary Information for source data.
Data exclusions	No data were excluded from the analyses.
Replication	The number of replication were indicated in each figure legend. At least 3 independent experiments perform for most the analysis except for Figure 3a, 3d-e, 5e, 6c, and Supplementary Figure S1c, S3e, S4a, S5d-e, and S6a only one independent experiment was performed.
Randomization	The mice for ischemia/reperfusion experiments, the Ledge test, or the blood tests were randomly allocated to experimental groups before the start of the experiment. All samples collected from each individual animal were analyzed together using the same processing and analysis method. For experiments by microscopy analysis, random fields were collected from each sample using the same parameter and analysis. For other experiments, such as Western blotting, cells/samples were collected using the same processing and analysis.
Blinding	The investigator performing the ischemia/reperfusion experiments were blinded during experiment and data acquisition. The same parameter and analysis method was applied to all the animals during experiment, data acquisition, and analysis. For other experiments, the cells/samples were treated and collected using the same processing. The investigator were blinded during experiment, data acquisition, and analysis with the same parameter.

Reporting for specific materials, systems and methods

We require information from authors about some types of materials, experimental systems and methods used in many studies. Here, indicate whether each material, system or method listed is relevant to your study. If you are not sure if a list item applies to your research, read the appropriate section before selecting a response.

Materials & experimental systems

n/a	Involved in the study
☐	☒ Antibodies
☐	☒ Eukaryotic cell lines
☒	☐ Palaeontology and archaeology
☐	☒ Animals and other organisms
☒	☐ Clinical data
☒	☐ Dual use research of concern
☒	☐ Plants

Methods

n/a	Involved in the study
☒	☐ ChIP-seq
☐	☒ Flow cytometry
☒	☐ MRI-based neuroimaging

Antibodies

Antibodies used

Western blot primary antibodies: Beta-actin (Sigma-Aldrich, Cat# A5441), Beta-tubulin (Sigma-Aldrich, Cat# T4026), BNIP3 (Genetex, Cat# gtx10433), DRP1 (Cell Signaling Technology, Cat# 8570), DRP1 (Abcam, Cat# ab56788), FIS1 (Proteintech, Cat# 10956-1-AP), Flag tag (Sigma-Aldrich, Cat# F3165), FUNDC1 (Genetex, Cat# gtx45570), GAPDH (Genetex, Cat# gtx28245), GFP (Abclonal, Cat# AE012), HA tag (Biolegend, Cat# 902301), HIF-1α (Cell Signaling Technology, Cat# 36169), His tag (Clontech, Cat# 631212), LC3B (Cell Signaling Technology, Cat# 3868), LC3B (Cell Signaling Technology, Cat# 83506), MARCH5 (Millipore, Cat# 06-1036), MFF (Proteintech, Cat# 17090-1-AP), MFN1 (Proteintech, Cat# 13798-1-AP), MFN2 (Abcam, Cat# ab56889), MID49 (Proteintech, Cat# 16413-1-AP), MID51 (Proteintech, Cat# 20164-1-AP), MUL1 (Abcam, Cat# ab209263), MUL1 (Proteintech, Cat# 16133-1-AP), N1-Phosphohistidine (1-pHis) (Millipore, Cat# MABS1341), N3-Phosphohistidine (3-pHis) (Salk Institute, clone SC44-1), NIX (BNIP3L) (Proteintech, Cat# 12986-1-AP), NME1 ((Santa Cruz Biotechnology, Cat# sc-343), NME2 (Santa Cruz Biotechnology, Cat# sc-14790), NME4 (Genetex, Cat# gtx55728), TOM20 (Cell Signaling Technology, Cat# 42406), TOM20 (Abcam, Cat# ab56783), Ubiquitin (Millipore, Cat# 04-263).
Western blot secondary antibodies: anti-mouse IgG-HRP (Millipore, Cat# AP-124P), anti-rabbit IgG-HRP (Millipore, Cat# AP-132P), anti-goat IgG-HRP (Santa Cruz Biotechnology, Cat# sc-2020).
For Immunofluorescence (IF) staining: LC3B (Cell Signaling Technology, Cat# 3868), IF staining: TOM20 (Abcam, Cat# ab56783), IF staining: Ubiquitin (Millipore, Cat# 04-263), FITC-conjugated goat anti-mouse IgG (Sigma-Aldrich, Cat# F9006), FITC-conjugated goat anti-rabbit IgG (Sigma-Aldrich, Cat# F9887), TRITC-conjugated goat anti-mouse IgG (Sigma-Aldrich, Cat# T5393), TRITC-conjugated goat anti-rabbit IgG (Sigma-Aldrich, Cat# T5268).
Antibodies for Immunoprecipitation (IP) antibodies: Myc tag (Millipore, Cat#05-419), Normal mouse IgG (Santa Cruz Biotechnology, Cat#sc2025).
Antibodies for proximity ligation assay antibodies: DRP1 (Abcam, Cat# ab56788), FIS1 (Proteintech, Cat# 10956-1-AP), MFF (Proteintech, Cat# 17090-1-AP), HA (Genetex, Cat# gtx628489), MUL1 (Abcam, Cat# ab209263).
All Western blot primary antibodies were diluted between 1:1000 to 1:3000 for working concentration. For secondary antibodies, the dilution were between 1:5000 to 1:10000. For IF staining, antibodies were diluted 1:100 to 1:800. For PLA experiments, antibodies were diluted 1:100 to 1:400.

Validation

The RRID or source of validation were provided for each antibody: Western blot primary antibodies: Beta-actin (Sigma-Aldrich, Cat# A5441, RRID: AB_476744), Beta-tubulin (Sigma-Aldrich, Cat# T4026, RRID: AB_477577), BNIP3 (Genetex, Cat# gtx10433, RRID: AB_381039), DRP1 (Cell Signaling Technology, Cat# 8570, RRID: AB_10950498), DRP1 (Abcam, Cat# ab56788, RRID: AB_941306), FIS1 (Proteintech, Cat# 10956-1-AP, RRID: AB_2102532), Flag tag (Sigma-Aldrich, Cat# F3165, RRID: AB_259529), FUNDC1 (Genetex, Cat# gtx45570, RRID: AB_11167069), GAPDH (Genetex, Cat# gtx28245, RRID: AB_370675), GFP (Abclonal, Cat# AE012, RRID: AB_2770402), HA tag (Biolegend, Cat# 902301, RRID: AB_2565018), HIF-1α (Cell Signaling Technology, Cat# 36169, RRID: AB_2799095), His tag (Clontech, Cat# 631212, RRID: AB_2721905), LC3B (Cell Signaling Technology, Cat# 3868, RRID: AB_2137707), LC3B (Cell Signaling Technology, Cat# 83506, RRID: AB_2800018), MARCH5 (Millipore, Cat# 06-1036, RRID: AB_10807027), MFF (Proteintech, Cat# 17090-1-AP, RRID: AB_2142463), MFN1 (Proteintech, Cat# 13798-1-AP, RRID: AB_2266318), MFN2 (Abcam, Cat# ab56889, RRID: AB_2142629), MID49 (Proteintech, Cat# 16413-1-AP, RRID: AB_2714217), MID51 (Proteintech, Cat# 20164-1-AP, RRID: AB_10639522), MUL1 (Abcam, Cat# ab209263, validated in this article), MUL1 (Proteintech, Cat# 16133-1-AP, RRID: AB_2147111), N1-Phosphohistidine (1-pHis) (Millipore, Cat# MABS1341, validated by published paper: Cell. 2015 Jul 2;162(1):198-210.), N3-Phosphohistidine (3-pHis) (Salk Institute, clone SC44-1, validated by published paper: Cell. 2015 Jul 2;162(1):198-210.), NIX (BNIP3L) (Proteintech, Cat# 12986-1-AP, RRID: AB_2877901), NME1 ((Santa Cruz Biotechnology, Cat# sc-343, RRID: AB_632060), NME2 (Santa Cruz Biotechnology, Cat# sc-14790, RRID: AB_2267261), NME4 (Genetex, Cat# gtx55728, validated by manufacturer: https://cdn.origene.com/datasheet/ta501114.pdf), TOM20 (Cell Signaling Technology, Cat# 42406, RRID: AB_2687663), TOM20 (Abcam, Cat# ab56783, RRID: AB_945896), Ubiquitin (Millipore, Cat# 04-263, RRID: AB_612093).
Western blot secondary antibodies: anti-mouse IgG-HRP (Millipore, Cat# AP-124P, RRID: AB_90456, https://www.sigmaaldrich.com/TW/en/product/mm/ap124p), anti-rabbit IgG-HRP (Millipore, Cat# AP-132P, RRID: AB_90264, https://www.sigmaaldrich.com/TW/en/product/mm/ap132p), anti-goat IgG-HRP (Santa Cruz Biotechnology, Cat# sc-2020, RRID: AB_631728, https://www.scbt.com/p/donkey-anti-goat-igg-hrp).
For Immunofluorescence (IF) staining: LC3B (Cell Signaling Technology, Cat# 3868, RRID: AB_2137707), IF staining: TOM20 (Abcam, Cat# ab56783, RRID: AB_945896), IF staining: Ubiquitin (Millipore, Cat# 04-263, RRID: AB_612093), FITC-conjugated goat anti-mouse IgG (Sigma-Aldrich, Cat# F9006, RRID: AB_259787), FITC-conjugated goat anti-rabbit IgG (Sigma-Aldrich, Cat# F9887, RRID: AB_259816), TRITC-conjugated goat anti-mouse IgG (Sigma-Aldrich, Cat# T5393, RRID: AB_261699), TRITC-conjugated goat anti-rabbit IgG (Sigma-Aldrich, Cat# T5268, RRID: AB_261693).
Antibodies for Immunoprecipitation (IP) antibodies: Myc tag (Millipore, Cat#05-419, RRID: AB_309725), Normal mouse IgG (Santa Cruz Biotechnology, Cat#sc2025, RRID: AB_737182).
Antibodies for proximity ligation assay antibodies: DRP1 (Abcam, Cat# ab56788, RRID: AB_941306), FIS1 (Proteintech, Cat# 10956-1-

AP, RRID: AB_2102532), MFF (Proteintech, Cat# 17090-1-AP, RRID: AB_2142463), HA (Genetex, Cat# gtx628489, RRID: AB_2888042), MUL1 (Abcam, Cat# ab209263, validated in this article).

Eukaryotic cell lines

Policy information about cell lines and Sex and Gender in Research

Cell line source(s)	Human 293T cells from female (ATCC CRL-3216), and human HeLa cells from female (ATCC CCL-2) were purchased from ATCC. Human female F741 fibroblast was kindly provided from Hanna Mandel (Institute of Human Genetics, Galilee Medical Center, Nahariya, Israel) published on Proc Natl Acad Sci USA. 2019 Jan 8;116(2):566-574). Mouse MEF cells Drp1 +/+ and Drp1 -/- were kindly gifts from Michael Ryan (Monash University, Melbourne, Victoria, AU). The mouse MEF cells Nme3 H135Q +/+ and m/m were established in this article. The mouse: MEF cells Nme3 +/+ and -/- were established and published on PLoS Genet. 2012;8(3):e1002567.
Authentication	Human 293T cells and human HeLa cells were not authenticated. The human F741 fibroblast was provided from Hanna Mandel (Institute of Human Genetics, Galilee Medical Center, Nahariya, Israel) published on Proc Natl Acad Sci USA. 2019 Jan 8;116(2):566-574.). The mouse MEF cells Drp1 +/+ and Drp1 -/- were kindly gifts from Michael Ryan (Monash University, Melbourne, Victoria, AU). The mouse MEF cells Nme3 H135Q +/+ and m/m were established in this article. The mouse: MEF cells Nme3 +/+ and -/- were established and pusblshed on PLoS Genet. 2012;8(3):e1002567.
Mycoplasma contamination	Cells were not contaminated with mycoplasma.
Commonly misidentified lines (See ICLAC register)	No misidentified cell lines were used in this study

Animals and other research organisms

Policy information about studies involving animals; ARRIVE guidelines recommended for reporting animal research, and Sex and Gender in Research

Laboratory animals	Wild-type (Nme3 H135Q+/+) and homozygous knock-in of Nme3 H135Q (H135Qm/m) of C57BL/6J background were used in this study. The mice at age of 8-12 weeks old were subjected to perform ischemia/reperfusion experiments, body weights, and peripheral blood tests. The Ledge tests of mice were performed at age of 9 months old. The mice were housing under a condition of 12 h light and 12 h dark cycle in animal center (8F) in National Taiwan University with normal room temperature between 18-22oC. The mice were housing in a density of 2-5 mice in each cage. The food and fluid were provided sufficiently and clean cages with wooden strips, and cotton sheets were changed weekly.
Wild animals	The study did not involve wild animals.
Reporting on sex	Only male mice were used in this study.
Field-collected samples	The study did not involve samples collected from the field.
Ethics oversight	The animal protocols and experiment procedure were approved by the Institutional Animal Care and Use Committee (IACUC) of National Taiwan University (IACUC#20200030, March 1, 2020 to Feb 28, 2023, and IACUC #20201125, March/1/2021 to March/1/2024). The IACUC chairman, Dr. Huei-Wen Chen, with 19 committees, including Tzong Shyuan Lee, Wen-Pin Chen, Chia-Yi Chang, An-Sheng Lee, Hao-Hueng Chang, Wan-Tseng Hsu, Ching-yu Lin, You-Tzung Chen, Shin-Hong Shiao, Ya-Hui Chuang, Hau-Jie Yau, Shing-Hwa Liu, Hurng-Yi Wang, Kuei-Pin Chung, Feng-Fang Tsai, Min-Shan Tsai, Ying-Cheng Chiang, Hsuan-Ming Huang, and Yi-Jing Huang approved the protocol of animal experiments.

Note that full information on the approval of the study protocol must also be provided in the manuscript.

Flow Cytometry

Plots

Confirm that:

☒ The axis labels state the marker and fluorochrome used (e.g. CD4-FITC).

☒ The axis scales are clearly visible. Include numbers along axes only for bottom left plot of group (a 'group' is an analysis of identical markers).

☒ All plots are contour plots with outliers or pseudocolor plots.

☒ A numerical value for number of cells or percentage (with statistics) is provided.

Methodology

Sample preparation	Cells were transfected with pMRX-IP-GFP-LC3-RFP-LC3DG (Addgene #84572). Cells stably expressing GFP-LC3-RFP-(deletaG)LC3 were sorted by FACS. After starvation for 0, 4, or 16 h in HBSS, these cells were harvested and subjected to flow cytometry analysis.
Instrument	LSRFortessa (BD bioscience)

Software	BD FACSDiva (v.8.0.1, BD Bioscience)
Cell population abundance	Cells with control RFP-LC3(deltaG) signal were gated for the expression of autophagy flux reporter. The distribution of cells for their intensity of RFP-LC3(deltaG) and GFP-LC3 were shown.
Gating strategy	All cells expressing RFP-LC3(deltaG) signal were gated for the expression of autophagy flux reporter. The intensity and distribution for RFP-LC3(deltaG) and GFP-LC3 of the control and NME3 knockdown cells were similar as shown.

☒ Tick this box to confirm that a figure exemplifying the gating strategy is provided in the Supplementary Information.
