# Supplementary material for: NME3 is a gatekeeper for DRP1-dependent mitophagy in hypoxia
Source: Nat Commun. 2024 Mar 13;15:2264. doi: 10.1038/s41467-024-46385-7 (PMC10938004; doi:10.1038/s41467-024-46385-7)

Supplementary Information

NME3 is a gatekeeper for DRP1-dependent mitophagy in hypoxia

Chih-Wei Chen, Chi Su, Chang-Yu Huang, Xuan-Rong Huang, Xiaojing Cuili, Tung Chao, Chun-Hsiang Fan, Cheng-Wei Ting, Yi-Wei Tsai, Kai-Chien Yang, Ti-Yen Yeh, Sung-Tsang Hsieh, Yi-Ju Chen, Yuxi Feng, Tony Hunter, and Zee-Fen Chang*

*Correspondence to: Zee-Fen Chang (zfchang@ntu.edu.tw)

This includes: unprocessed Western blots of Figure 1-8, and Supplementary Figure S1 to S7

Unprocessed Western blots of Figure 1a

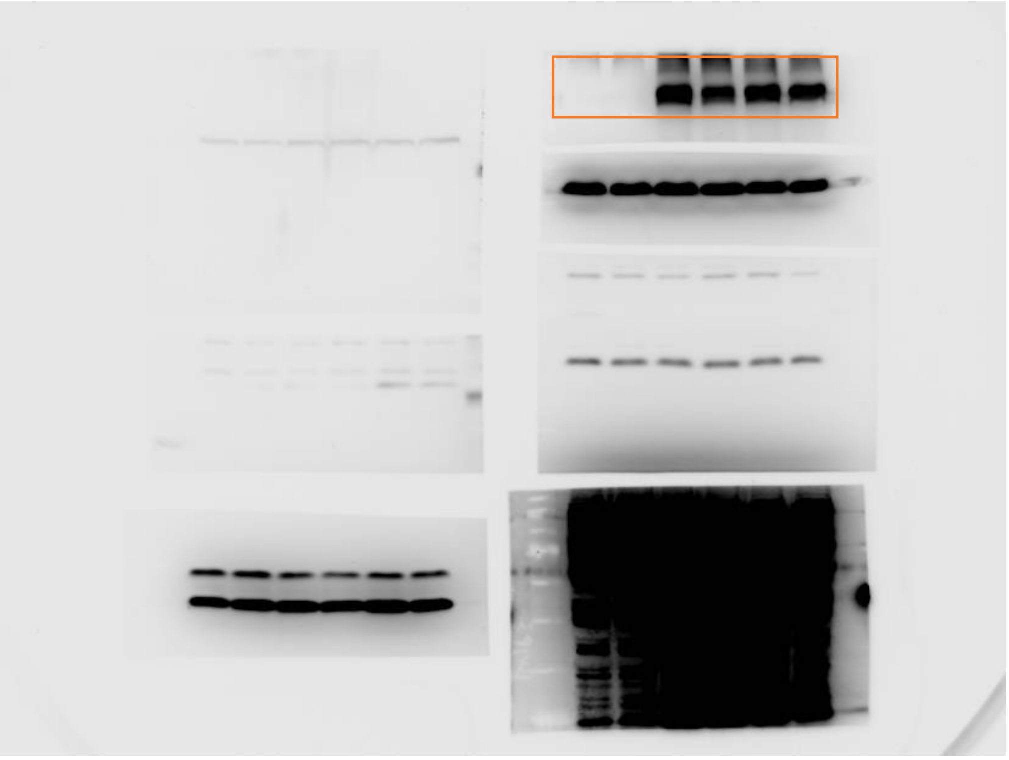

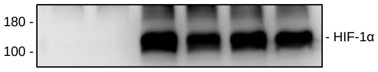

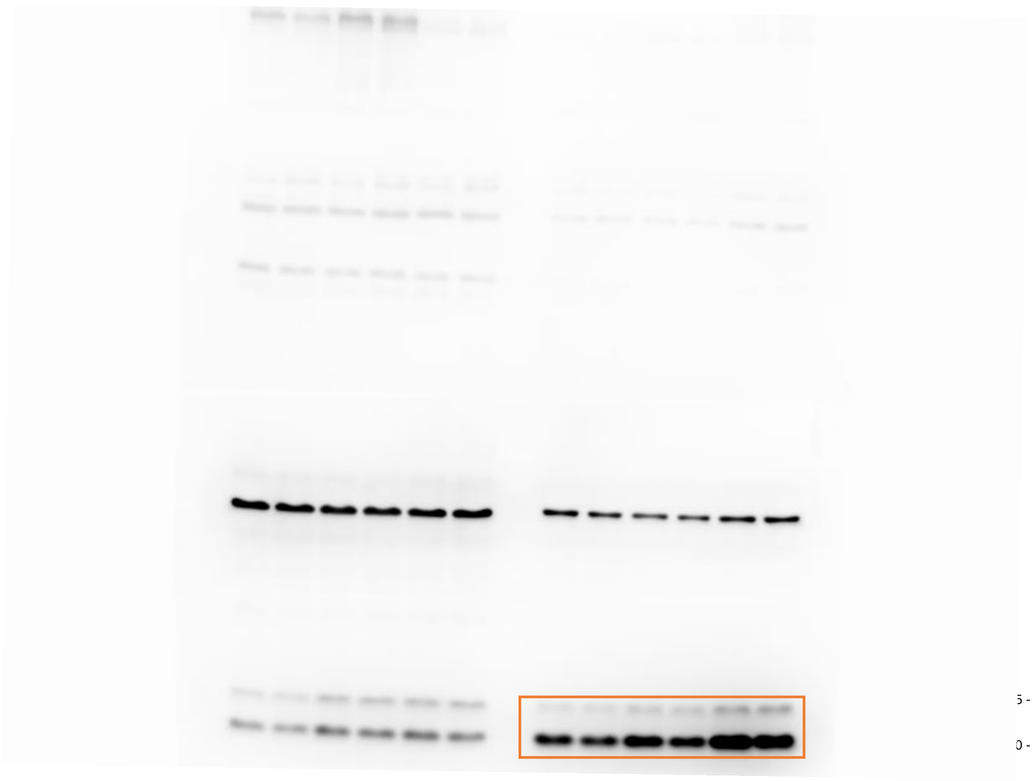

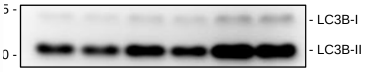

Unprocessed Western blots of Figure 1a (continue)

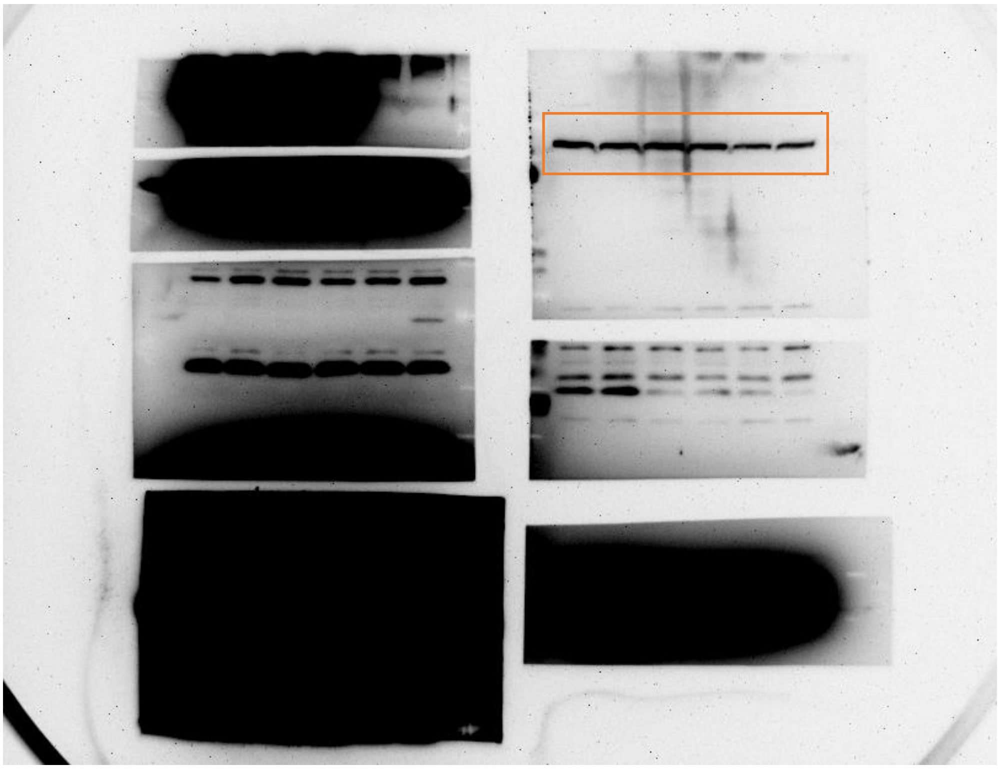

75 -
60 -

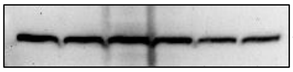

- NIX

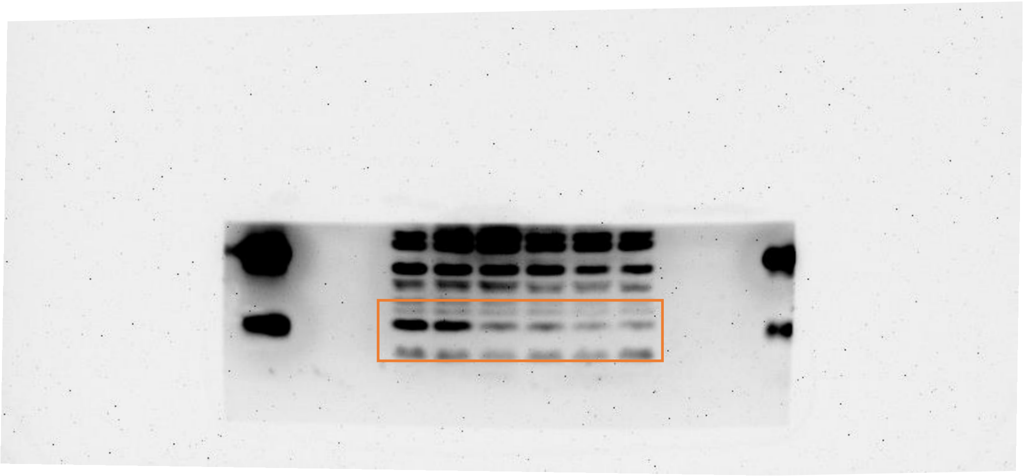

15 -

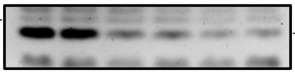

- FUNDC1

Unprocessed Western blots of Figure 1a (continue)

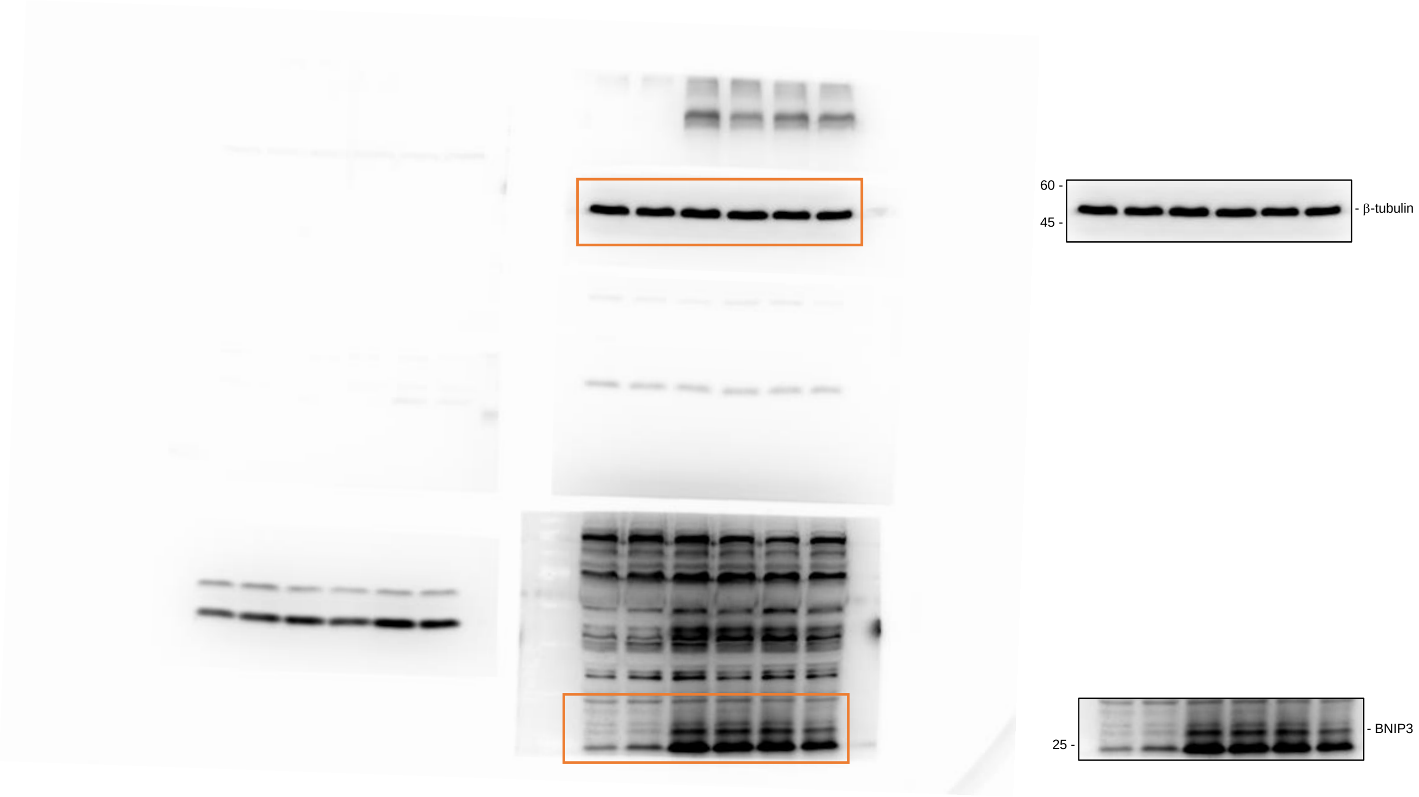

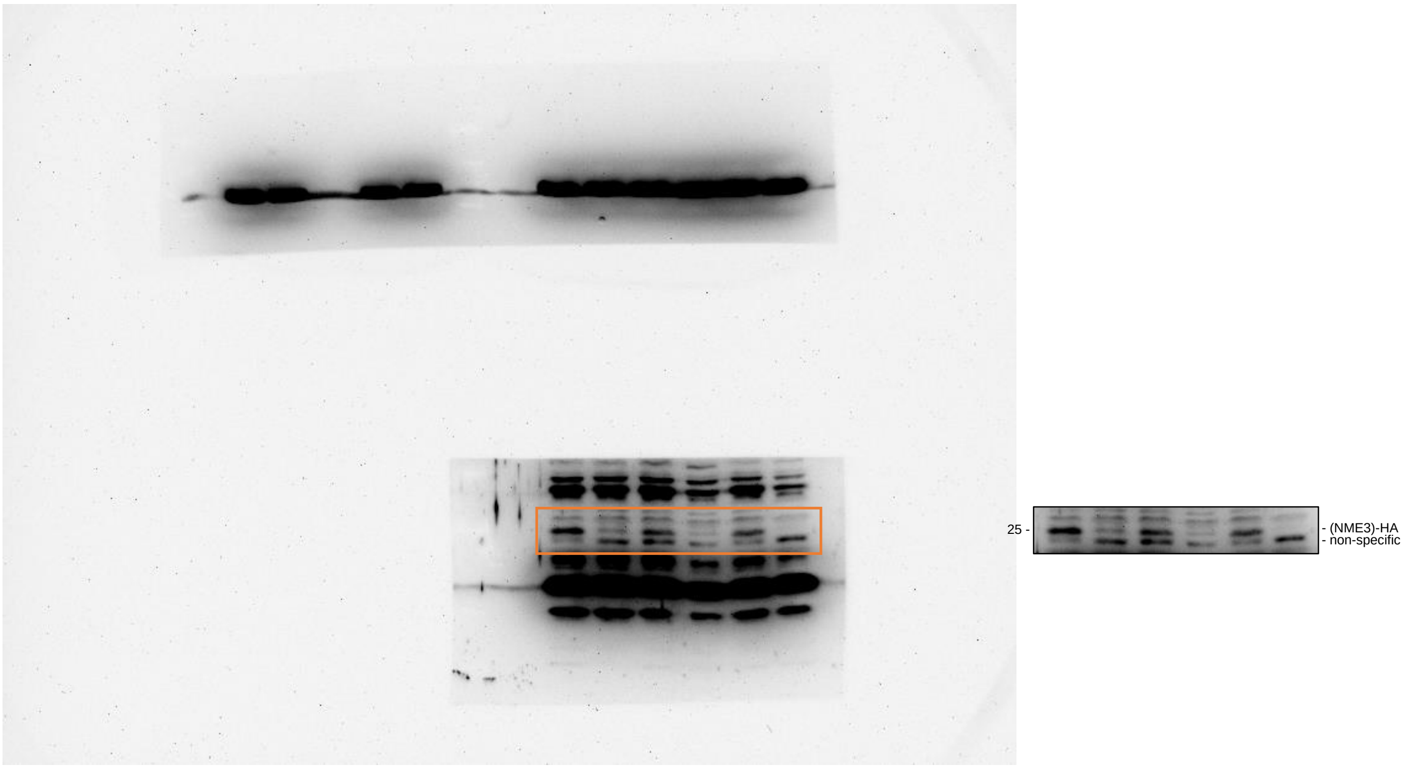

Unprocessed Western blots of Figure 1d

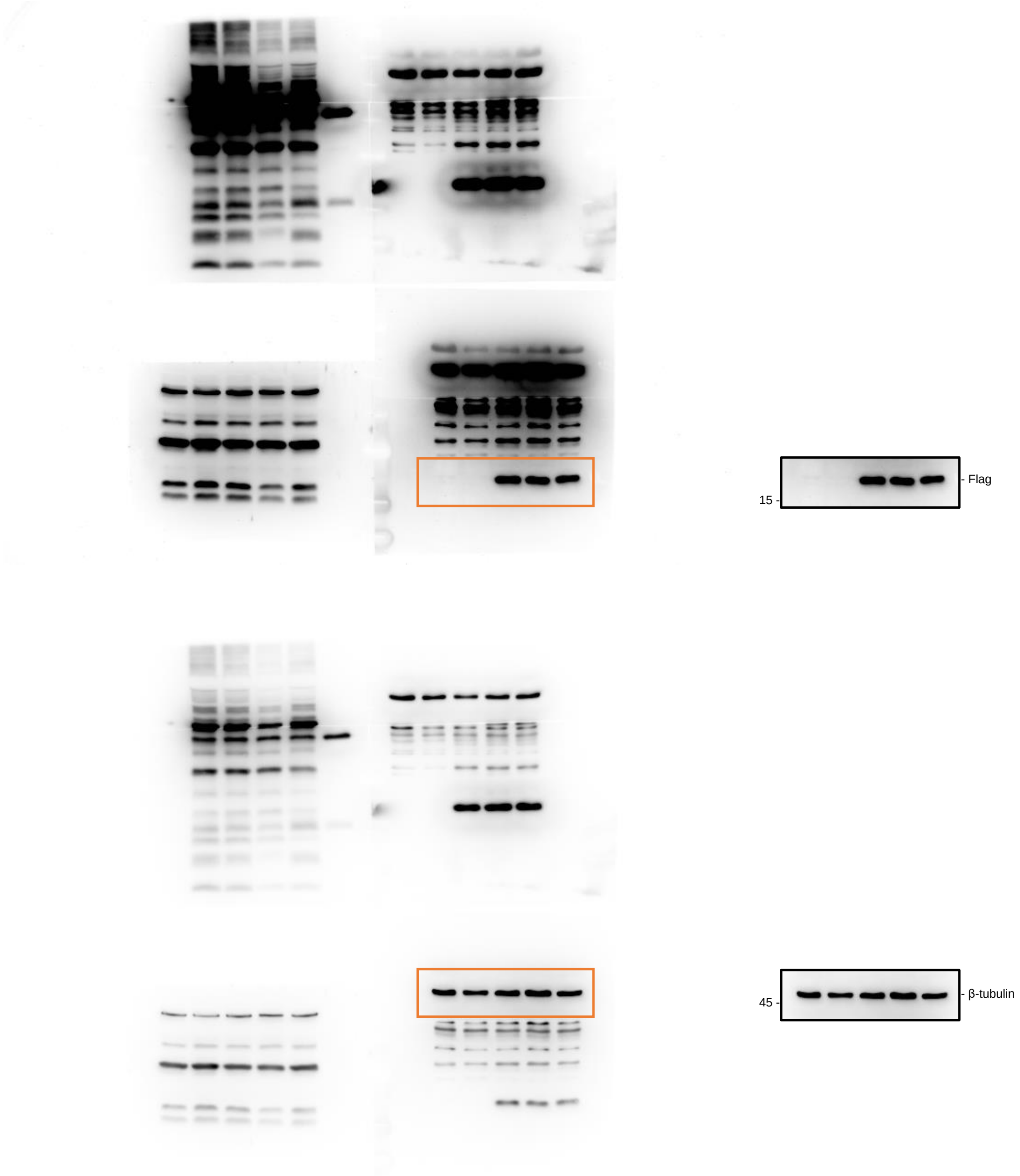

Unprocessed Western blots of Figure 1e

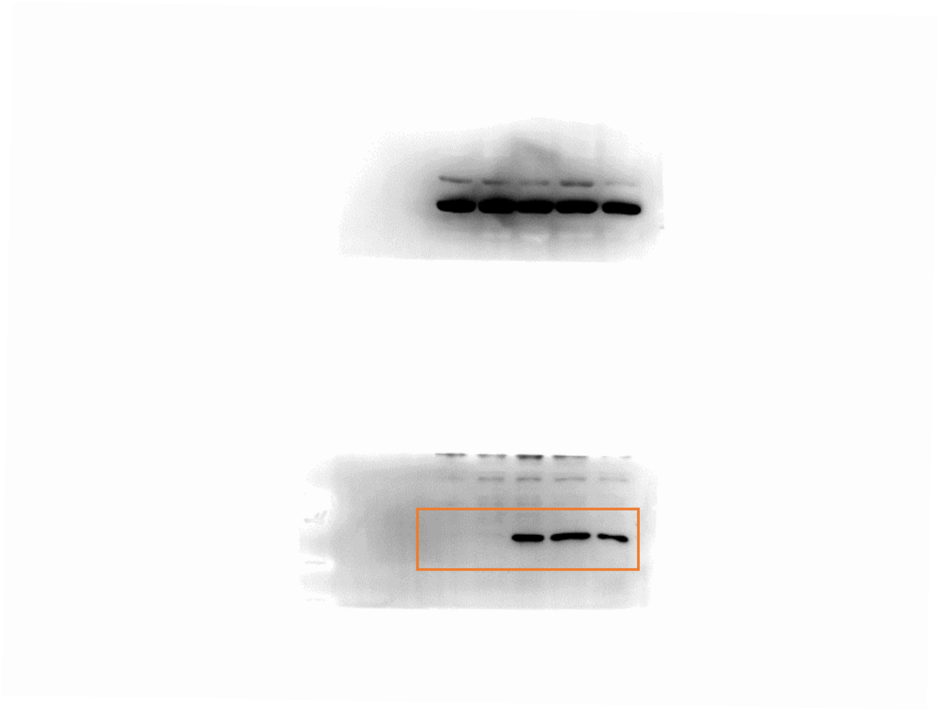

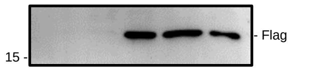

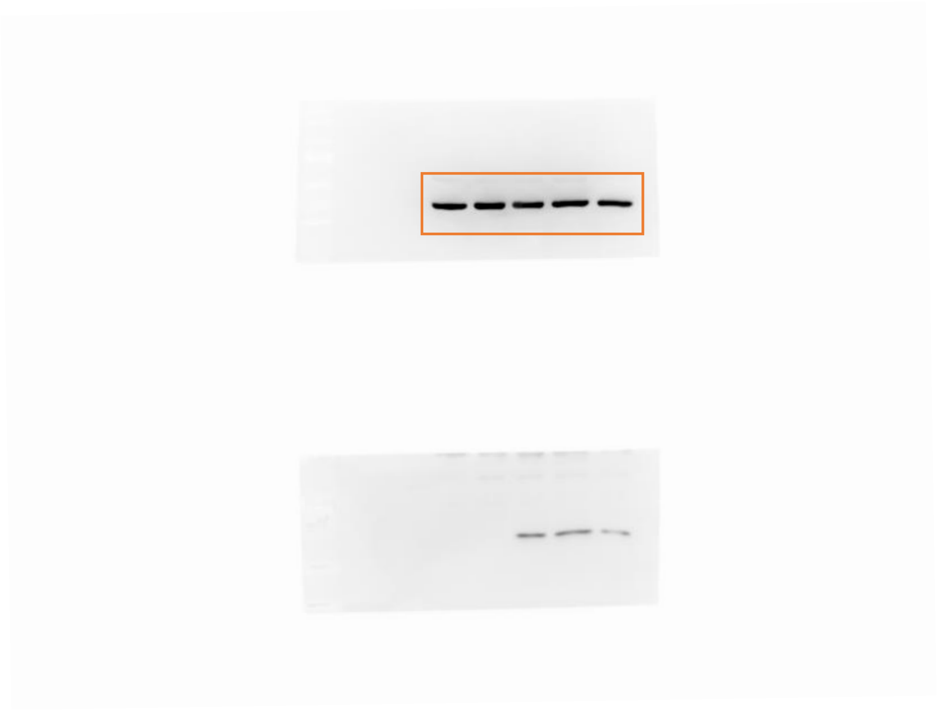

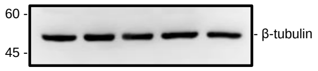

Unprocessed Western blots of Figure 1f

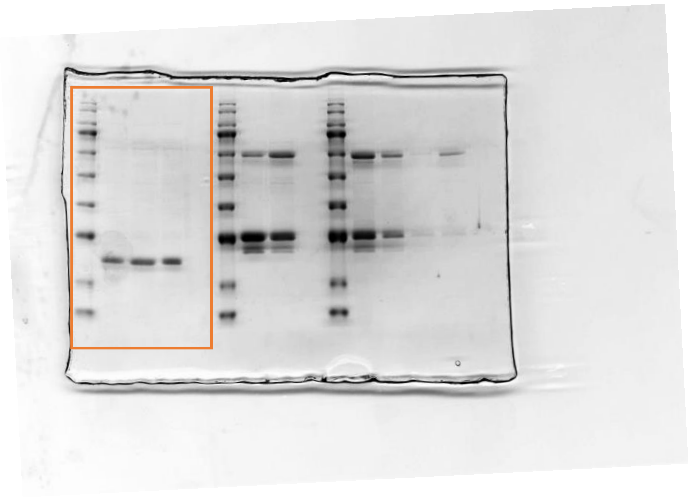

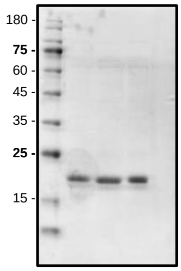

Unprocessed Western blots of Figure 1g

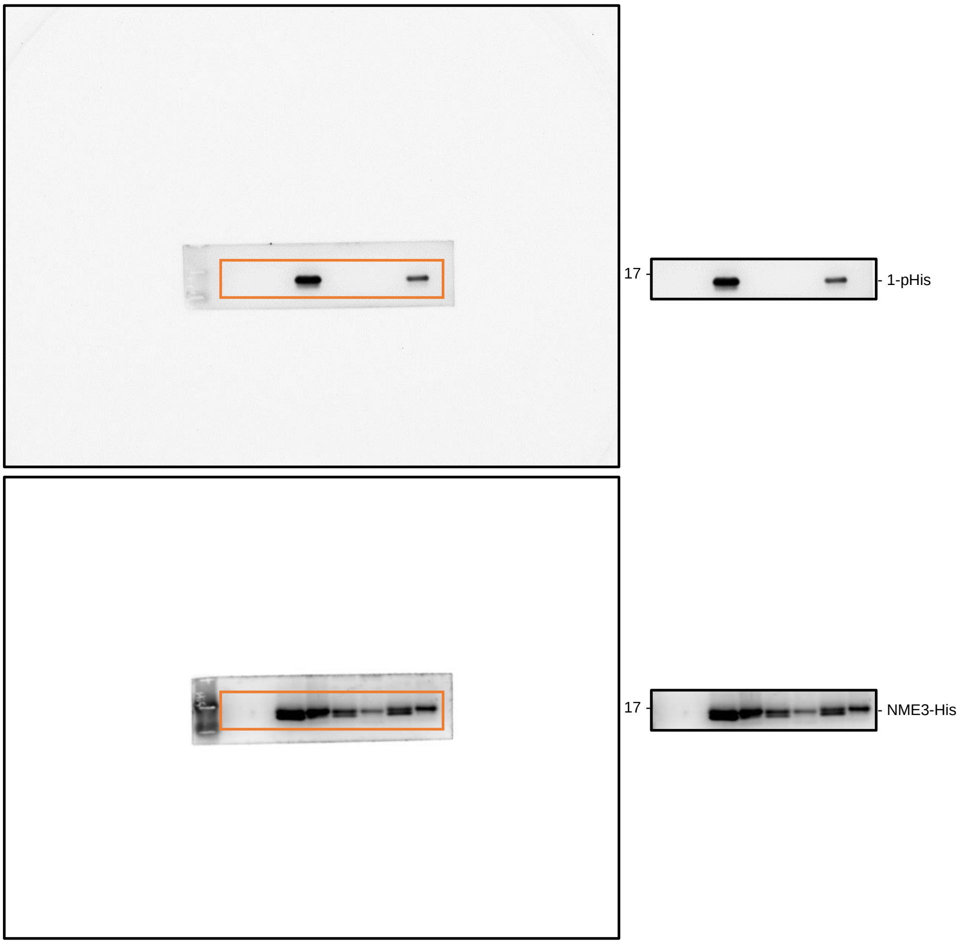

Unprocessed Western blots of Figure 2f

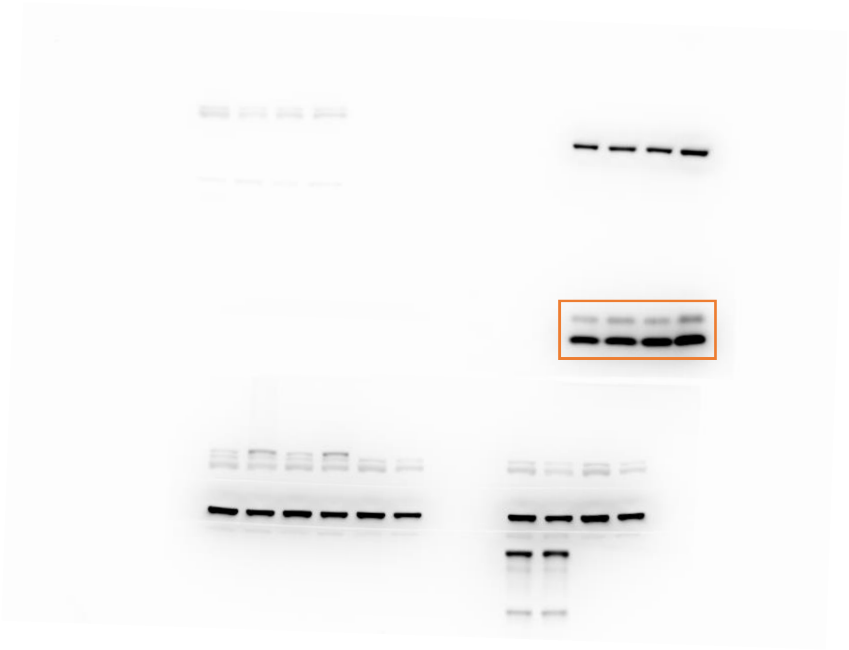

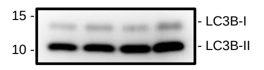

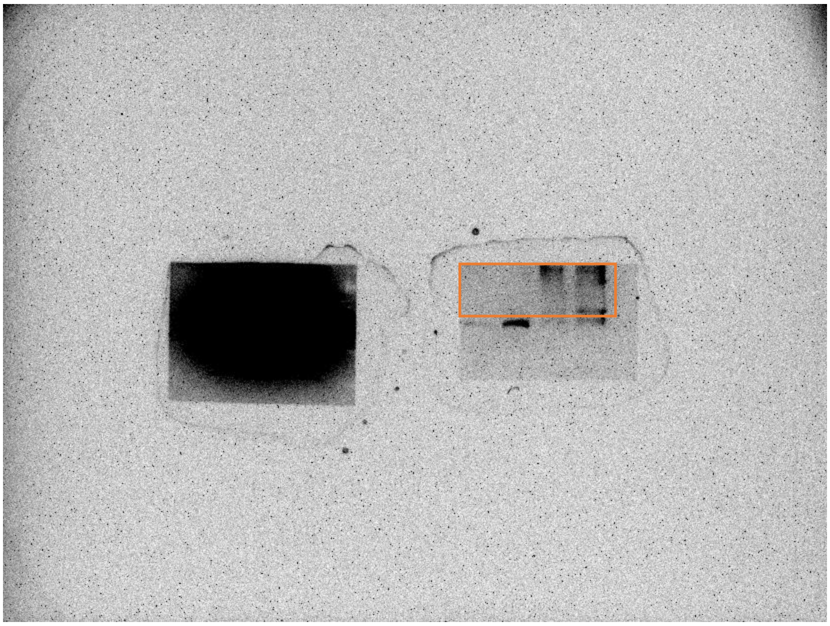

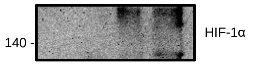

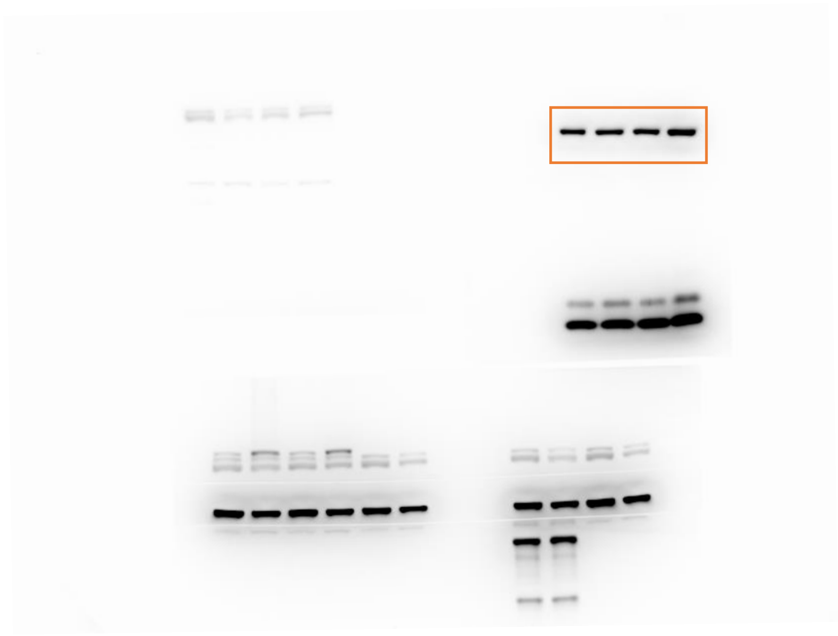

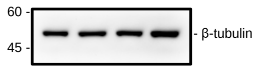

Unprocessed Western blots of Figure 2g

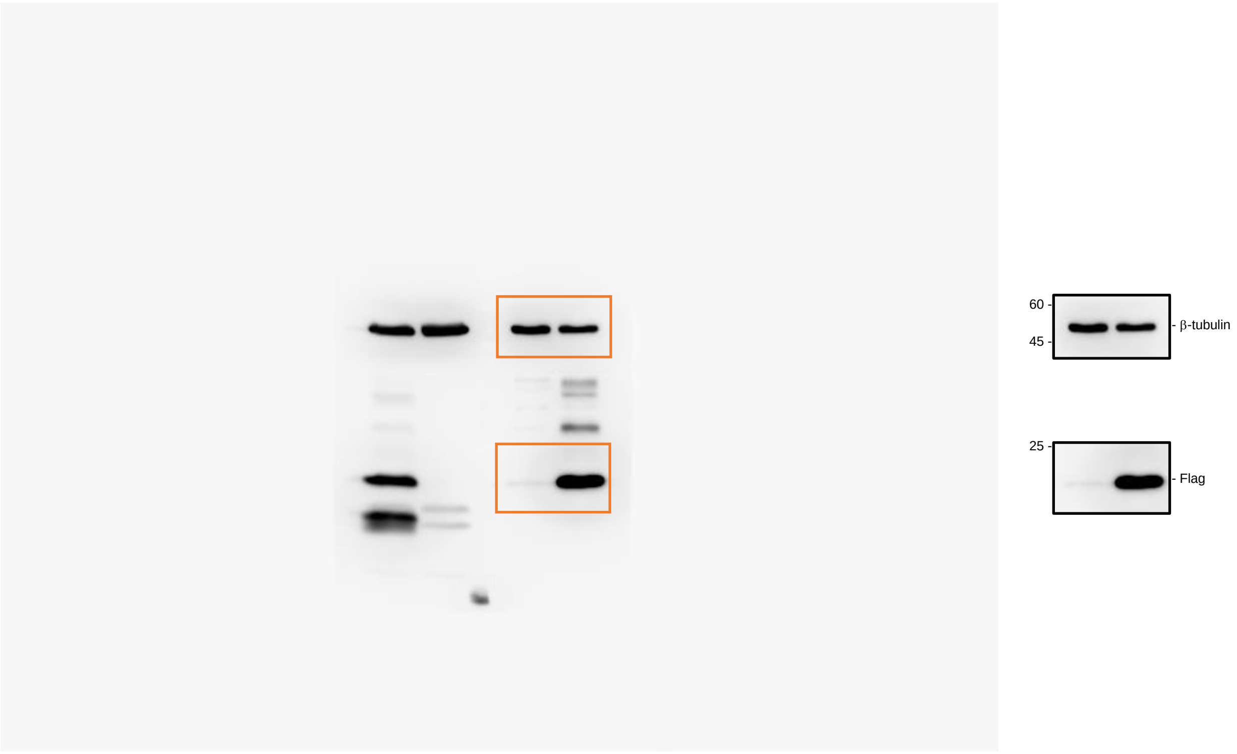

Unprocessed Western blots of Figure 3b

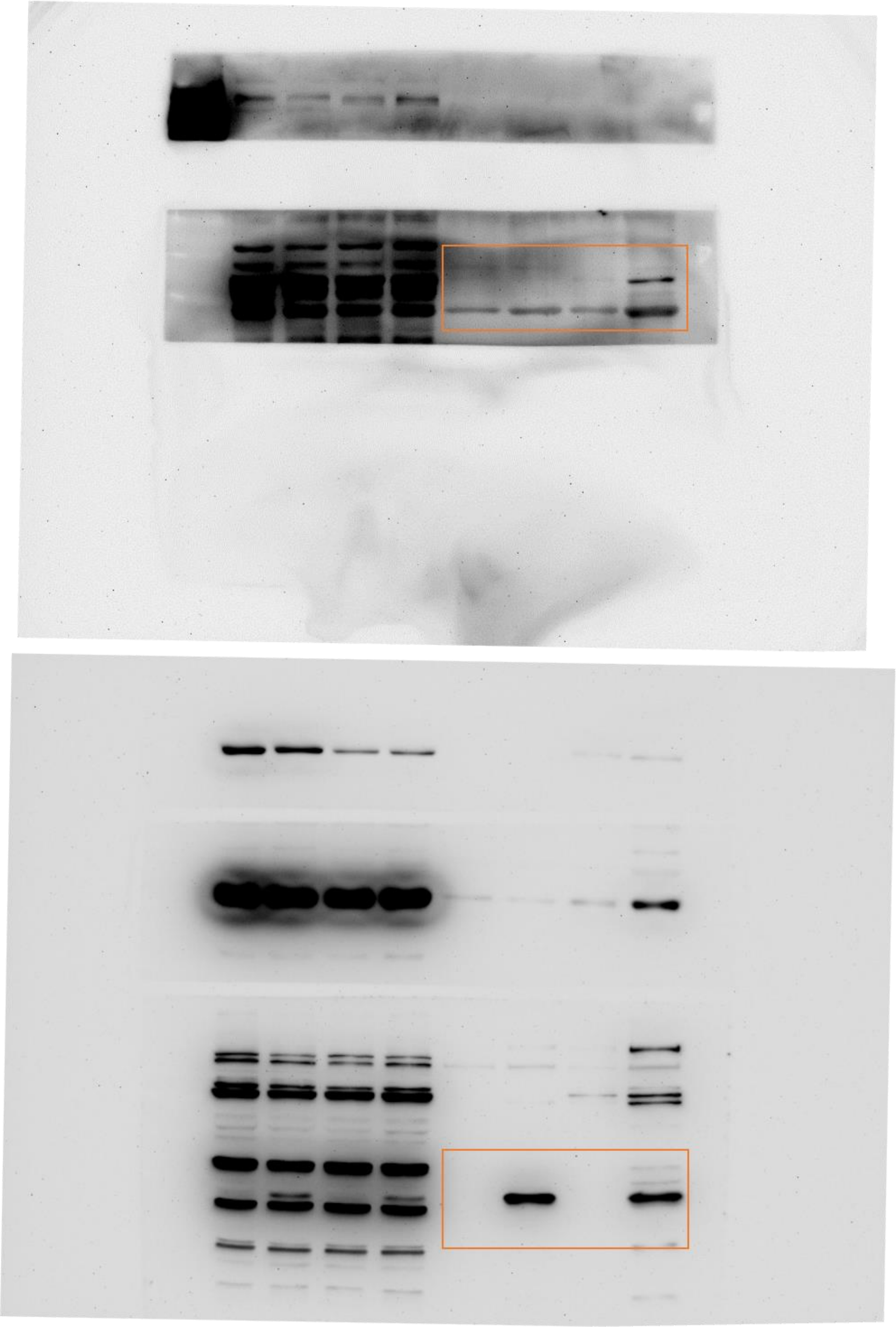

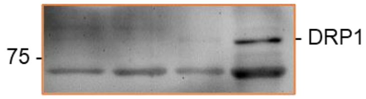

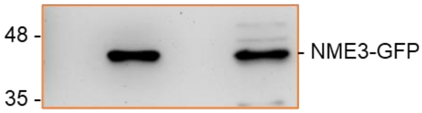

Unprocessed Western blots of Figure 3b (continue)

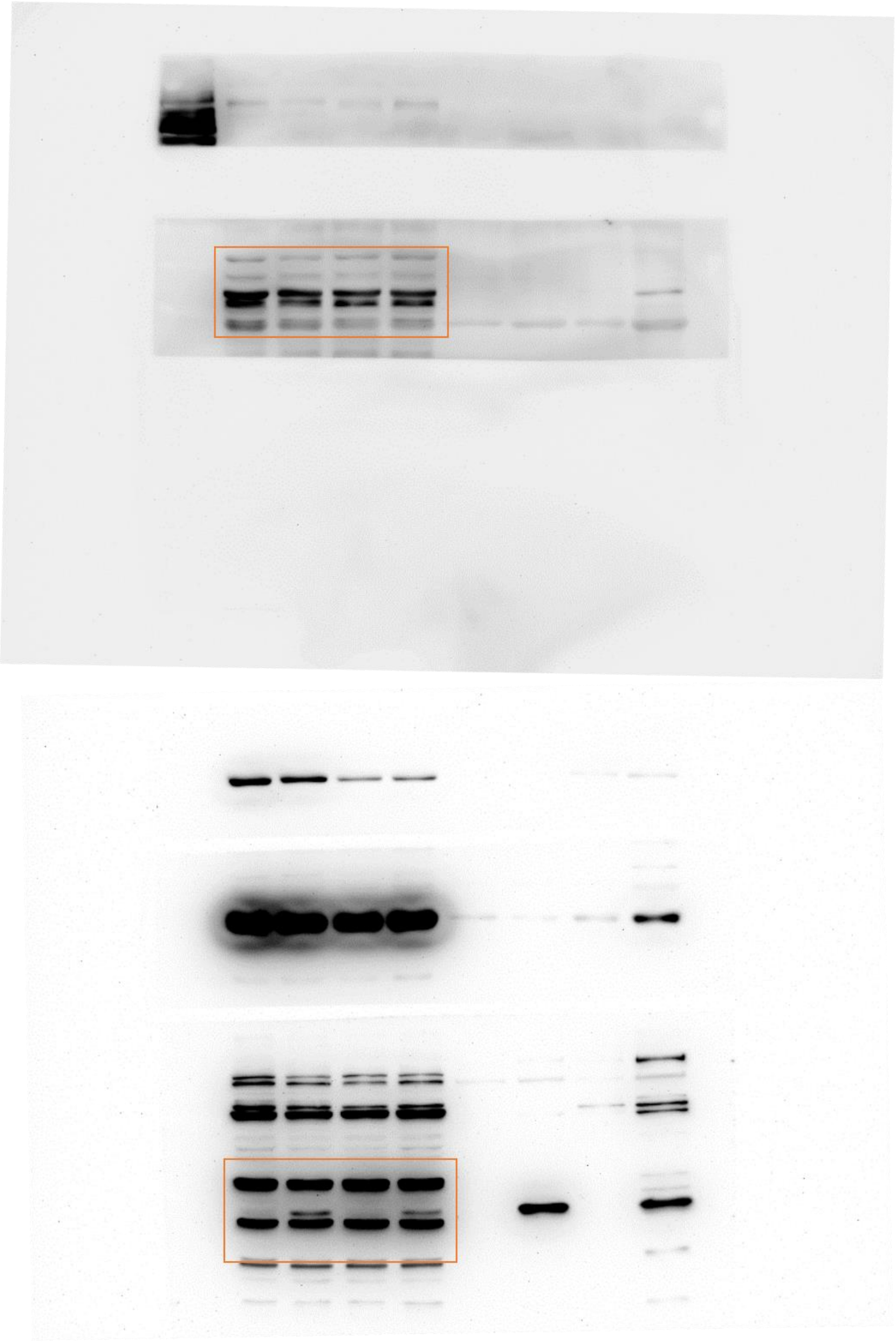

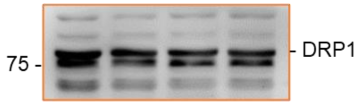

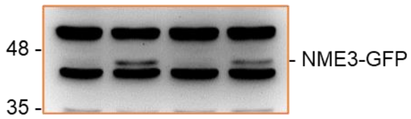

Unprocessed Western blots of Figure 3c

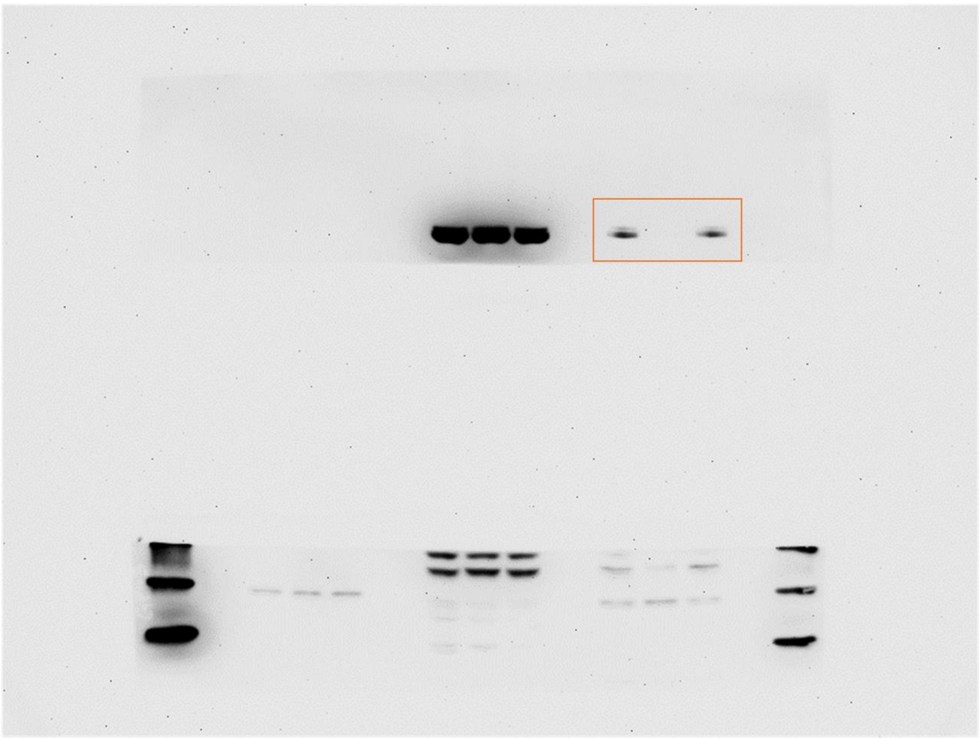

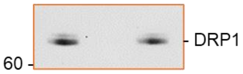

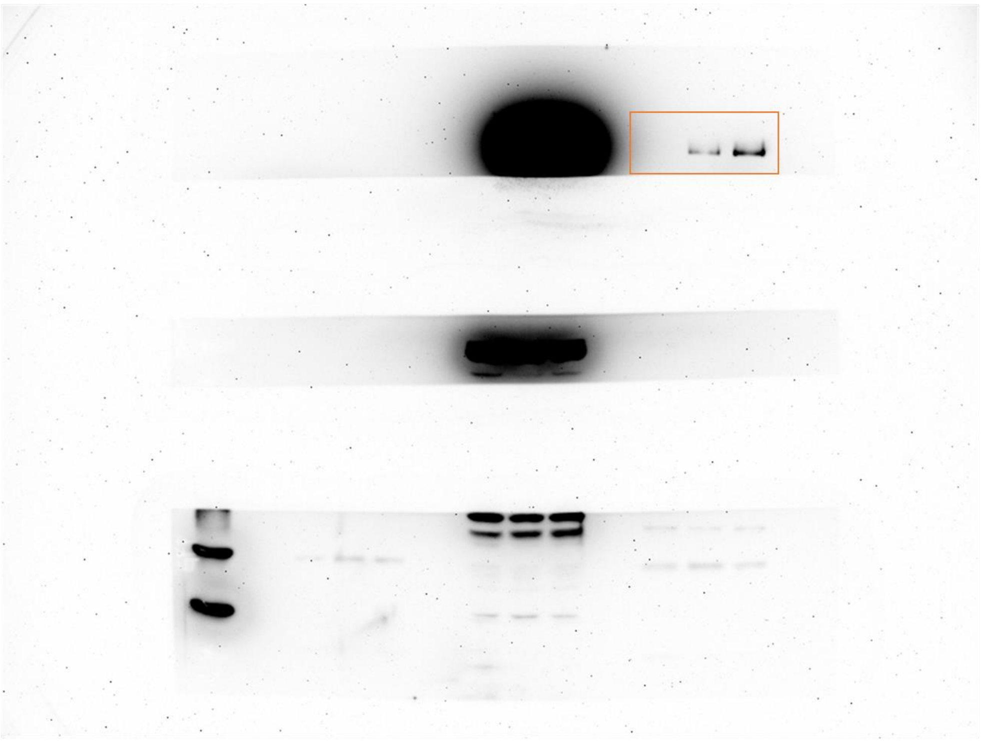

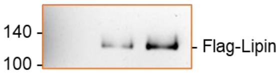

Unprocessed Western blots of Figure 3c

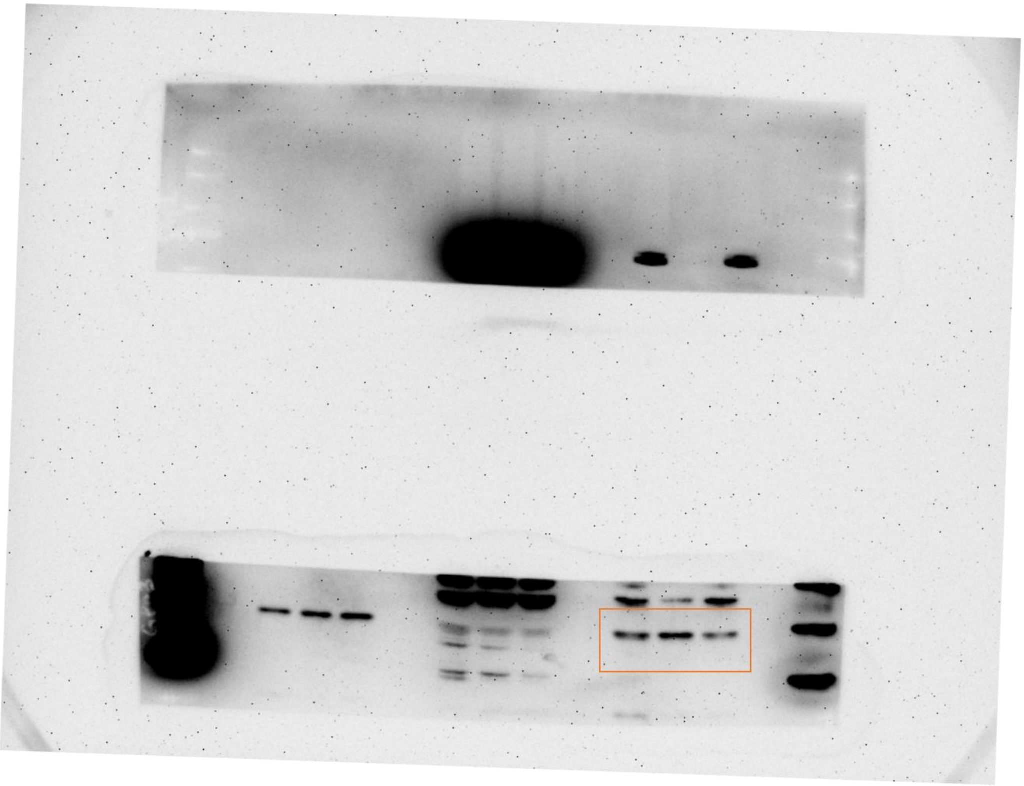

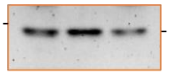

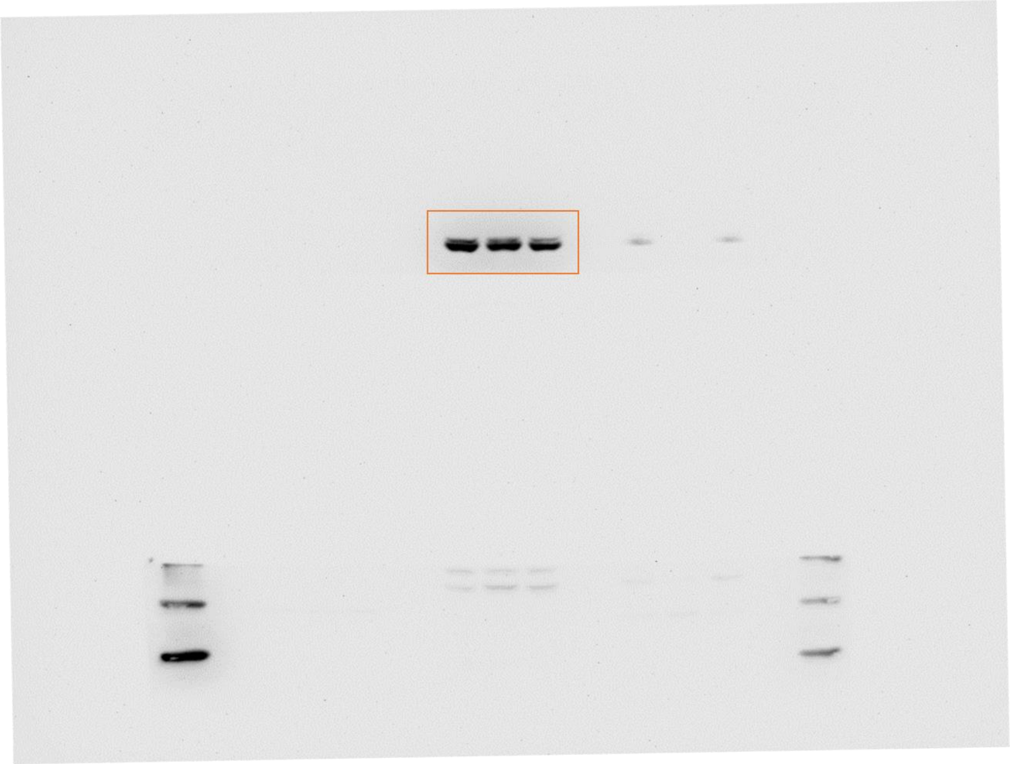

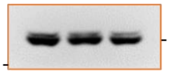

Unprocessed Western blots of Figure 3c

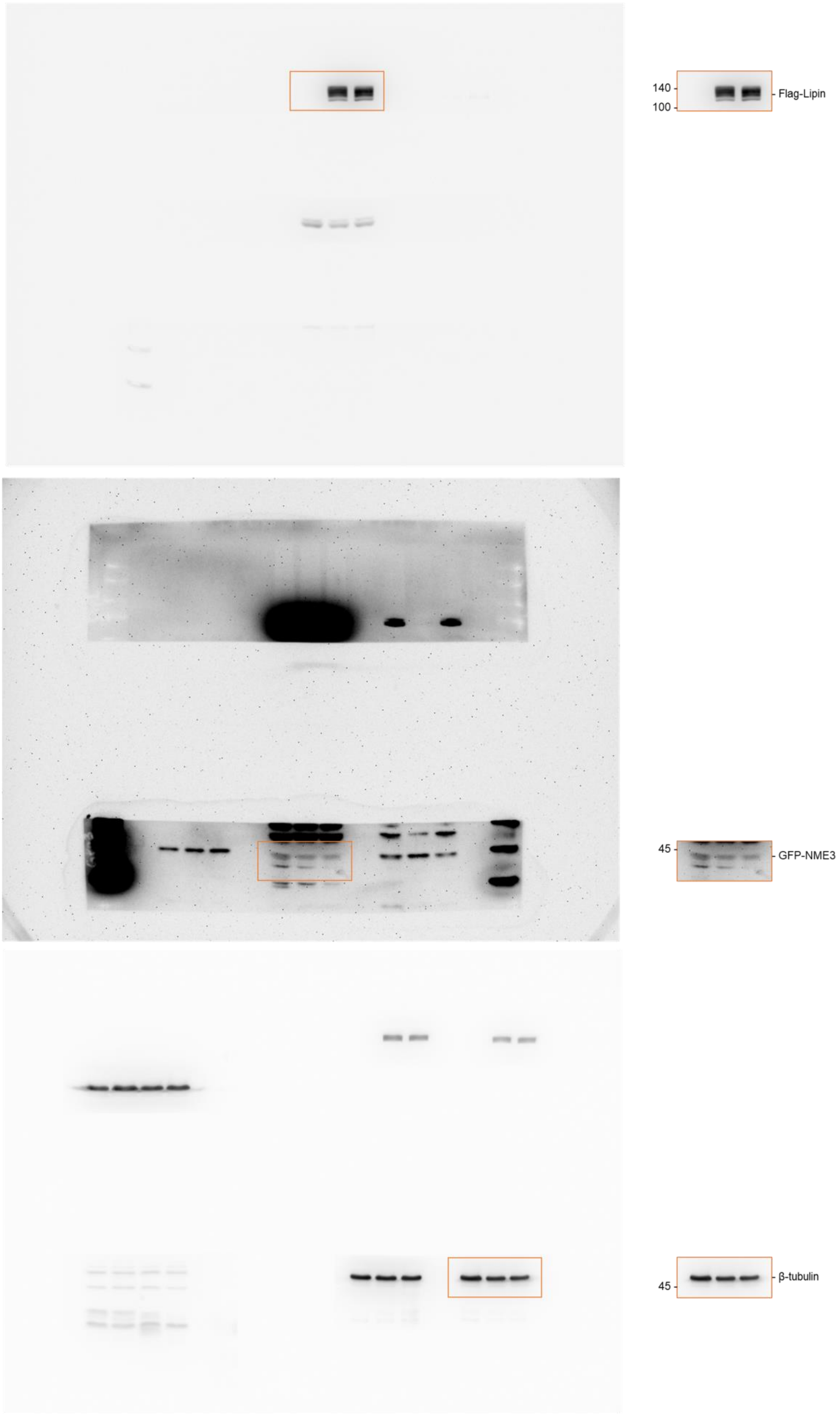

Unprocessed Western blots of Figure 3f

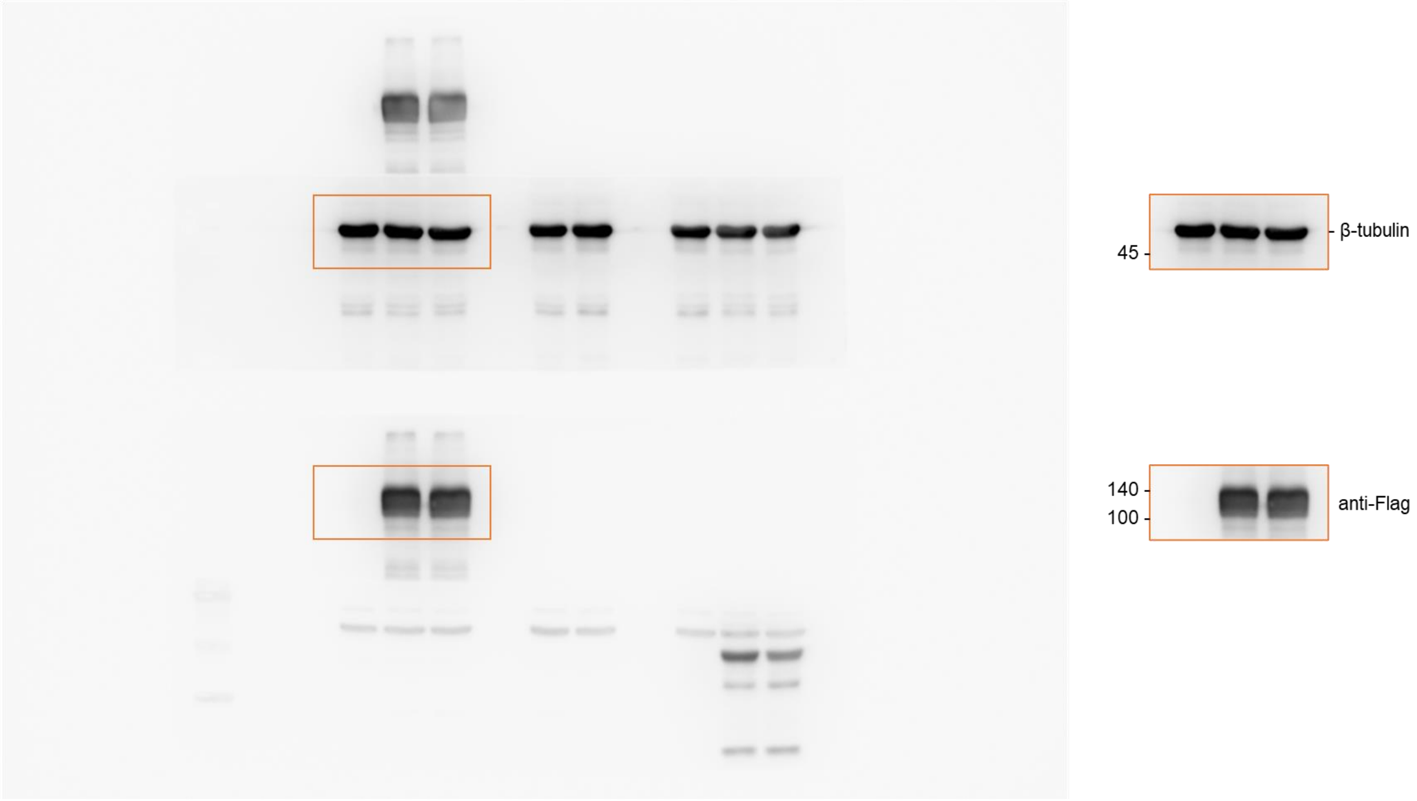

Unprocessed Western blots of Figure 3g

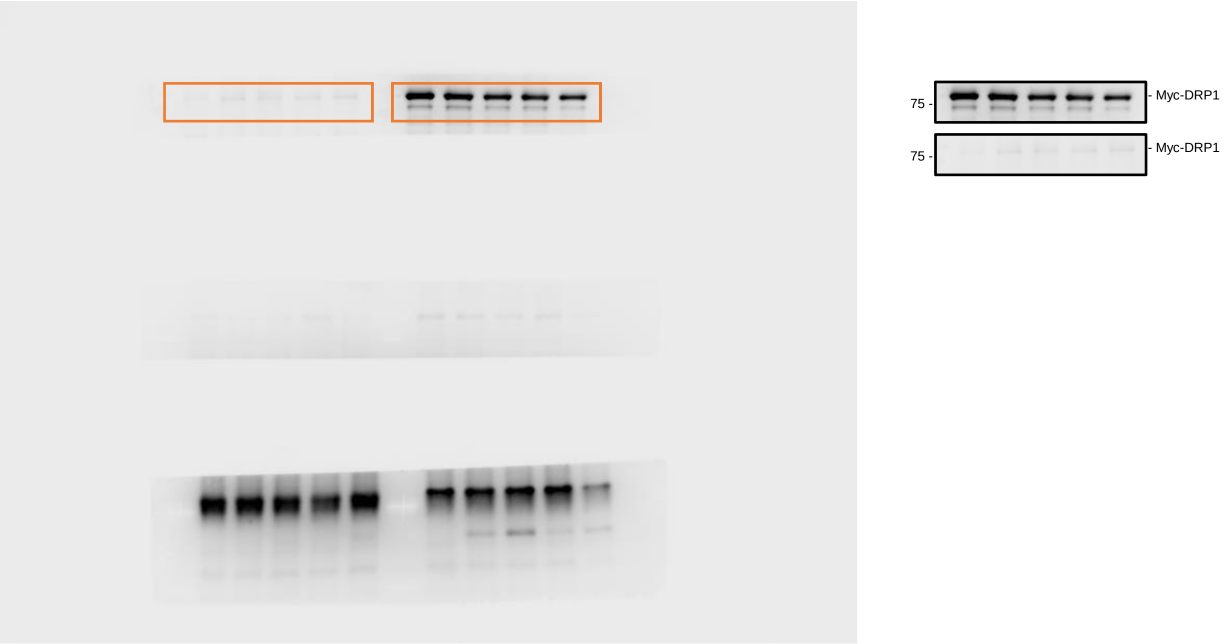

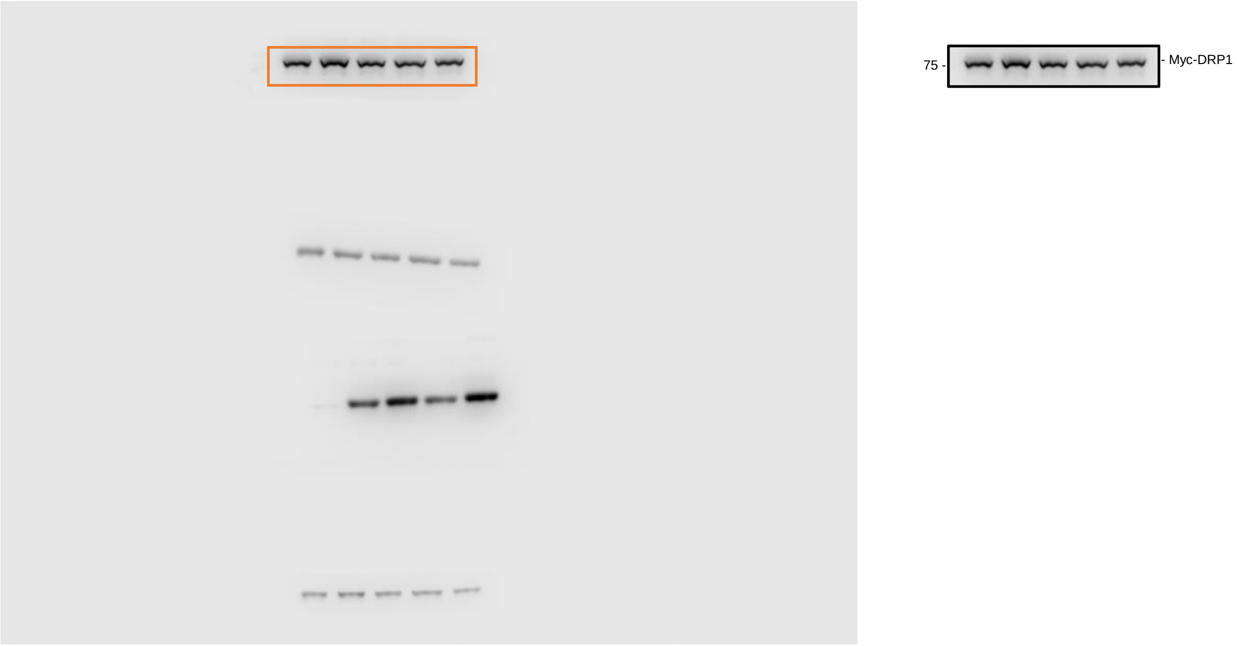

Unprocessed Western blots of Figure 3g (continue)

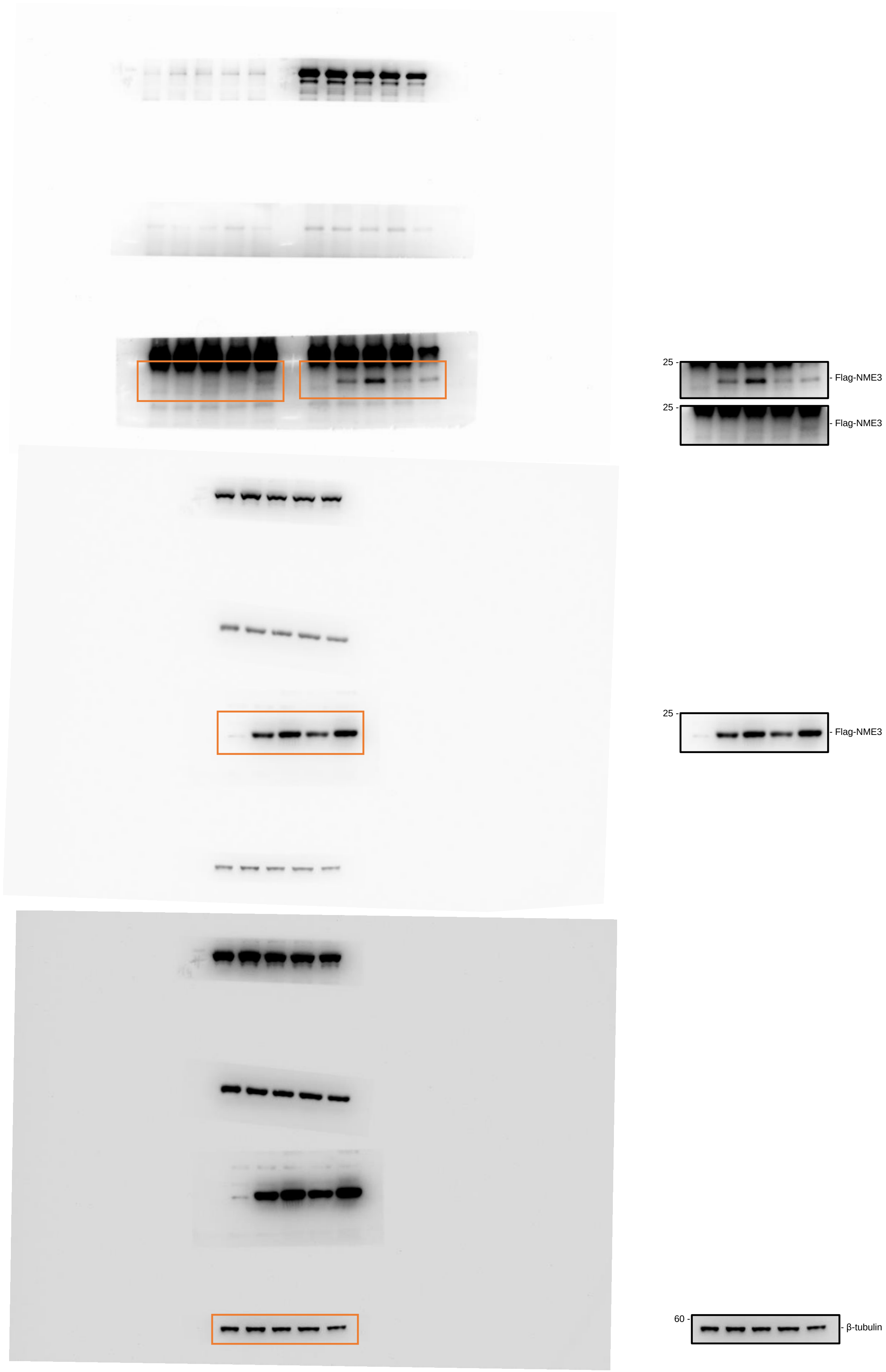

Unprocessed Western blots of Figure 3h

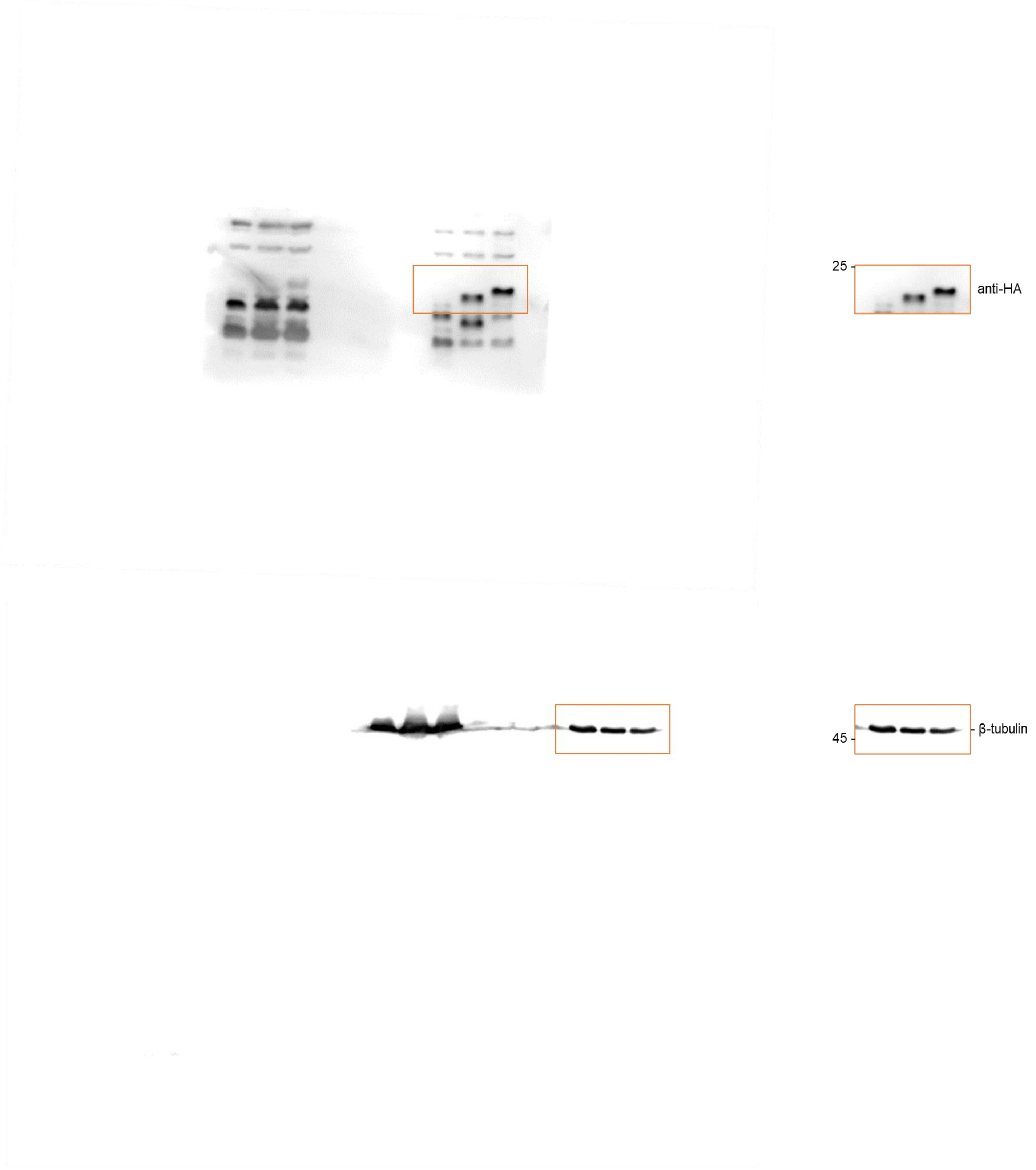

Unprocessed Western blots of Figure 4b

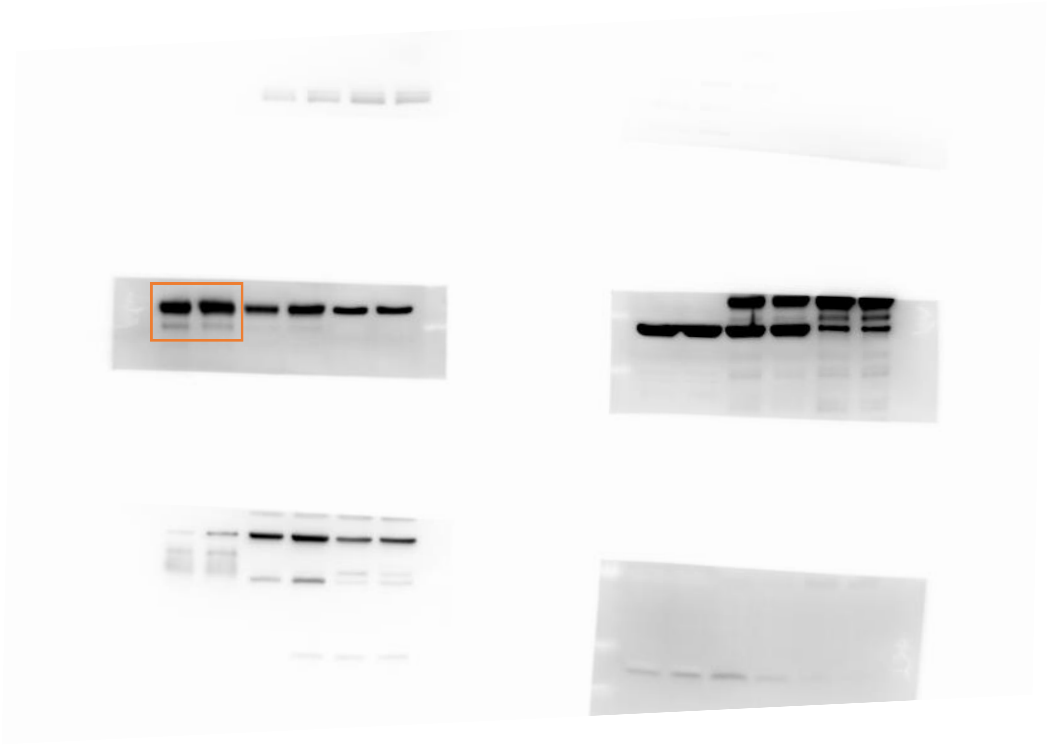

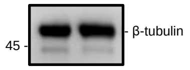

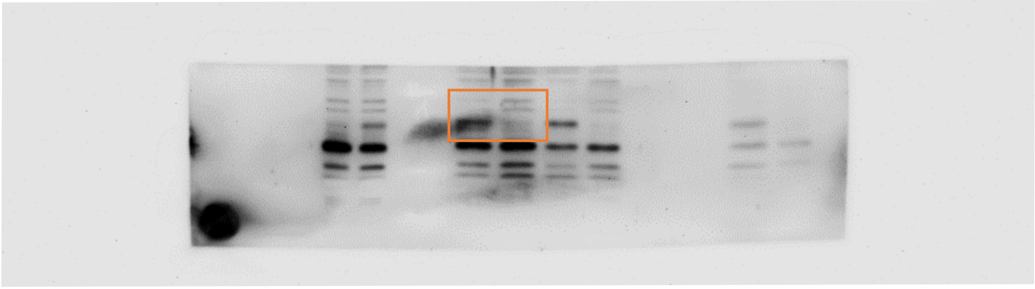

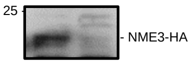

Unprocessed Western blots of Figure 4e

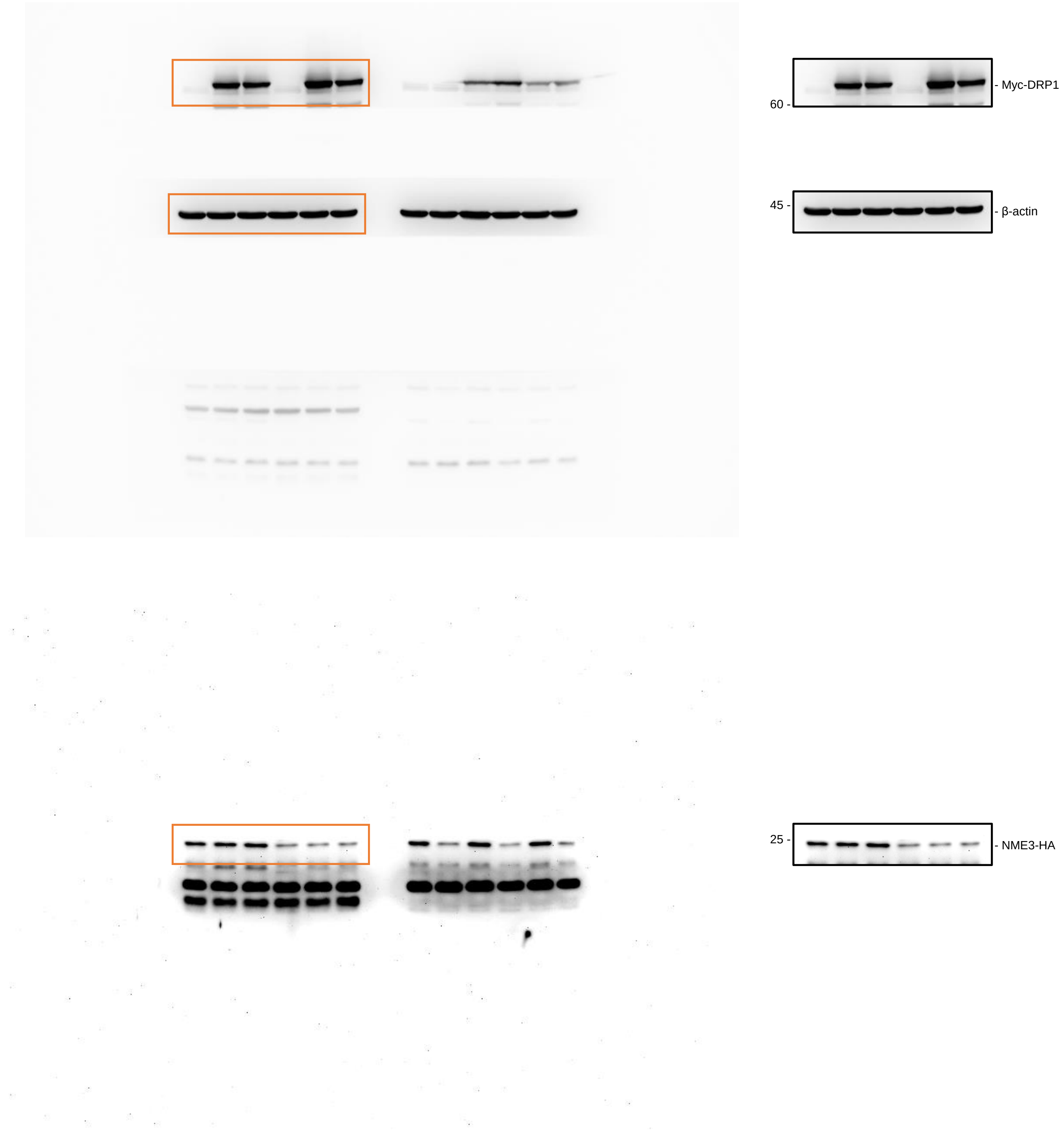

Unprocessed Western blots of Figure 4g

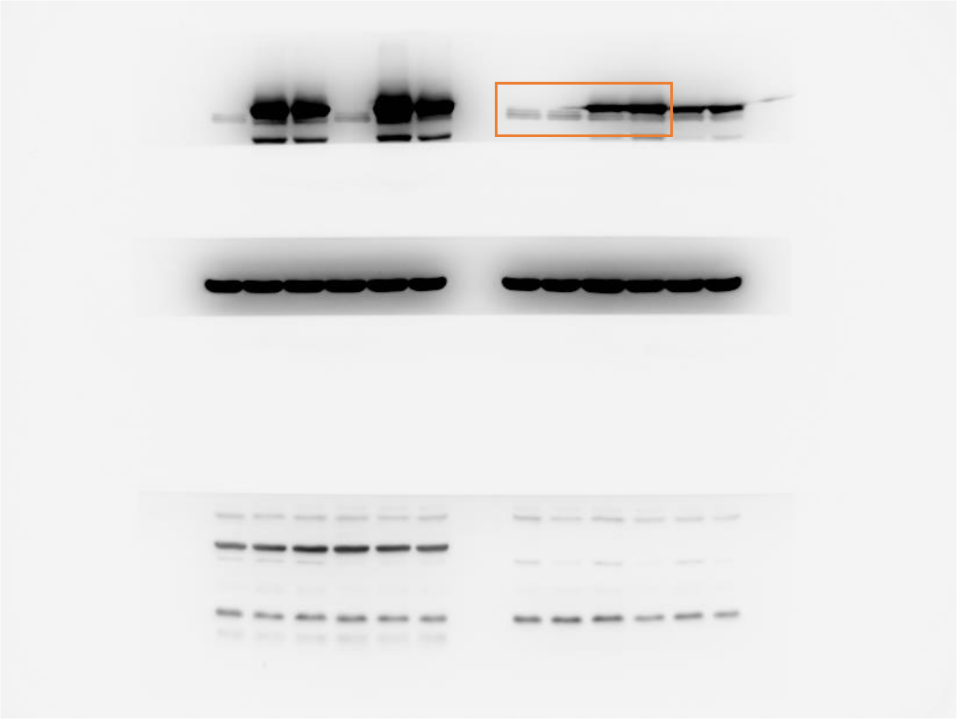

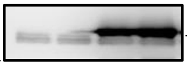

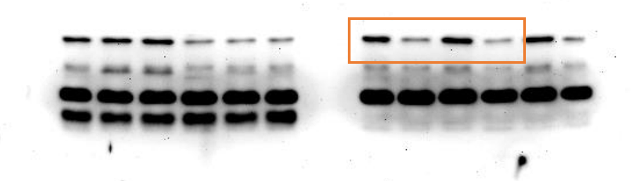

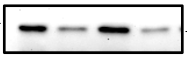

Unprocessed Western blots of Figure 4g (continue)

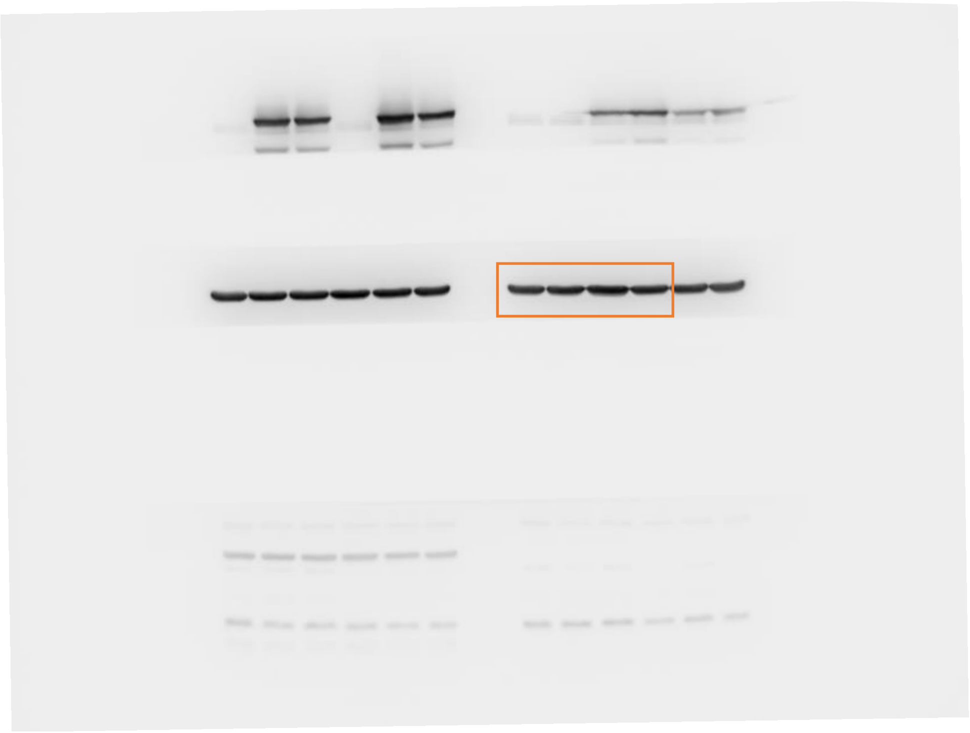

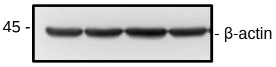

Unprocessed western blots of Figure 4h

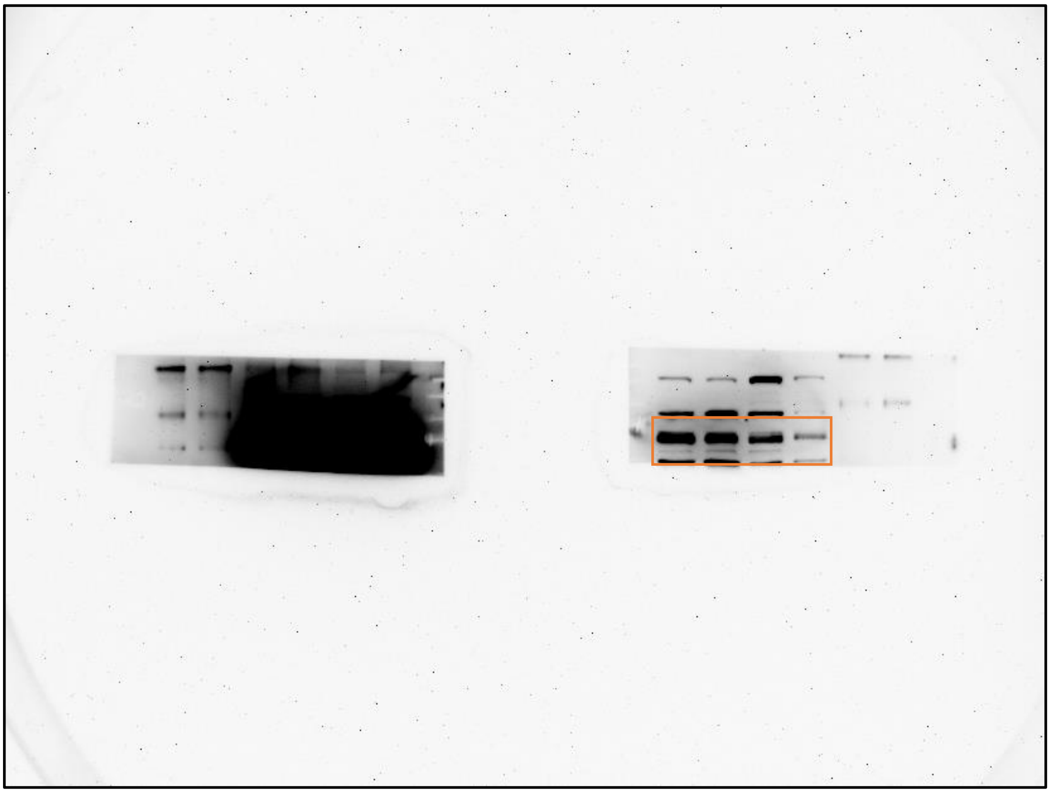

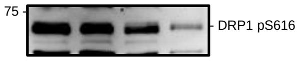

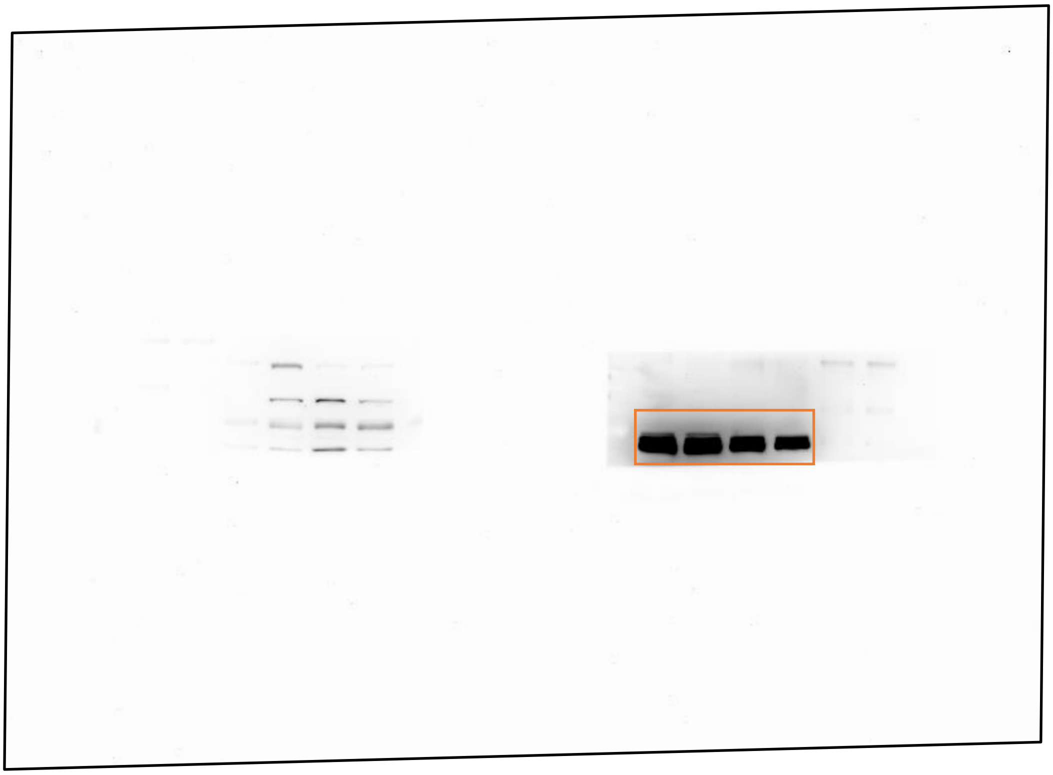

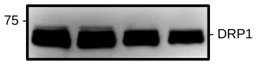

Unprocessed western blots of Figure 4h (continue)

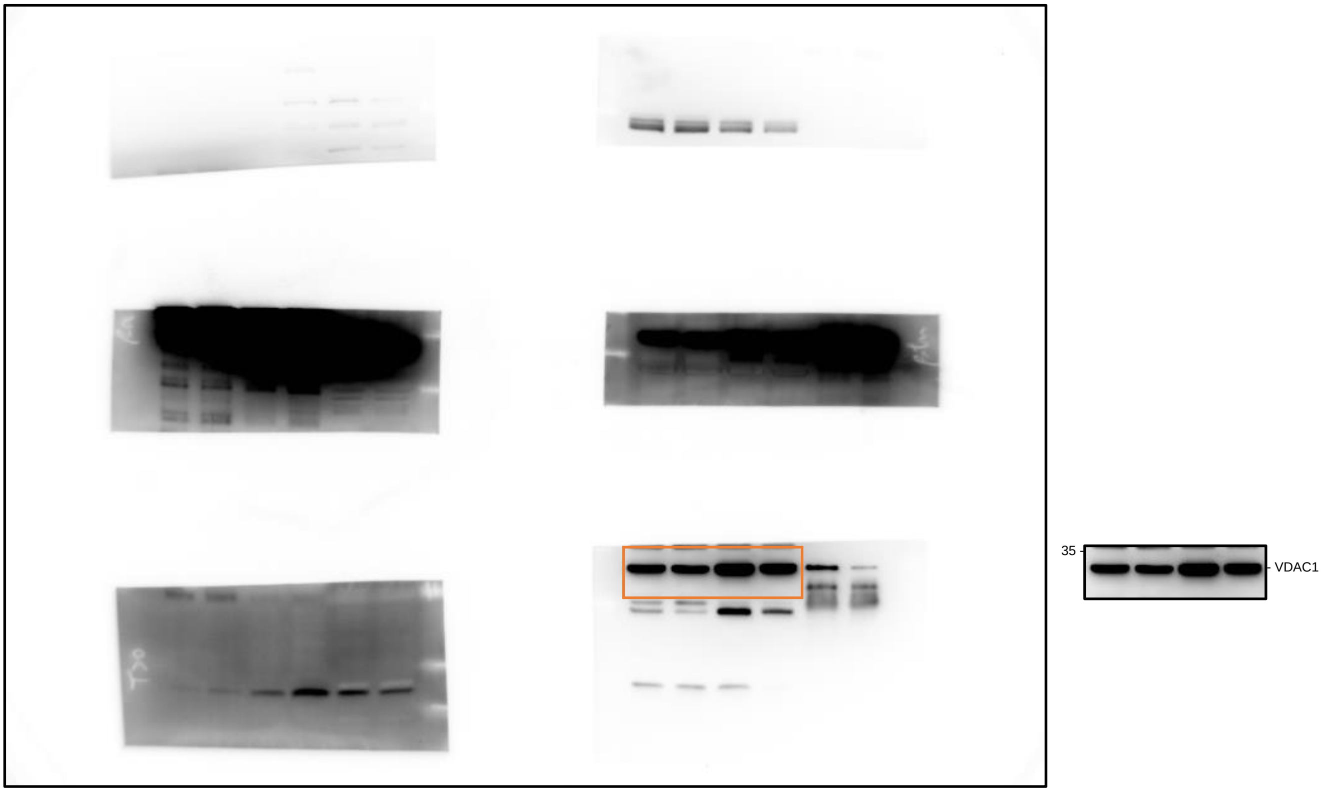

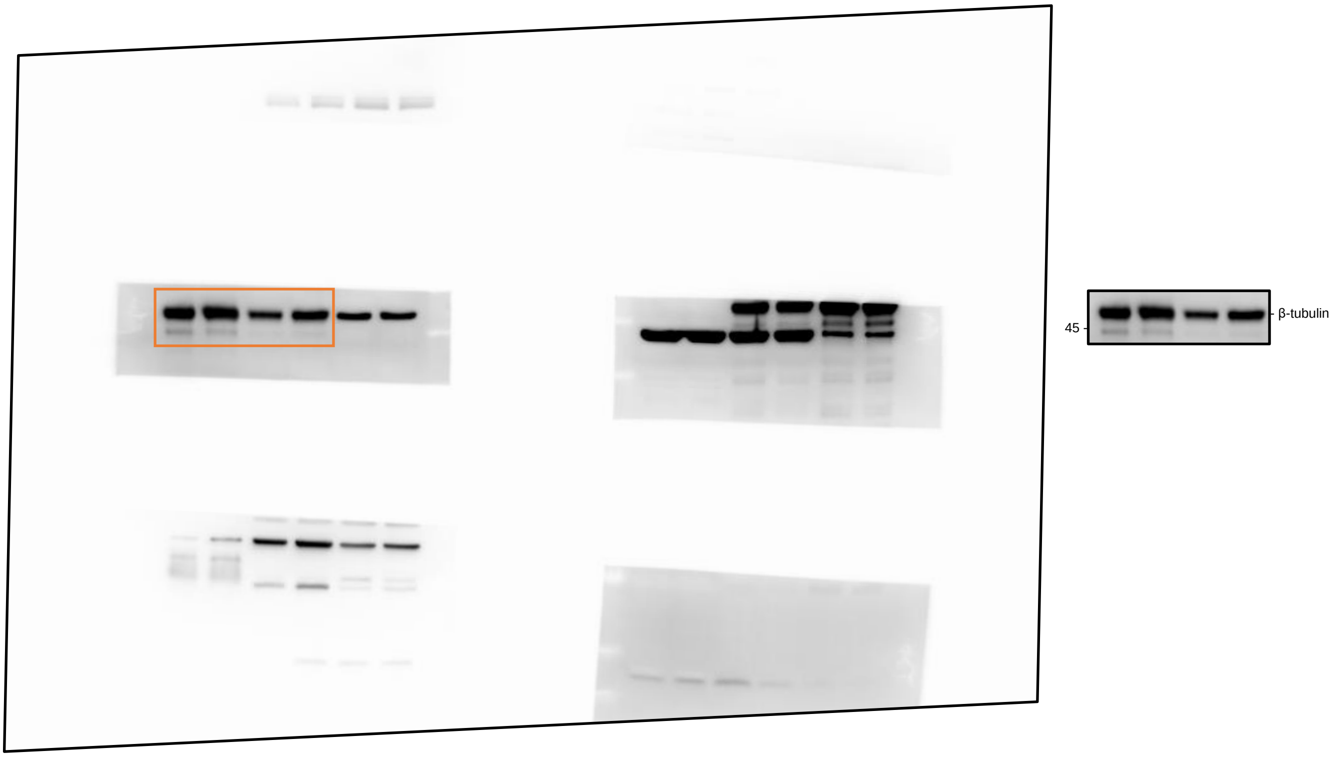

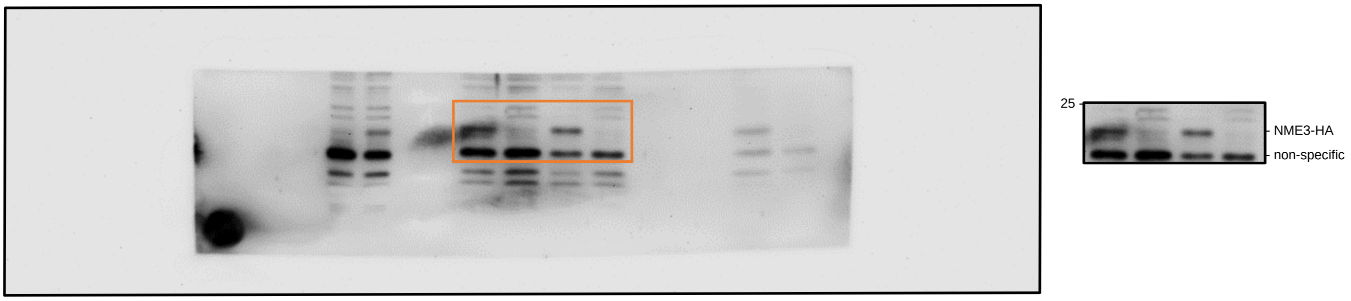

Unprocessed Western blots of Figure 5a

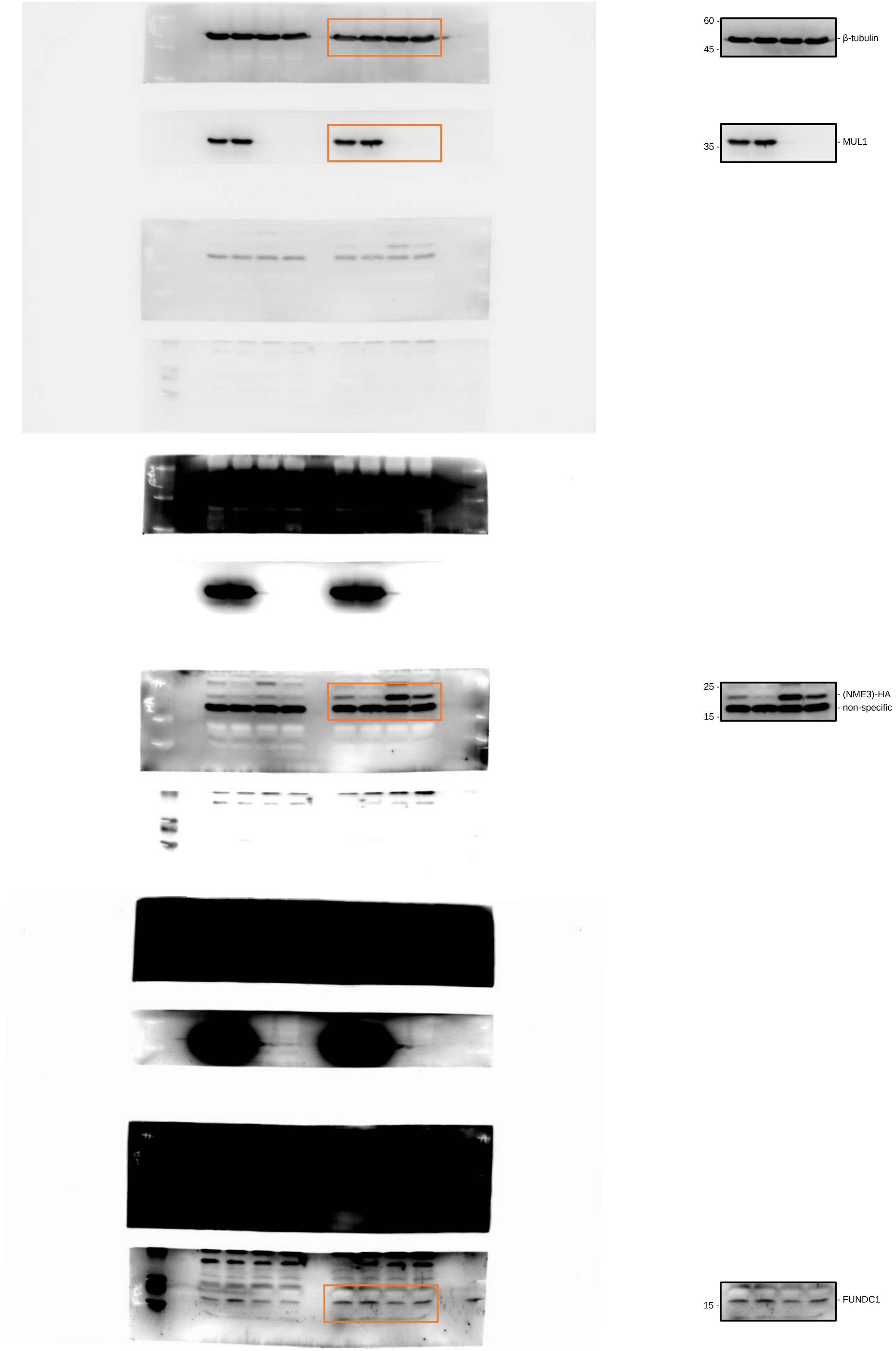

Unprocessed Western blots of Figure 5b

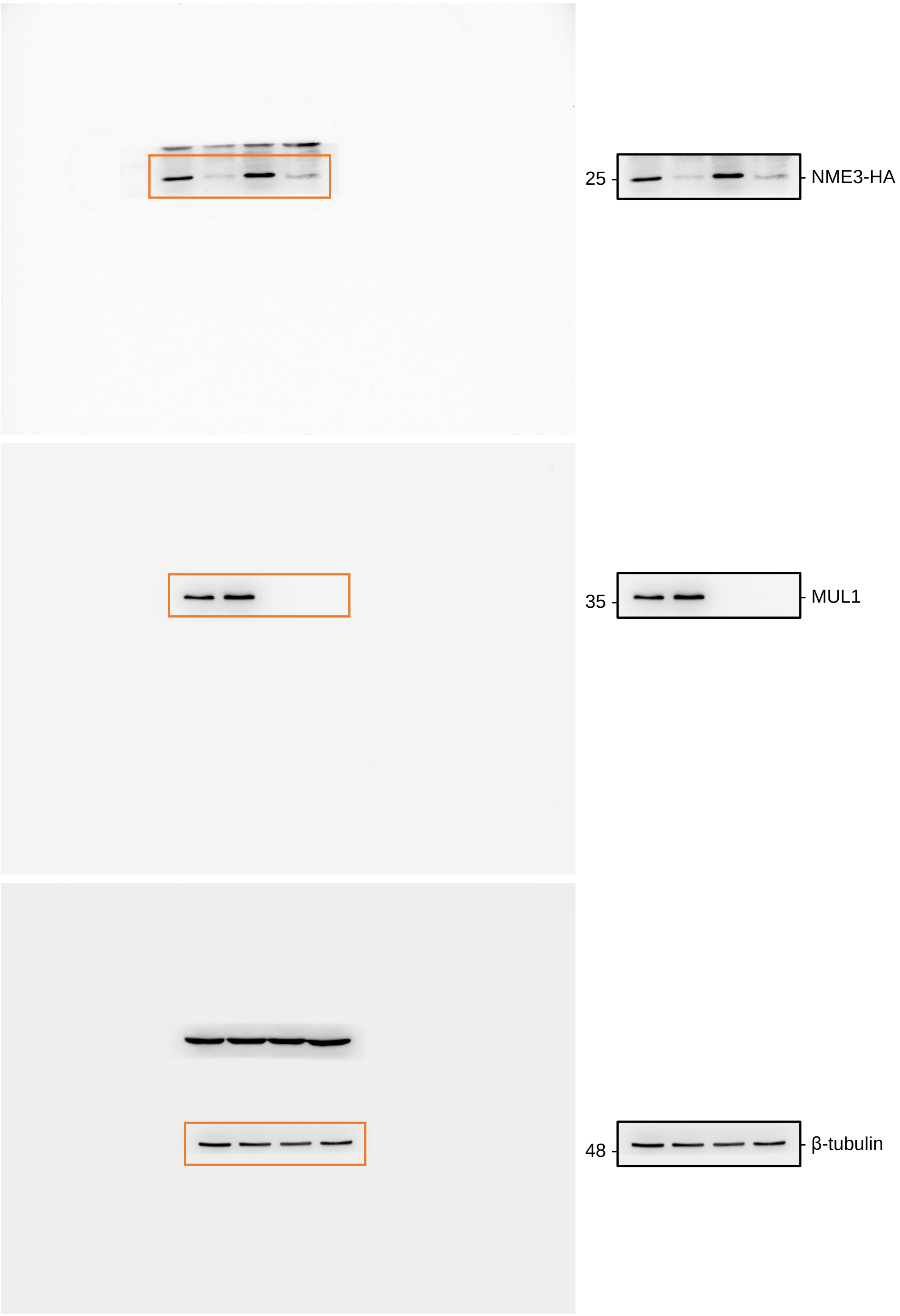

Unprocessed Western blots of Figure 5d

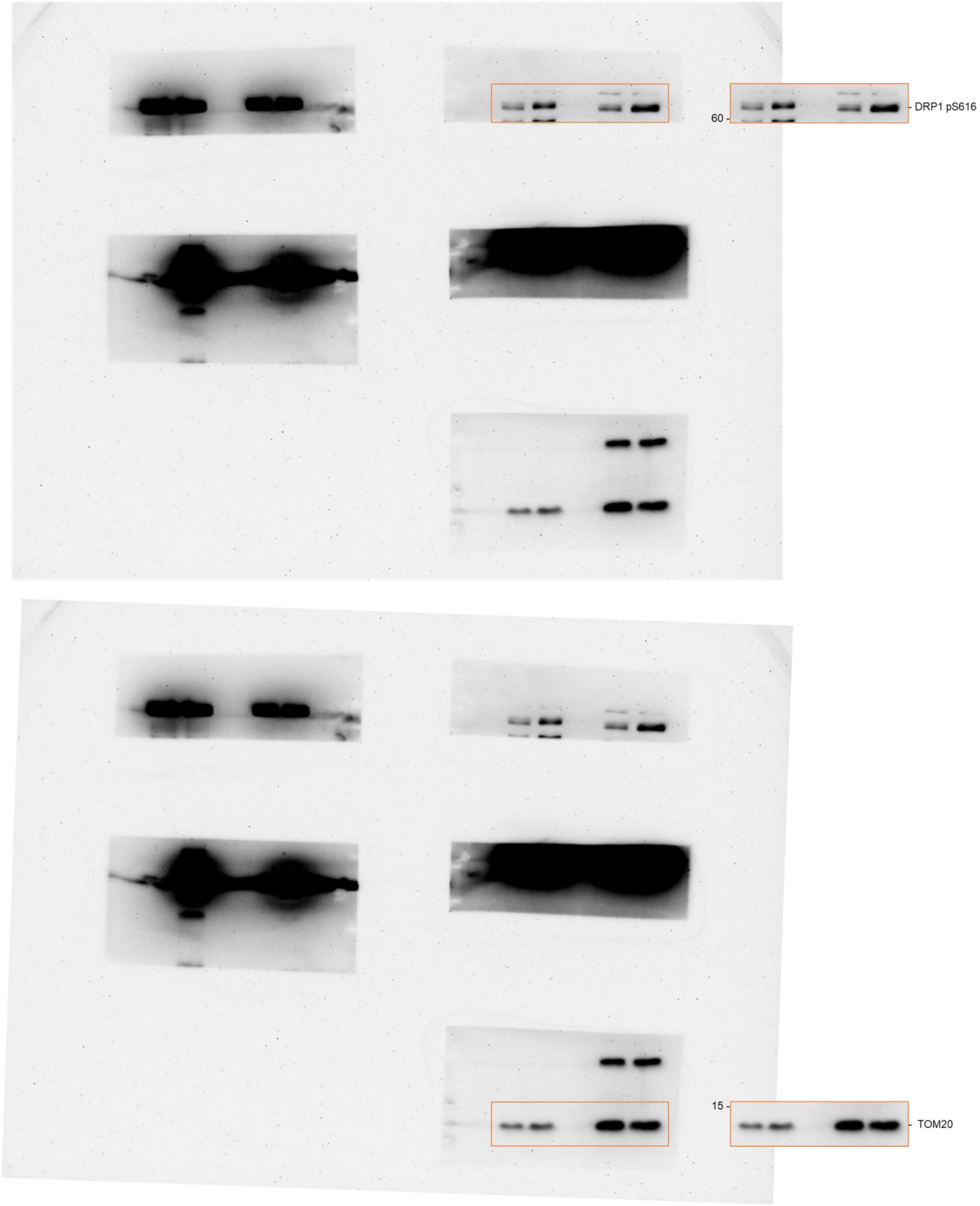

Unprocessed Western blots of Figure 5d (continue)

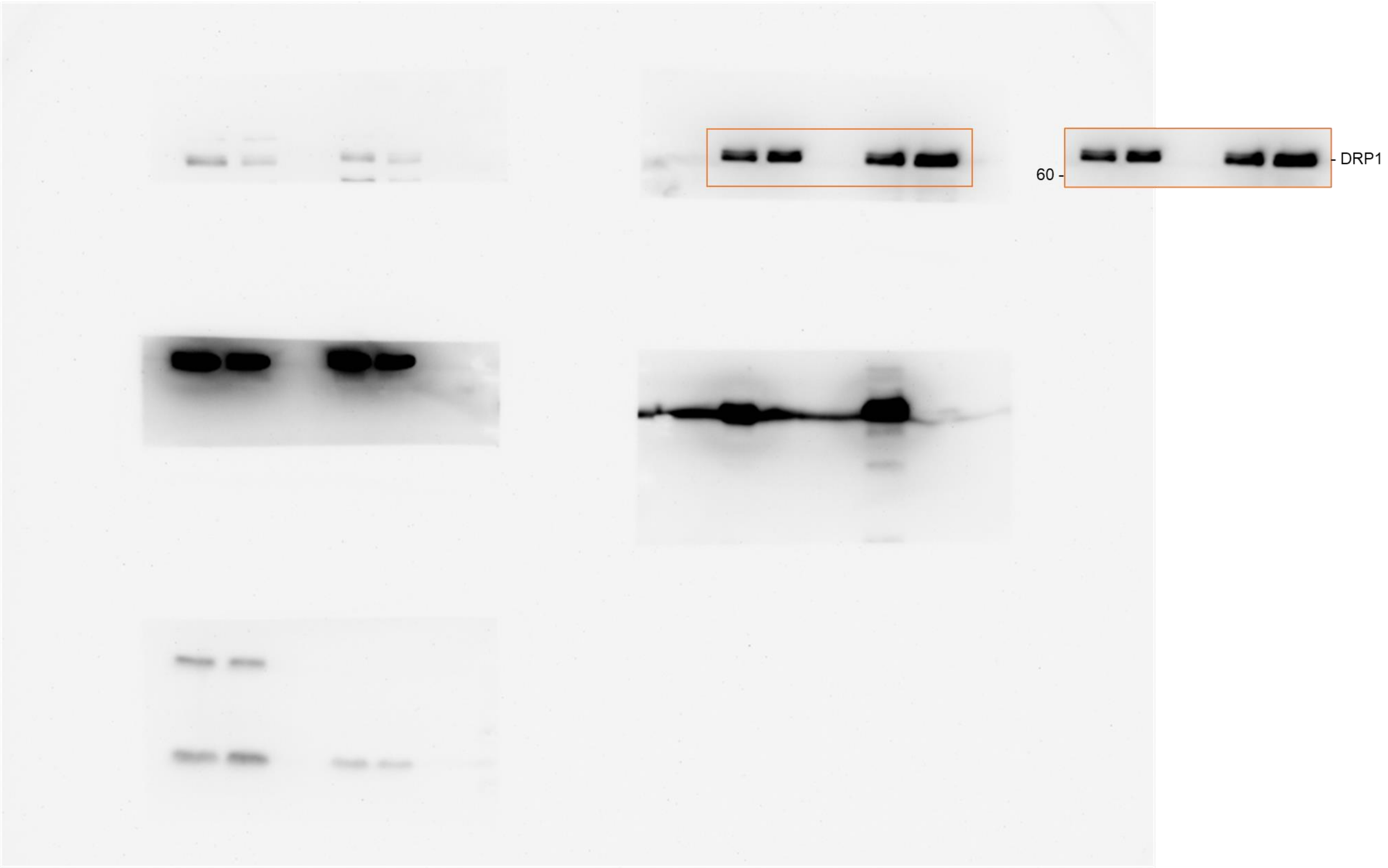

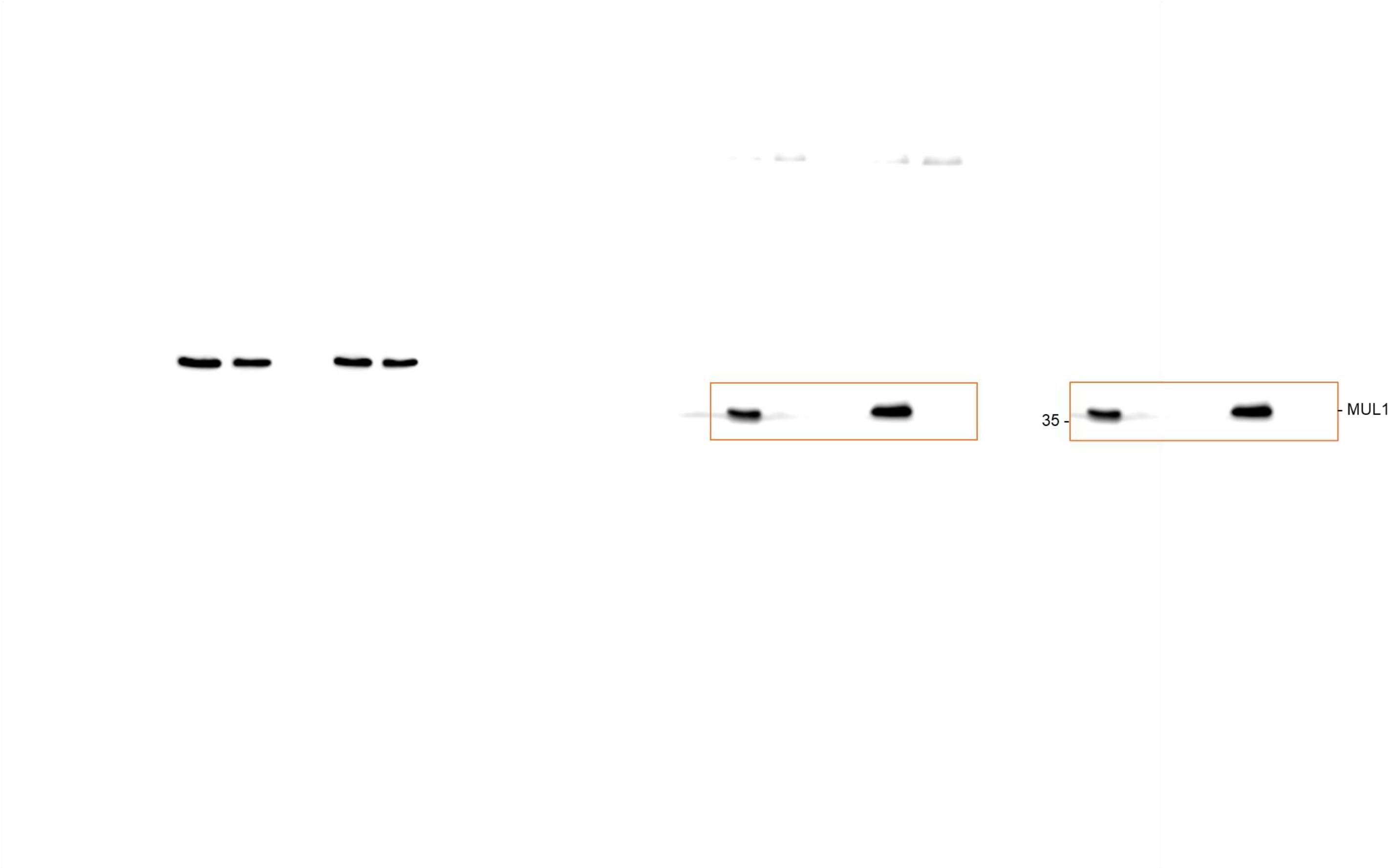

Unprocessed Western blots of Figure 5e

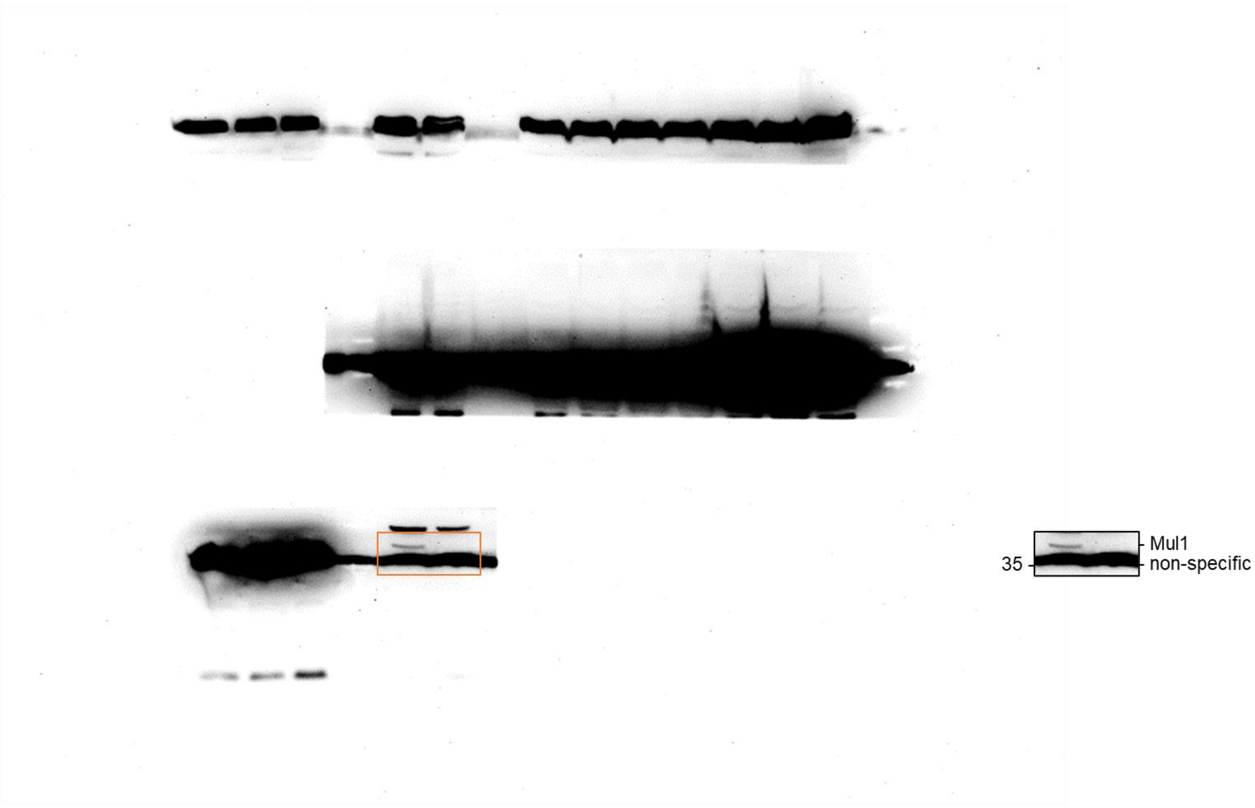

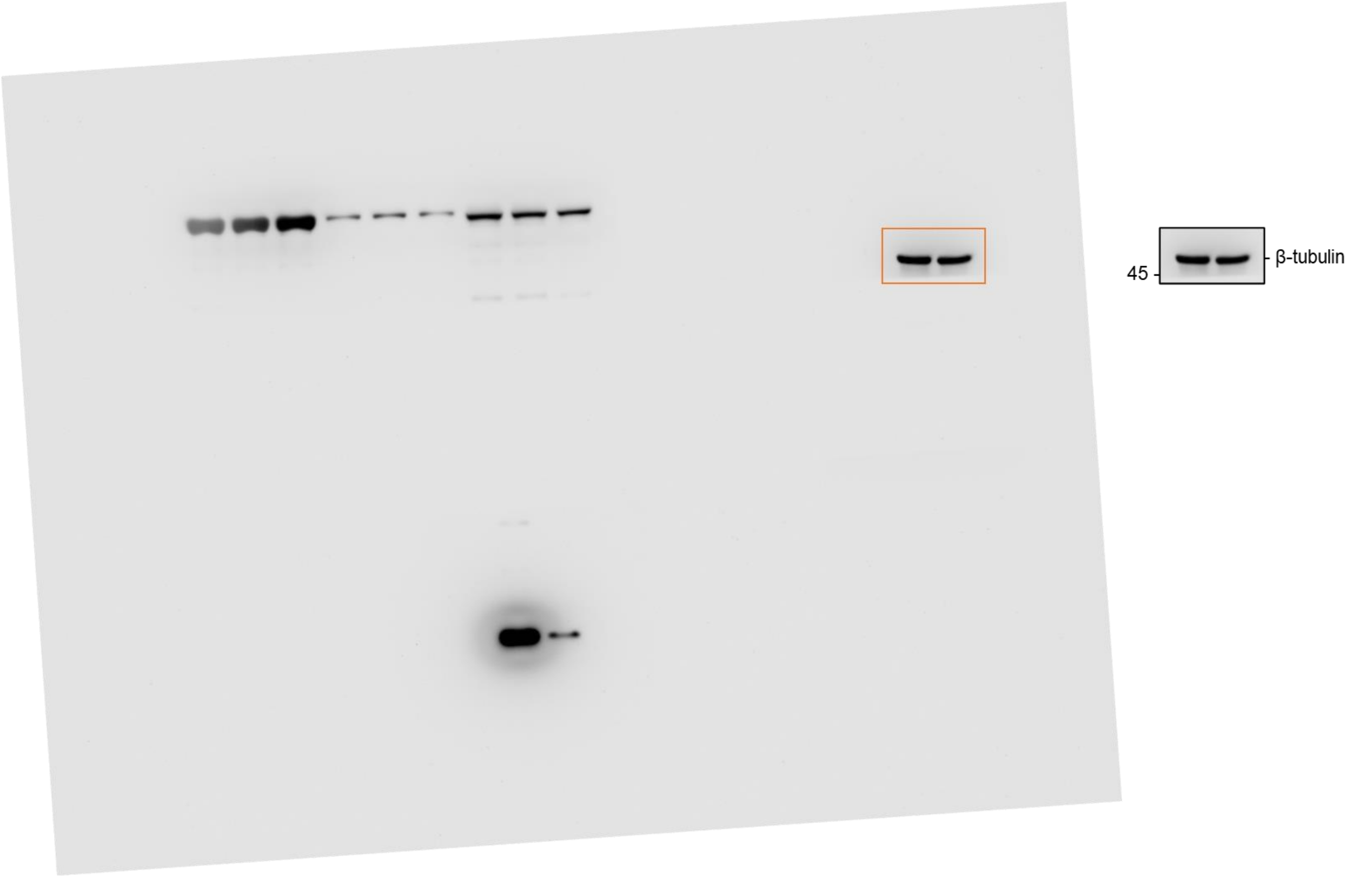

Unprocessed Western blots of Figure 6a

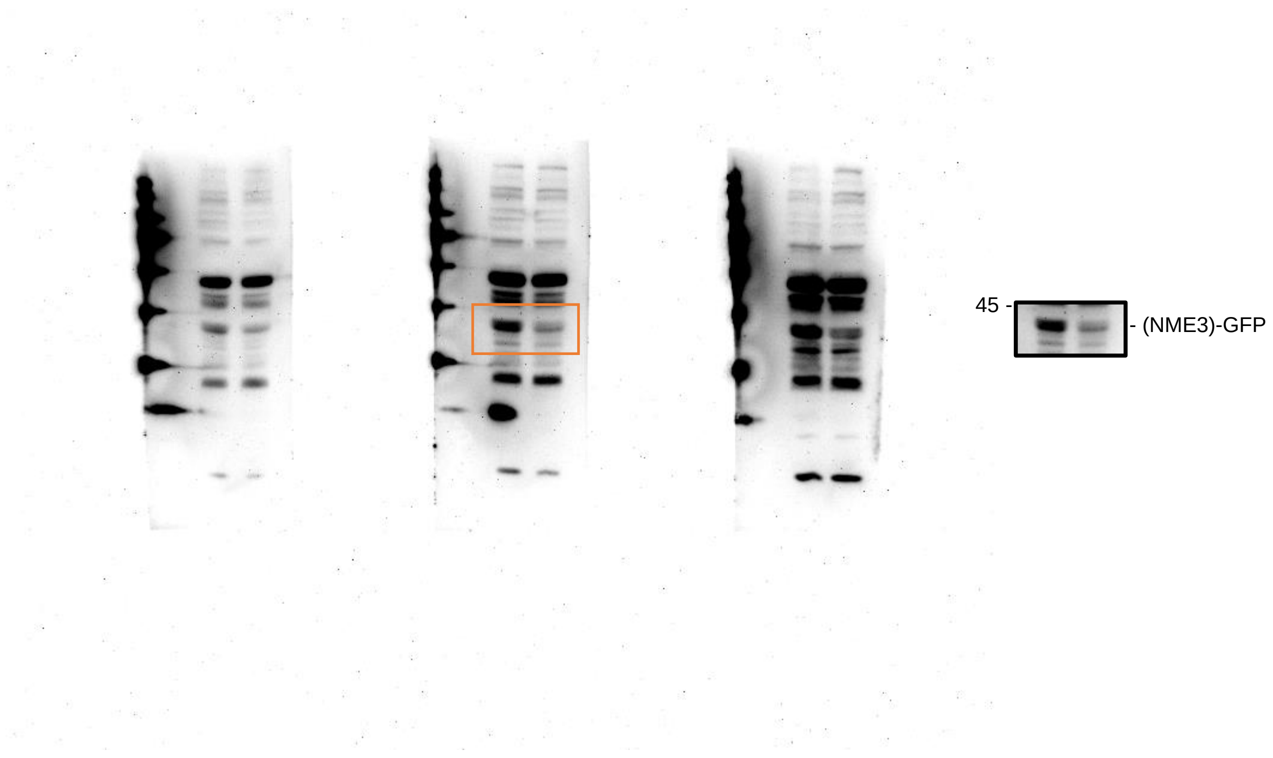

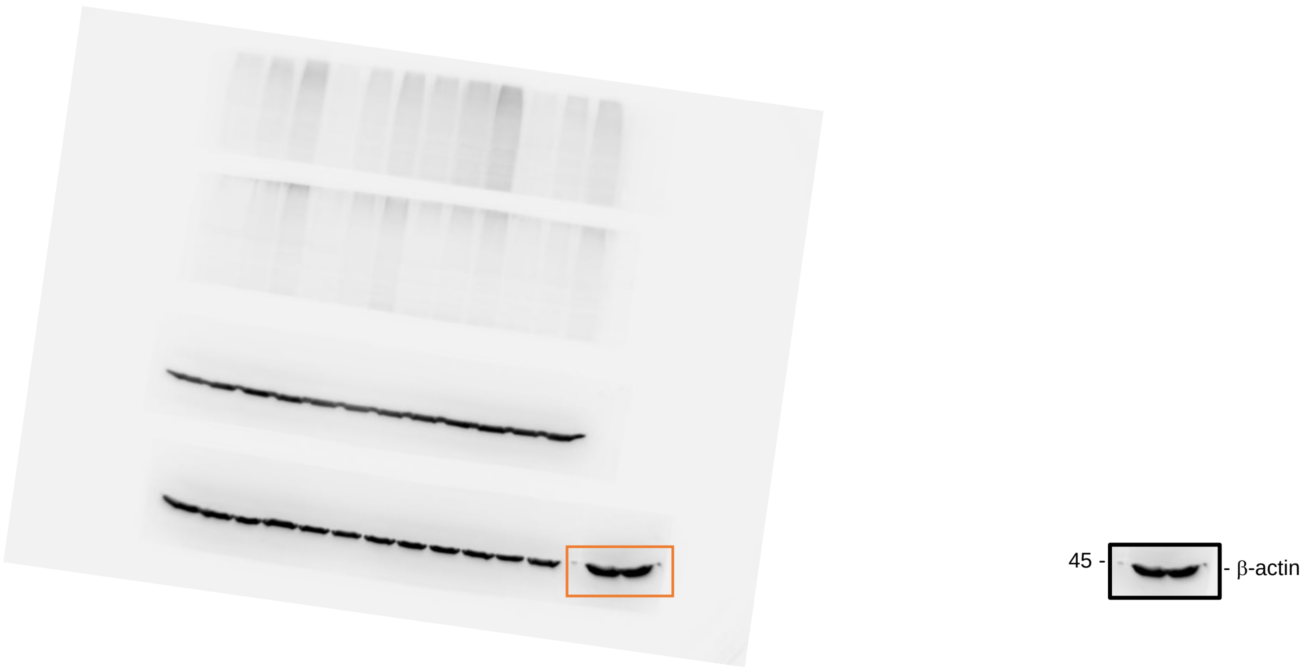

Unprocessed Western blots of Figure 6c

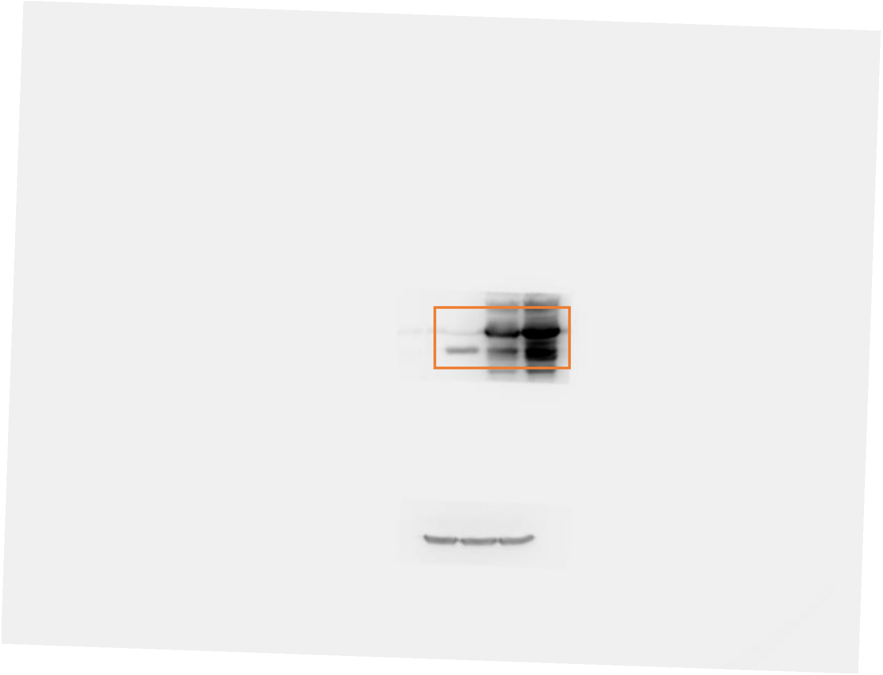

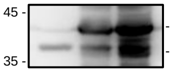

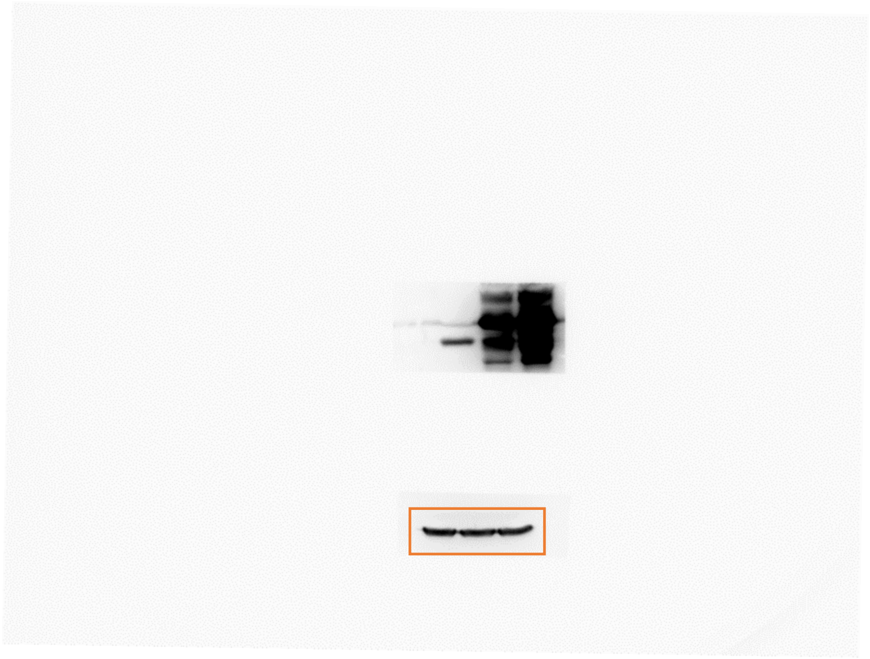

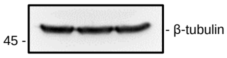

Unprocessed Western blots of Figure 6d

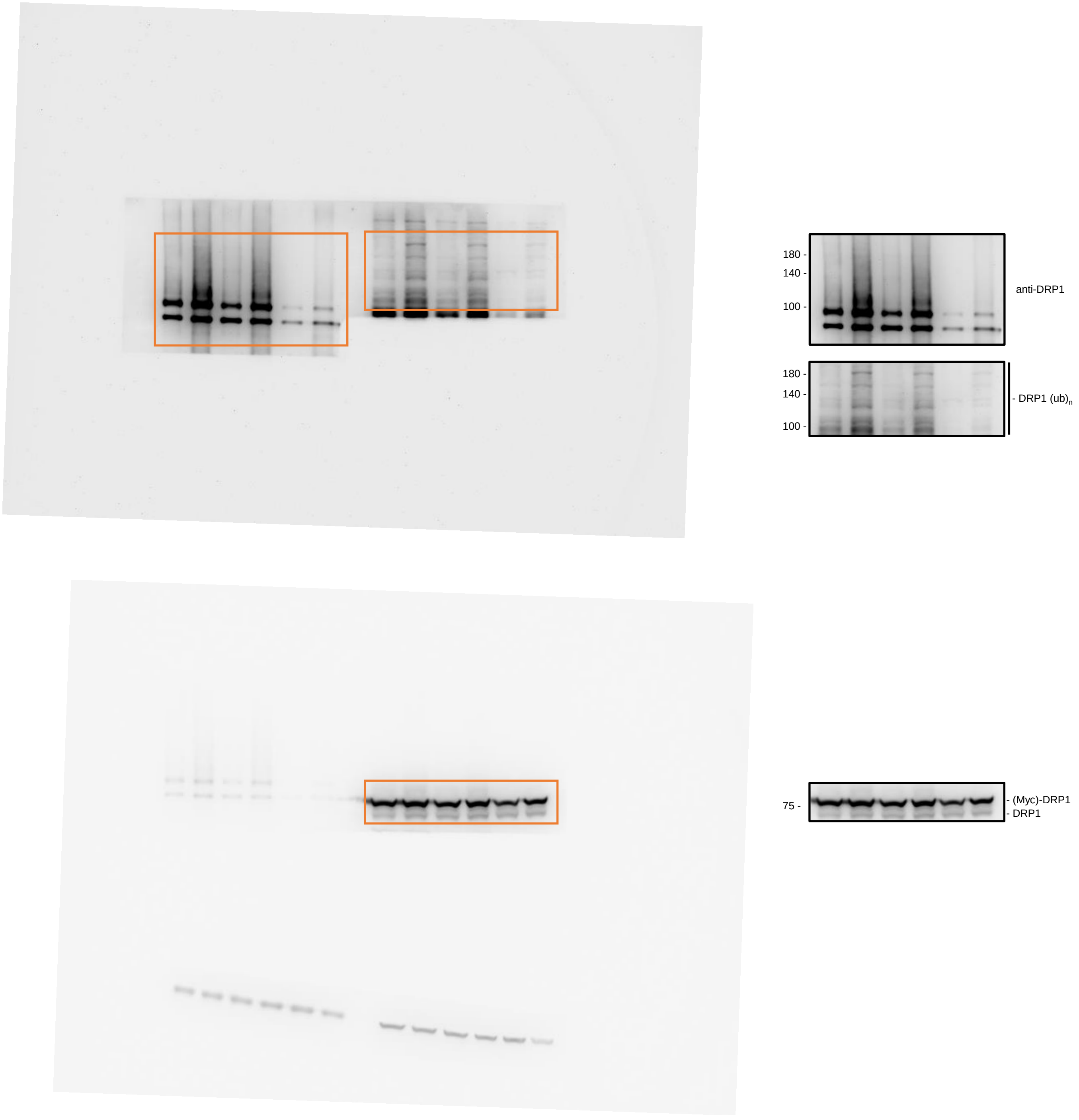

Unprocessed Western blots of Figure 6d (continue)

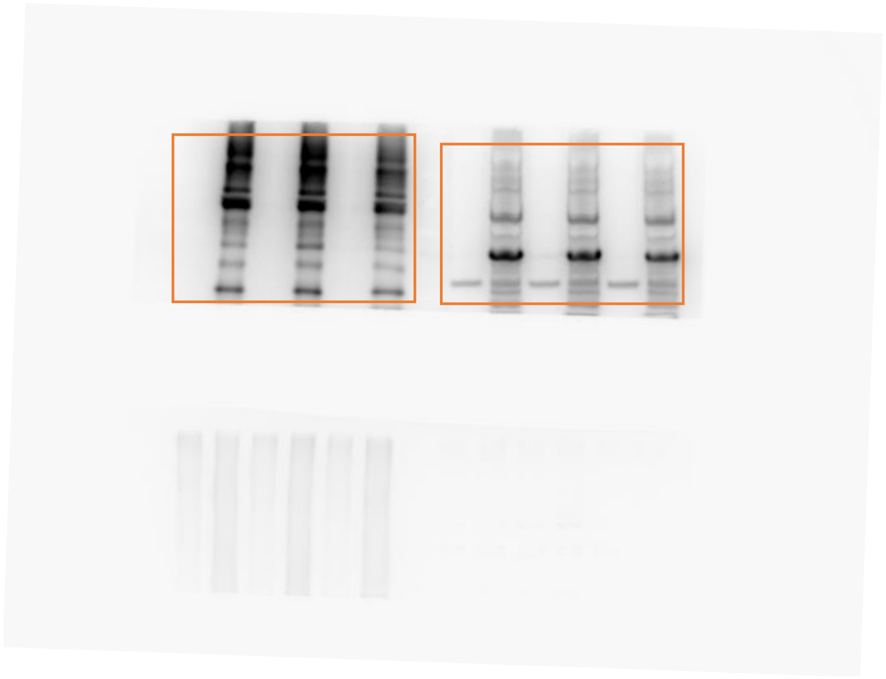

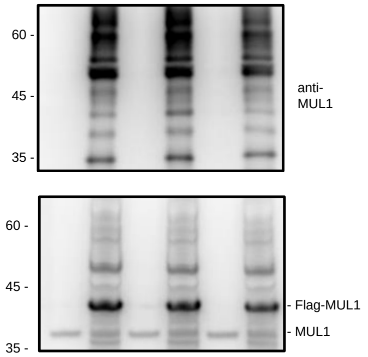

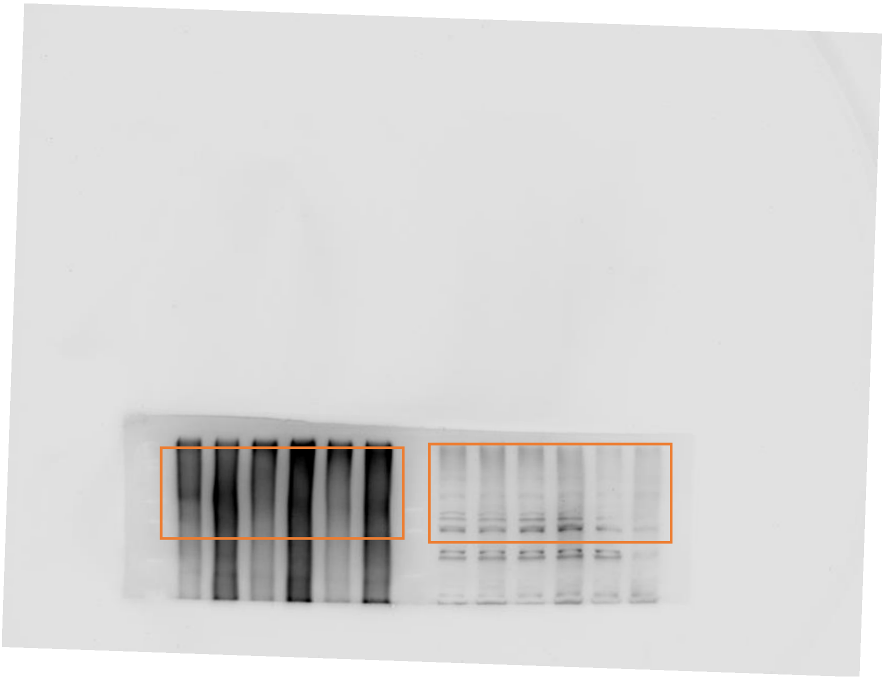

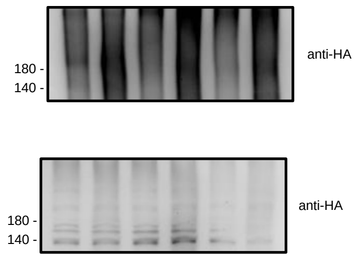

Unprocessed Western blots of Figure 6e

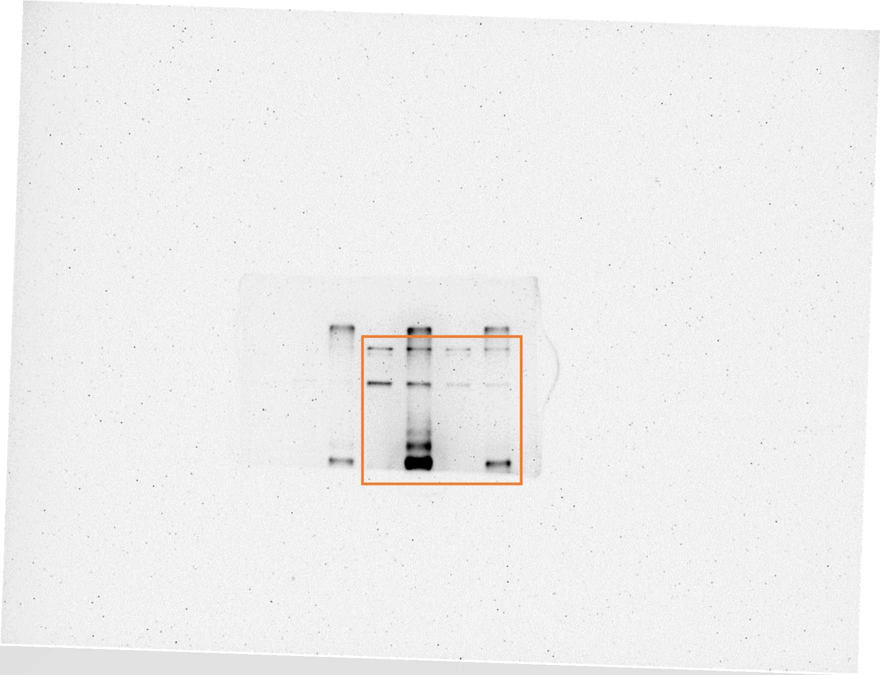

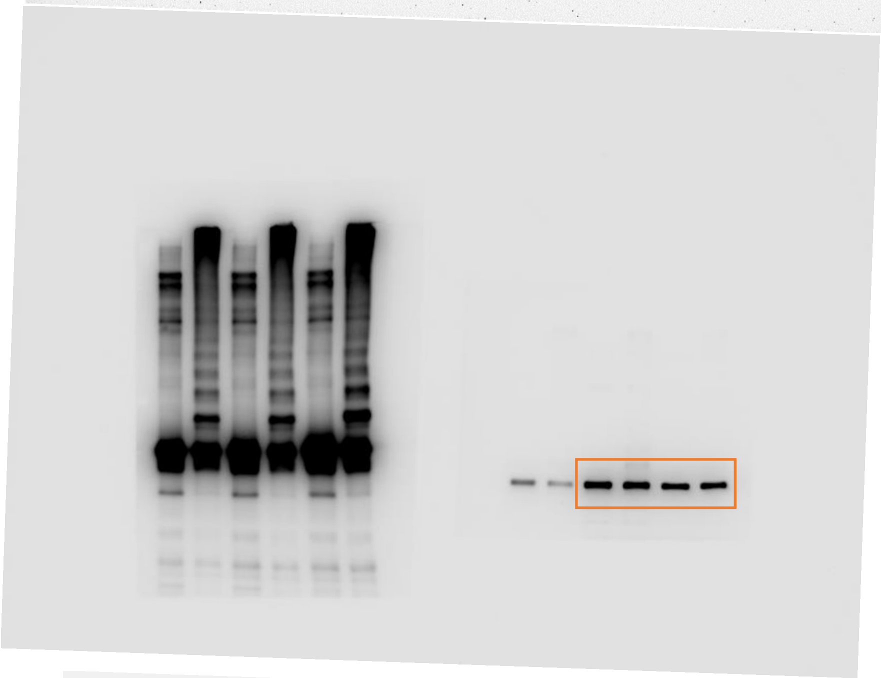

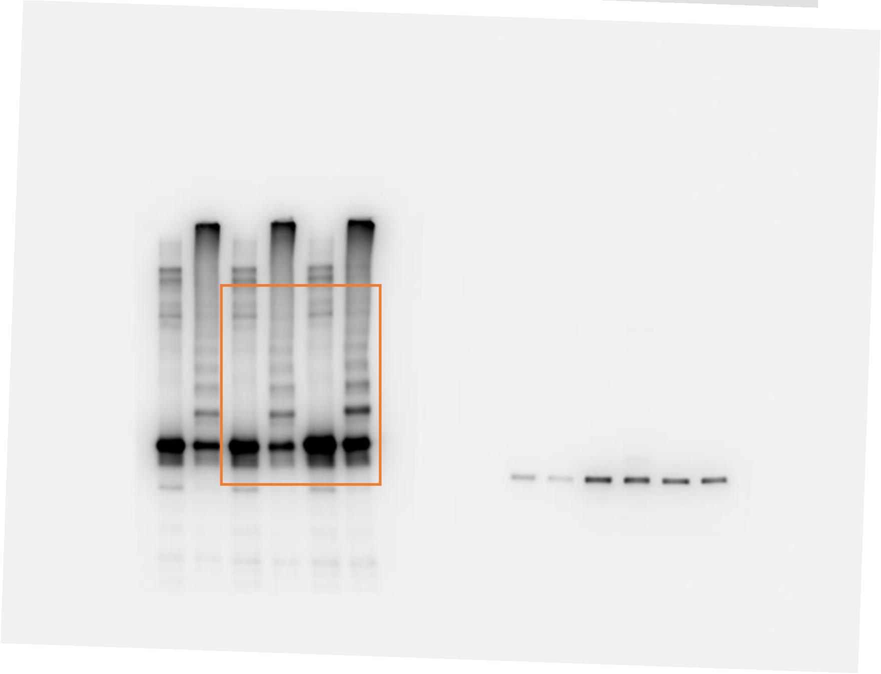

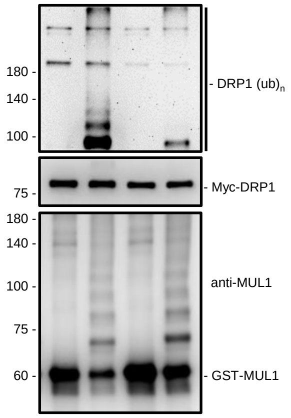

Unprocessed Western blots of Figure 6f

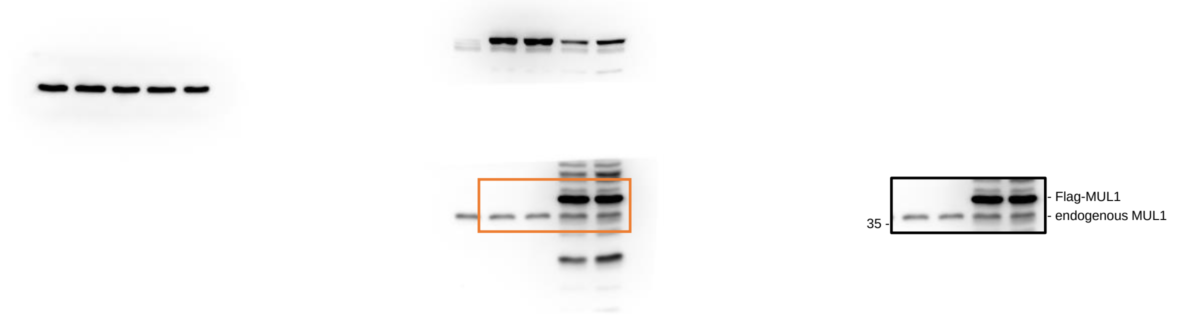

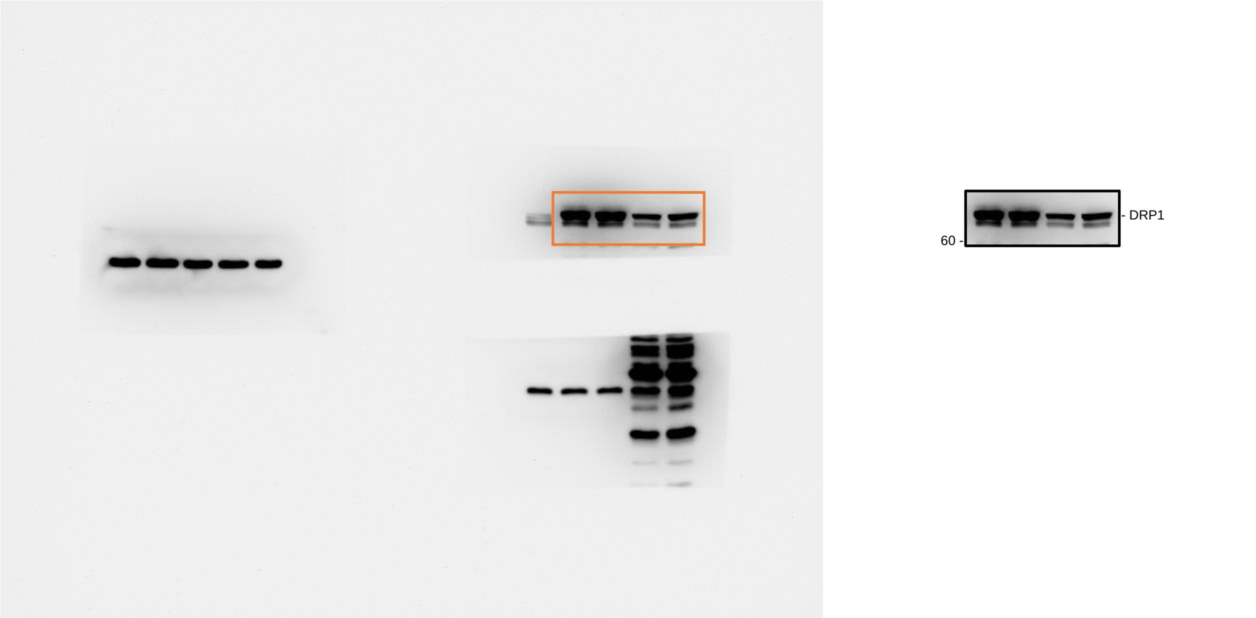

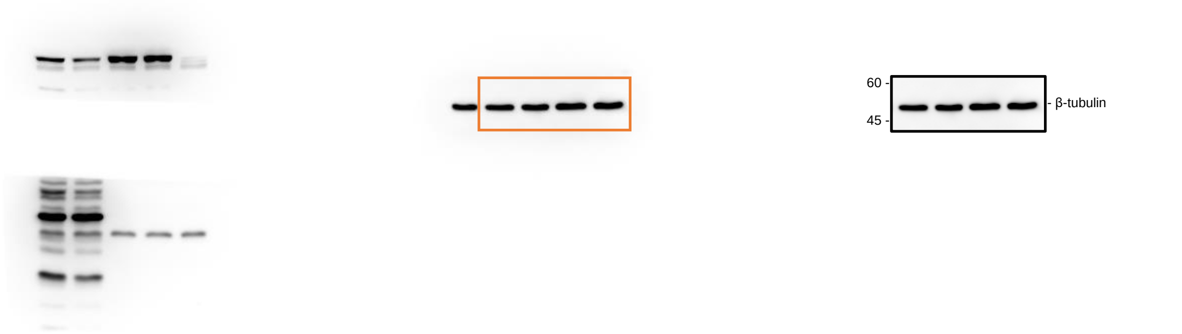

Unprocessed Western blots of Figure 6g

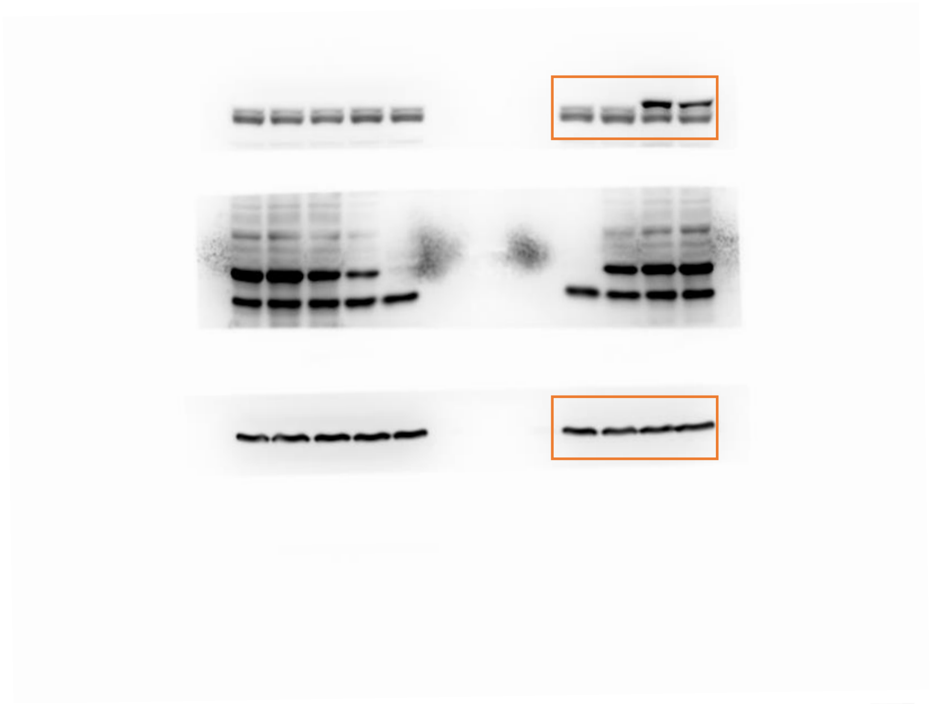

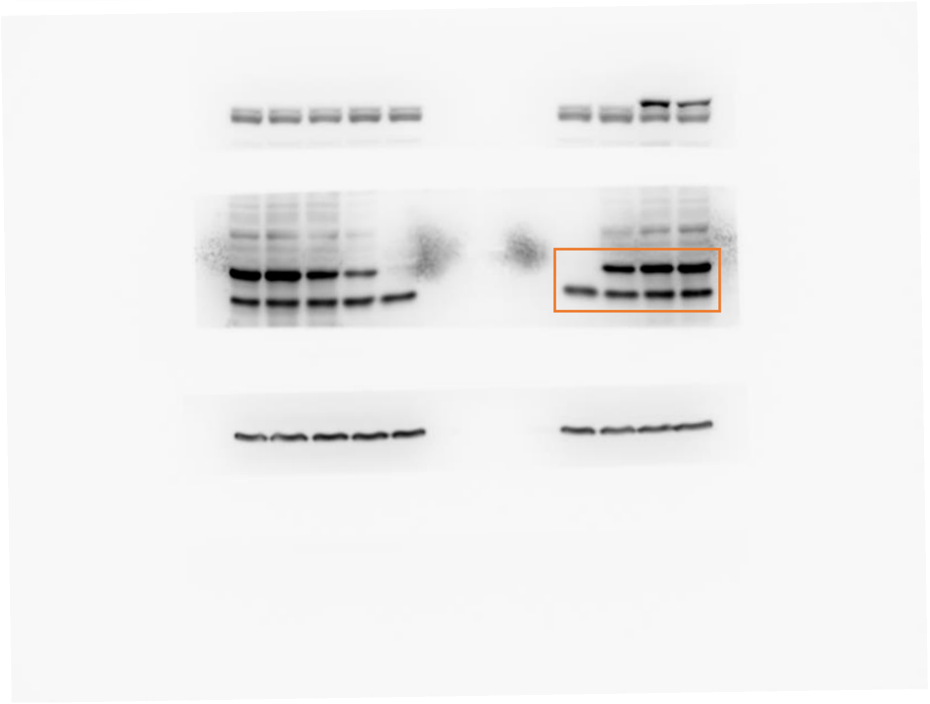

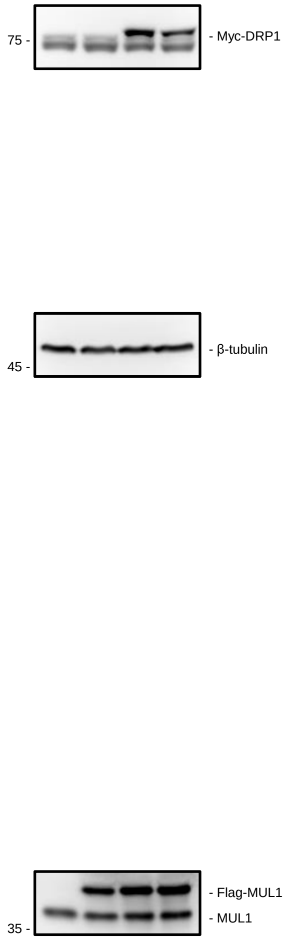

Unprocessed Western blots of Figure 7a

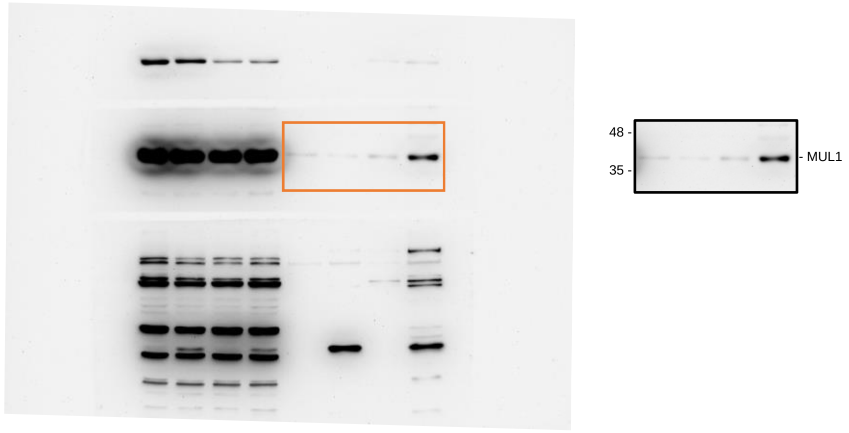

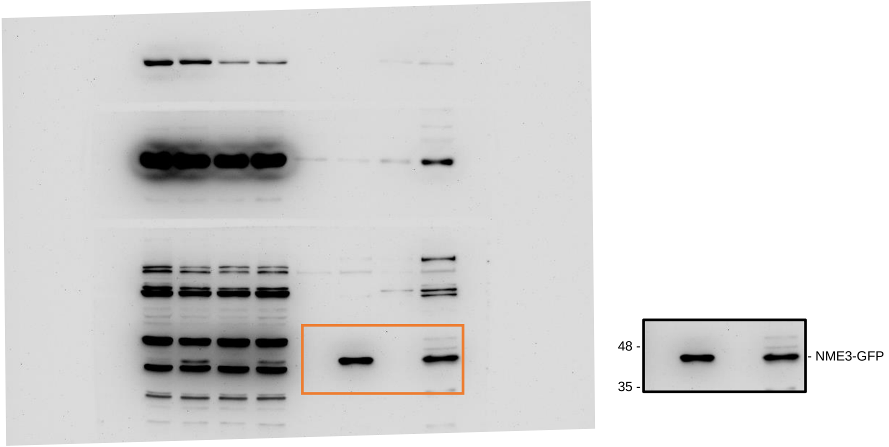

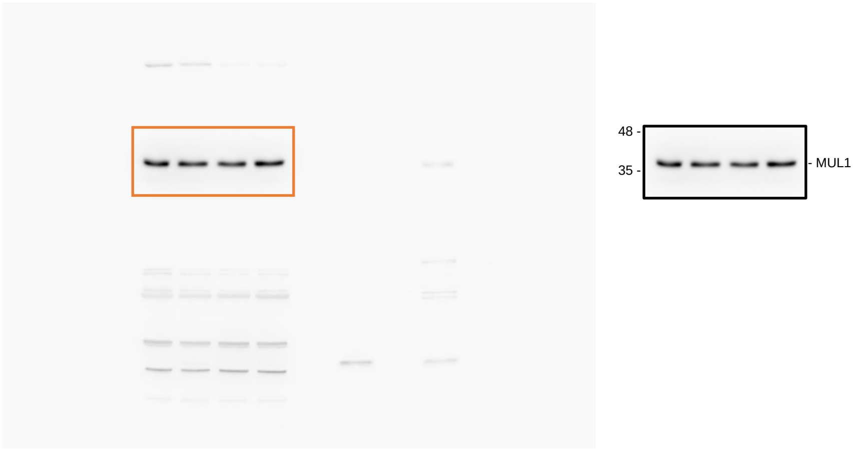

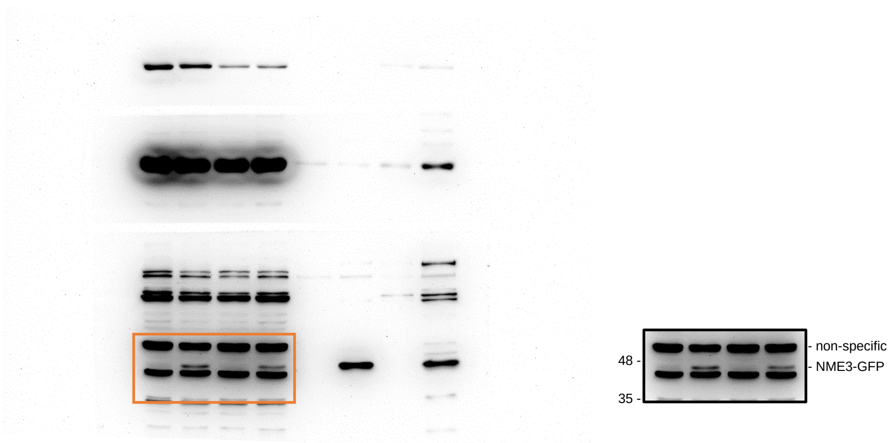

Unprocessed Western blots of Figure 7b

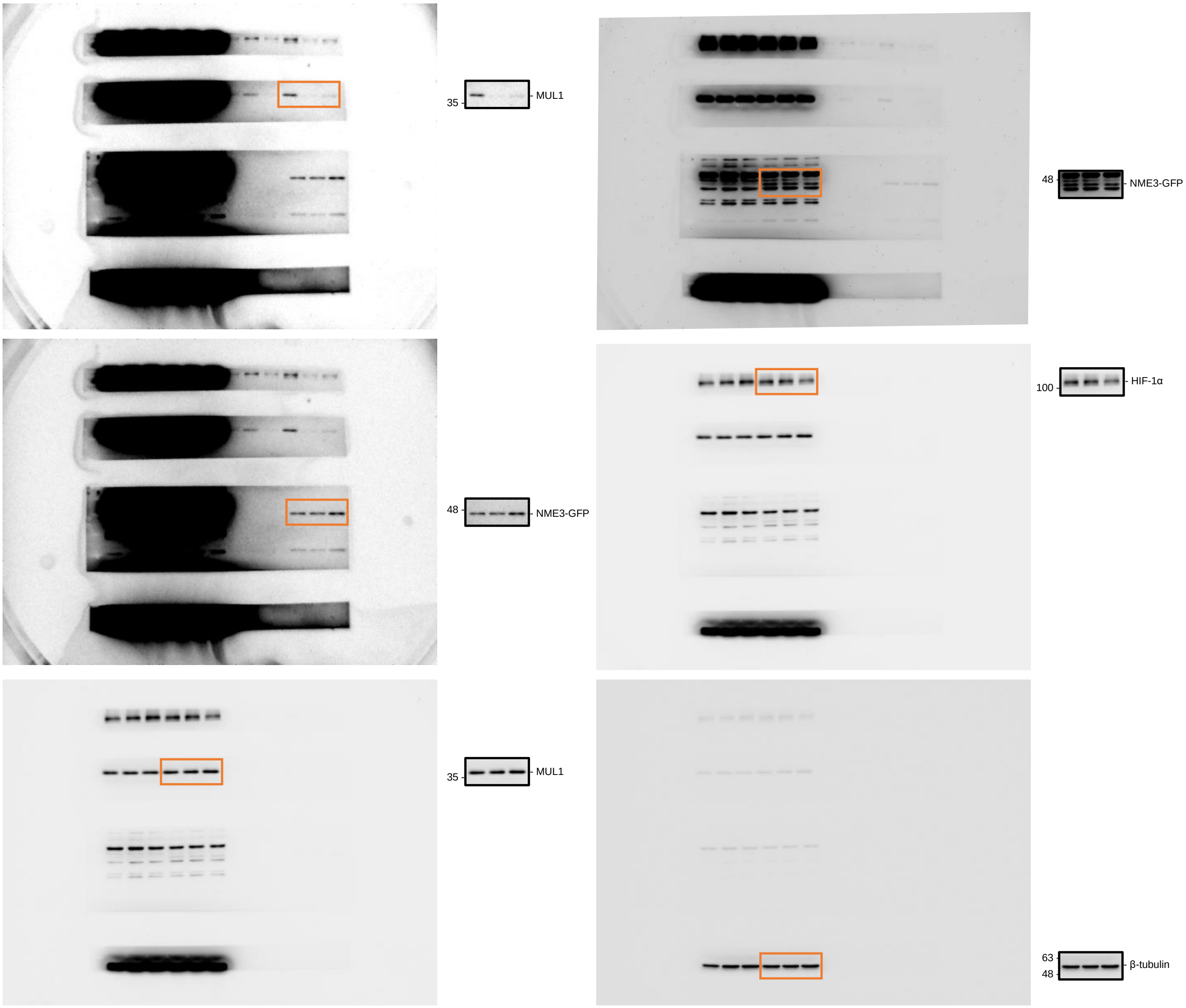

Unprocessed Western blots of Figure 7c

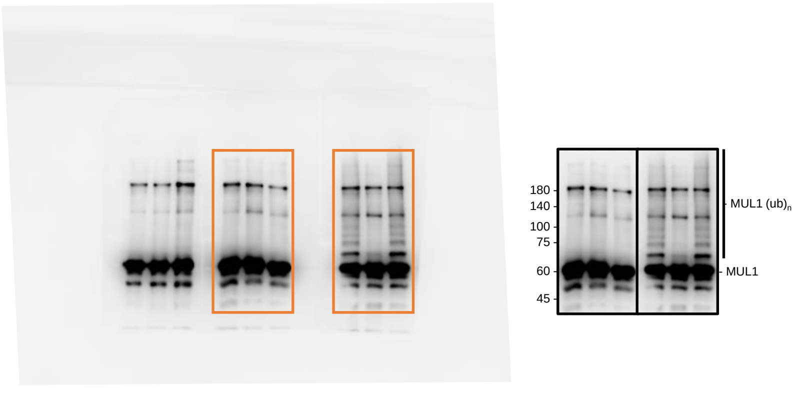

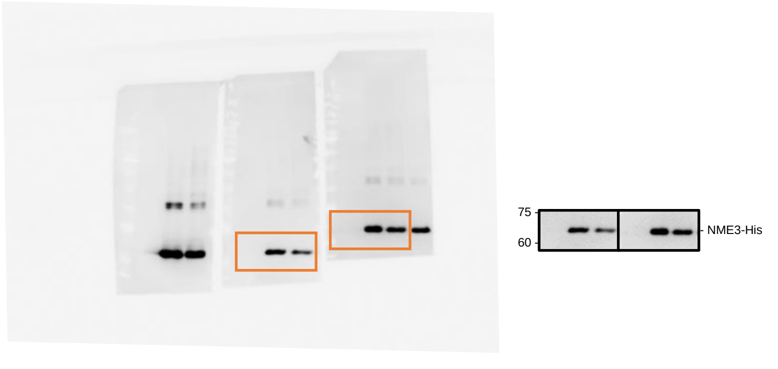

Unprocessed Western blots of Figure 7e

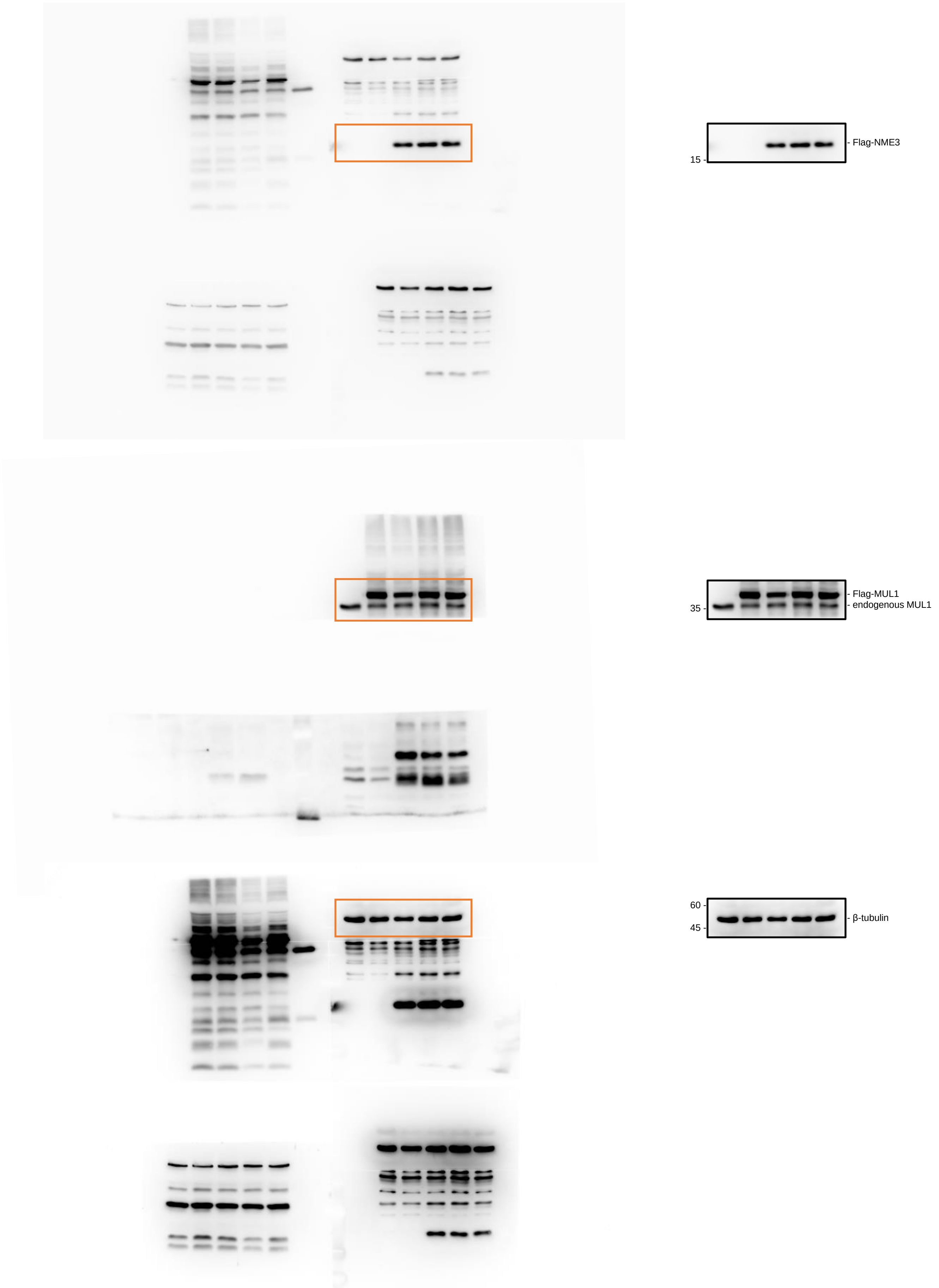

Unprocessed Western blots of Figure 8a

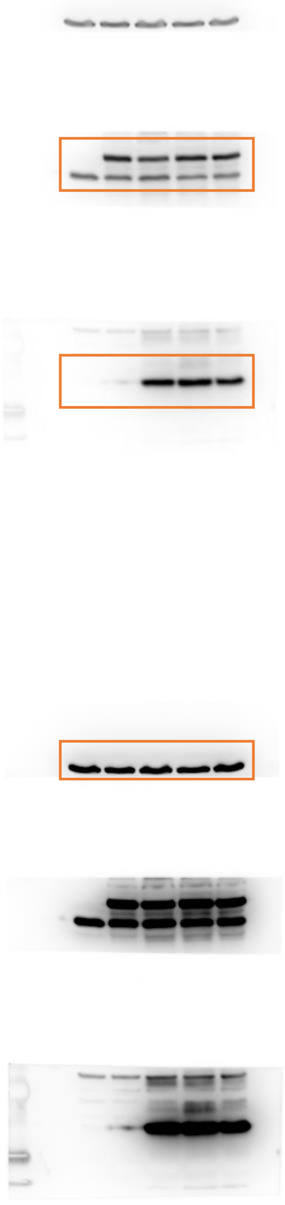

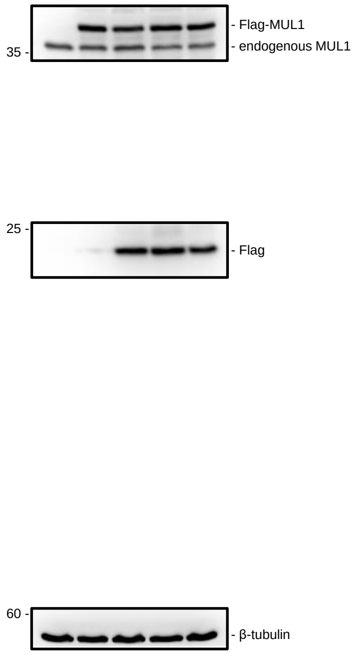

Unprocessed Western blots of Figure 8b

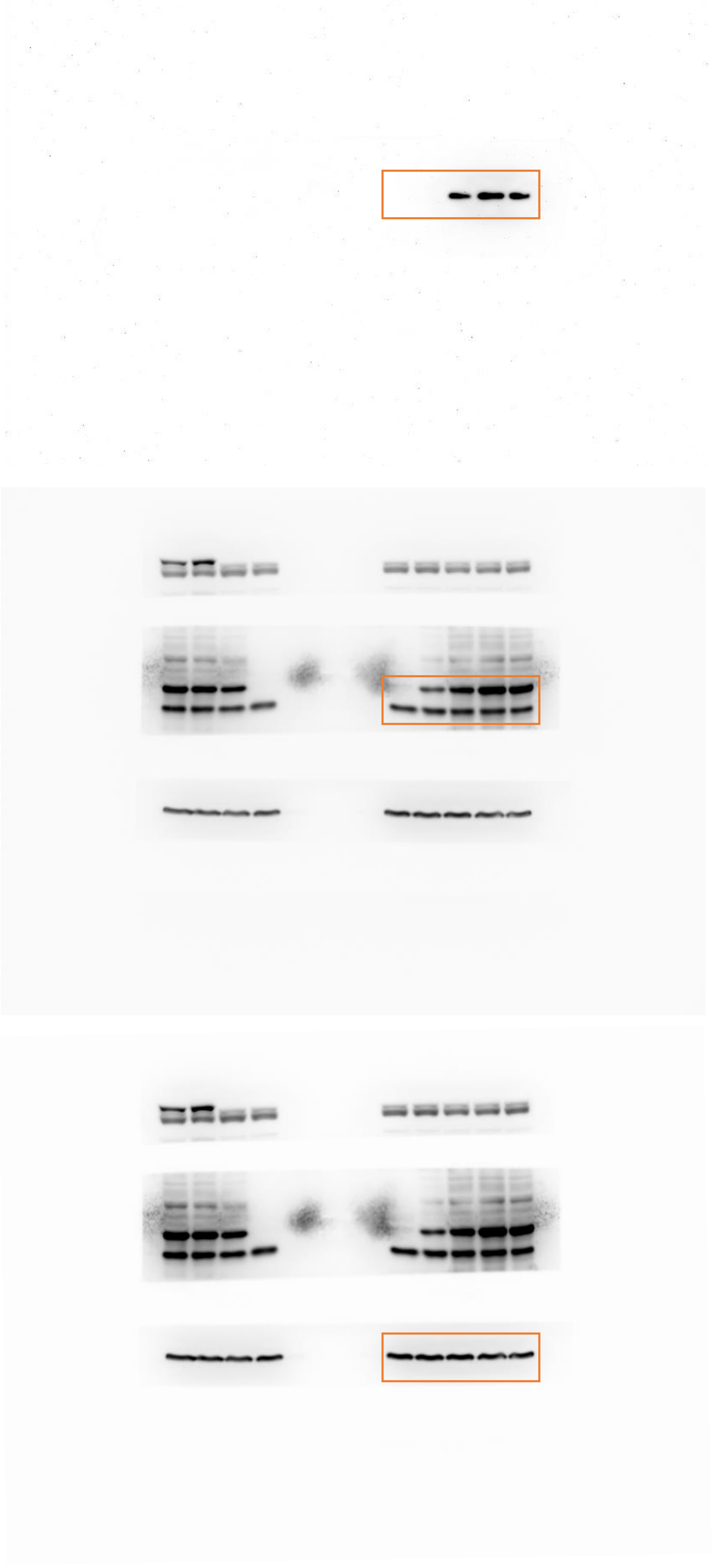

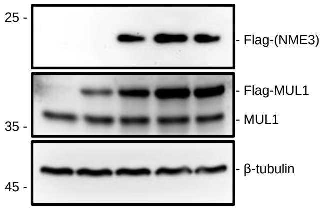

Unprocessed Western blots of Figure S1b

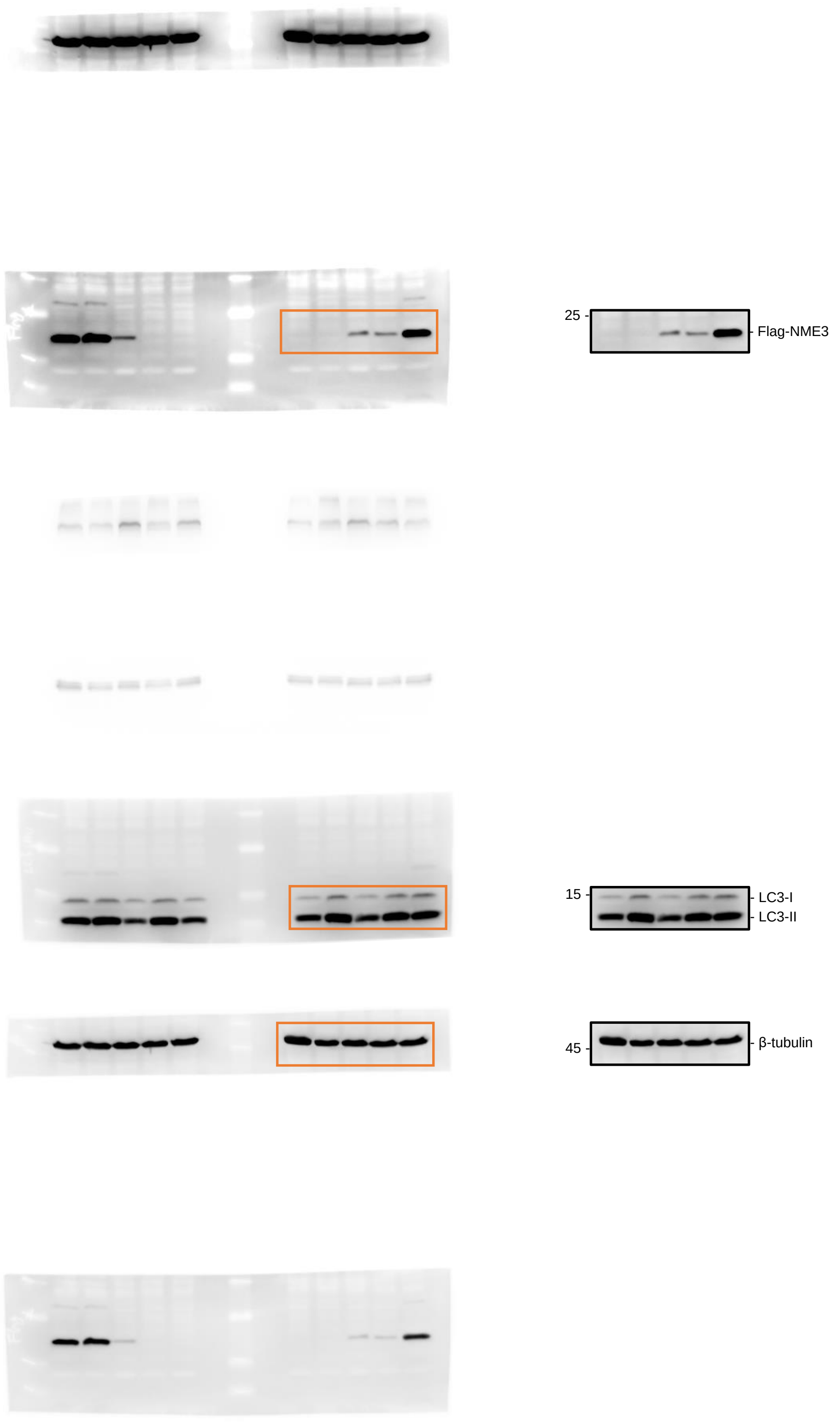

Unprocessed Western blots of Figure S1c

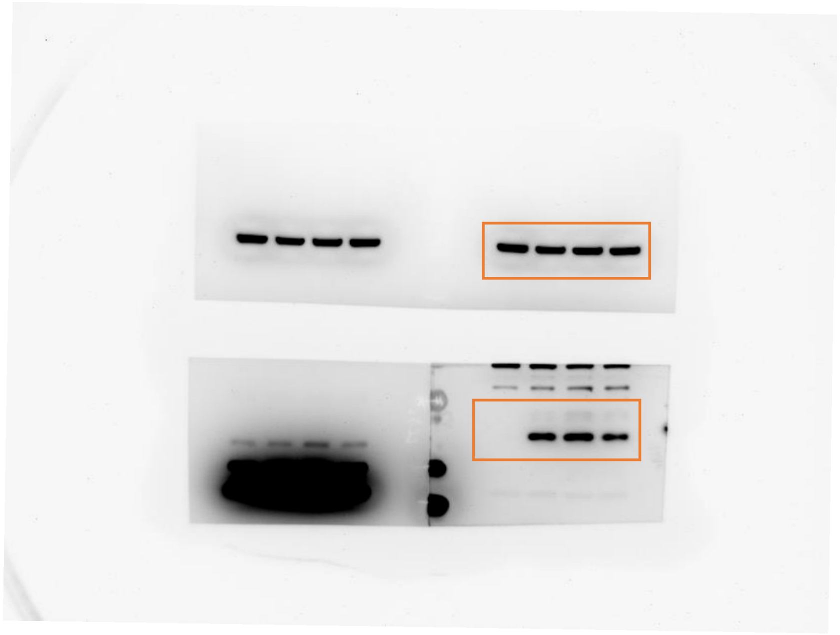

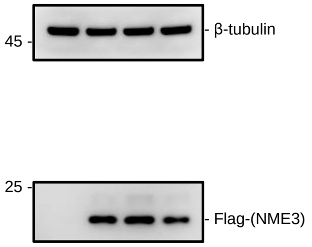

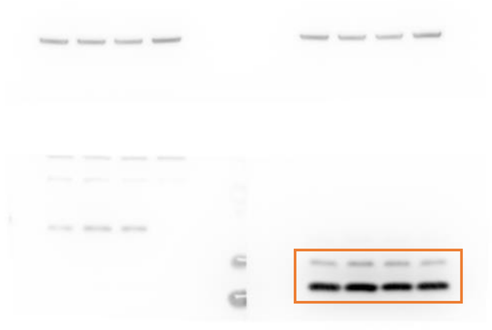

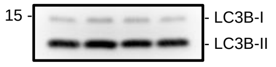

Unprocessed Western blots of Figure S2g

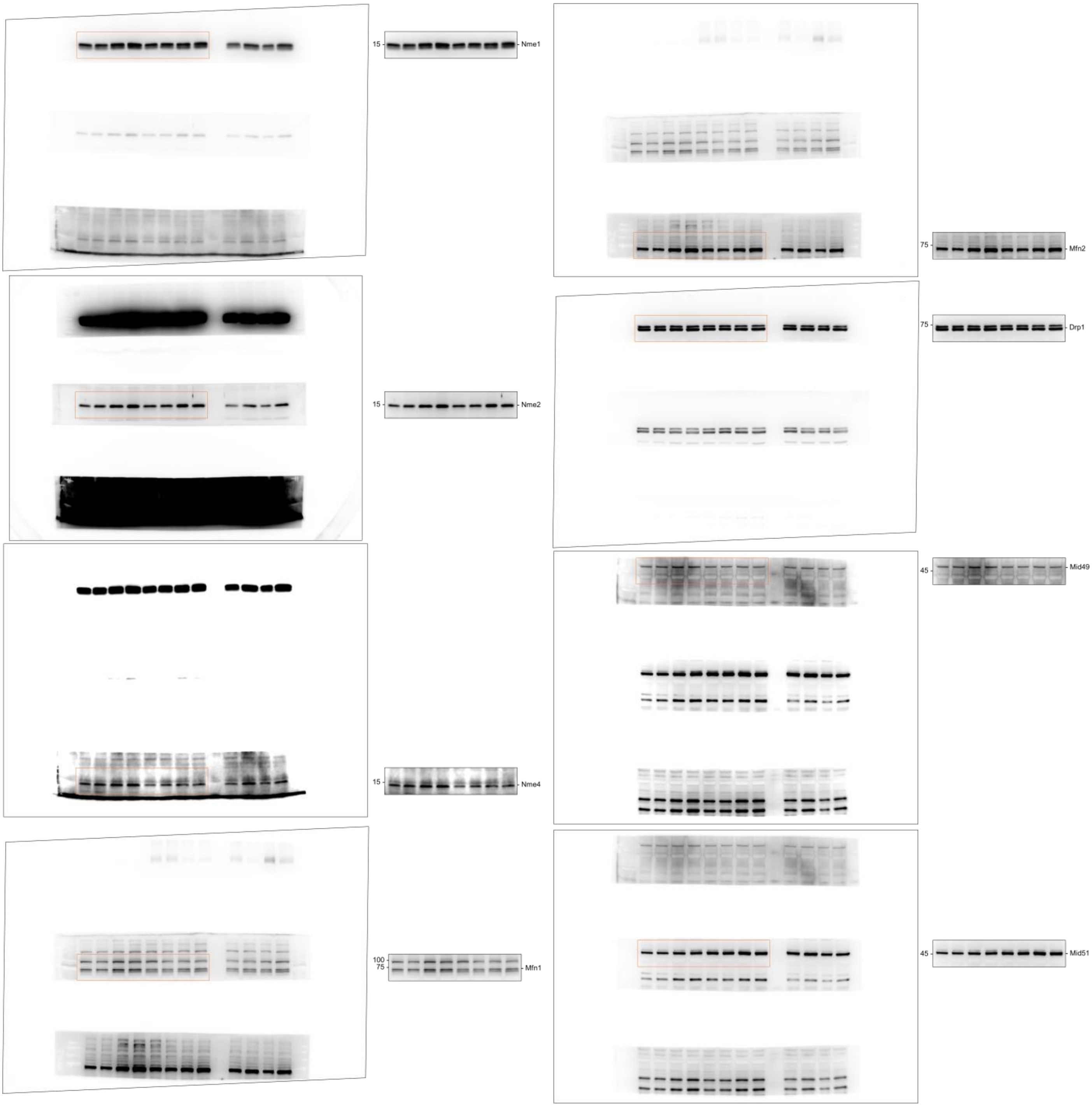

Unprocessed Western blots of Figure S2g (continue)

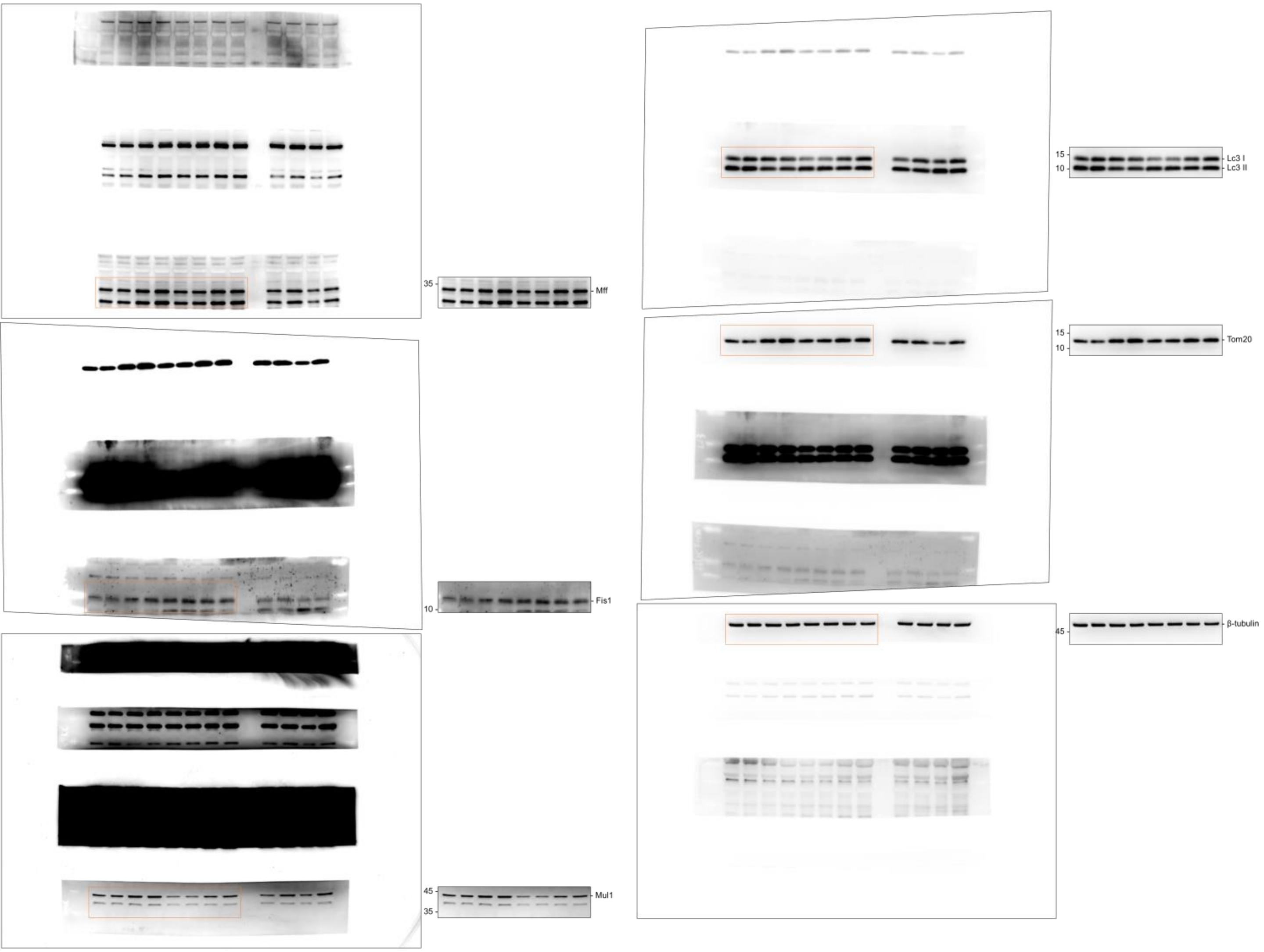

Unprocessed Western blots of Figure S2h

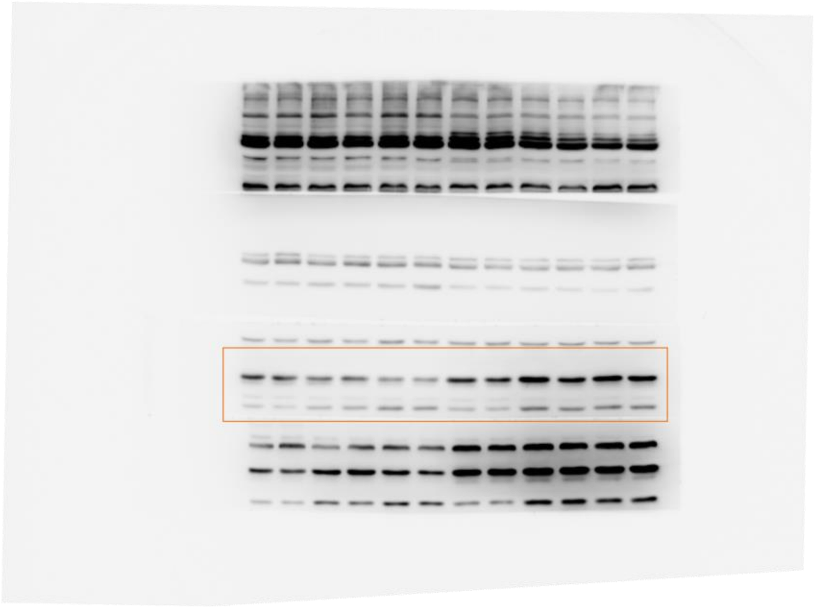

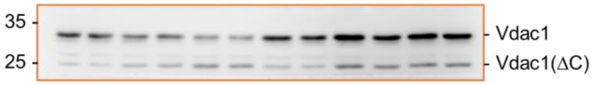

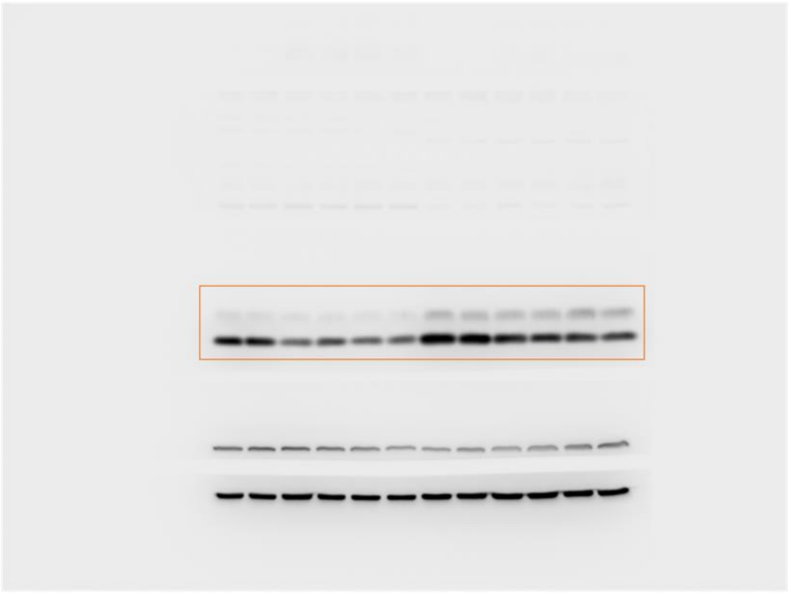

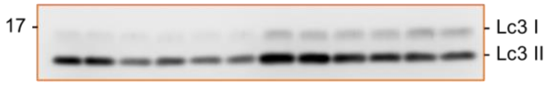

Unprocessed Western blots of Figure S2h

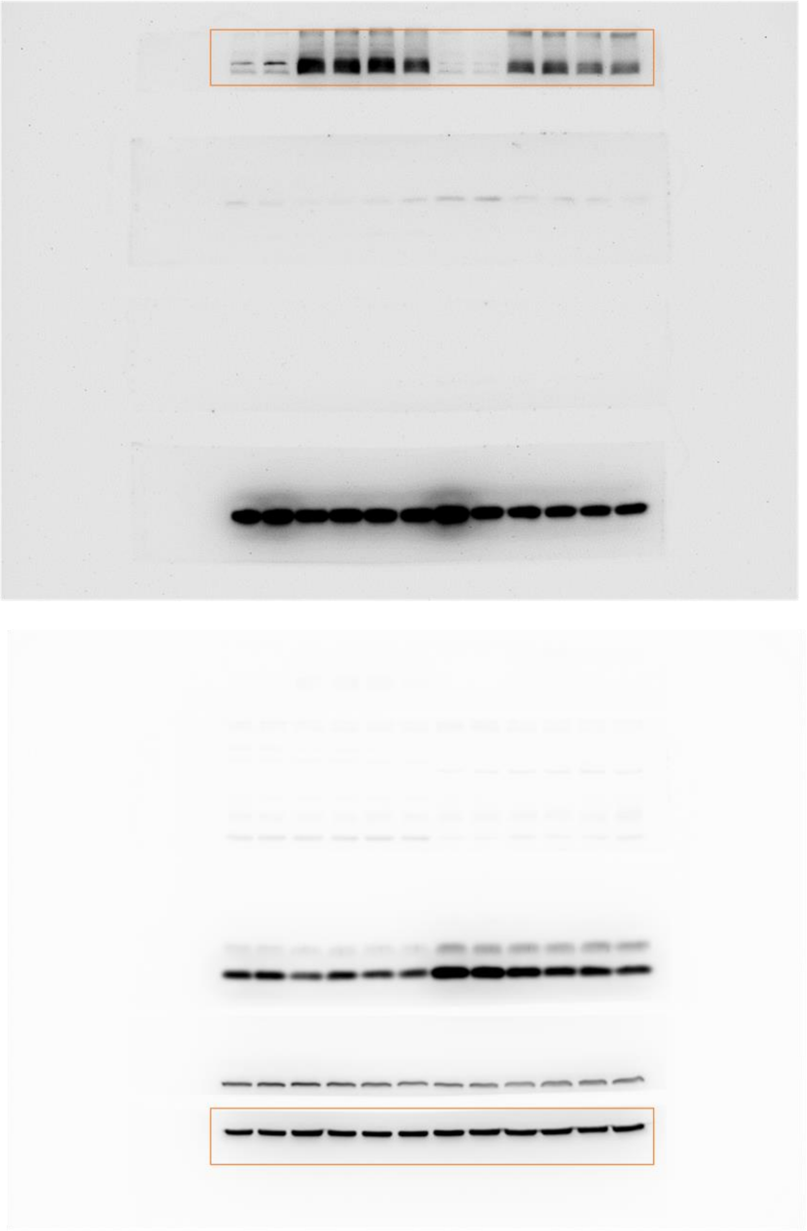

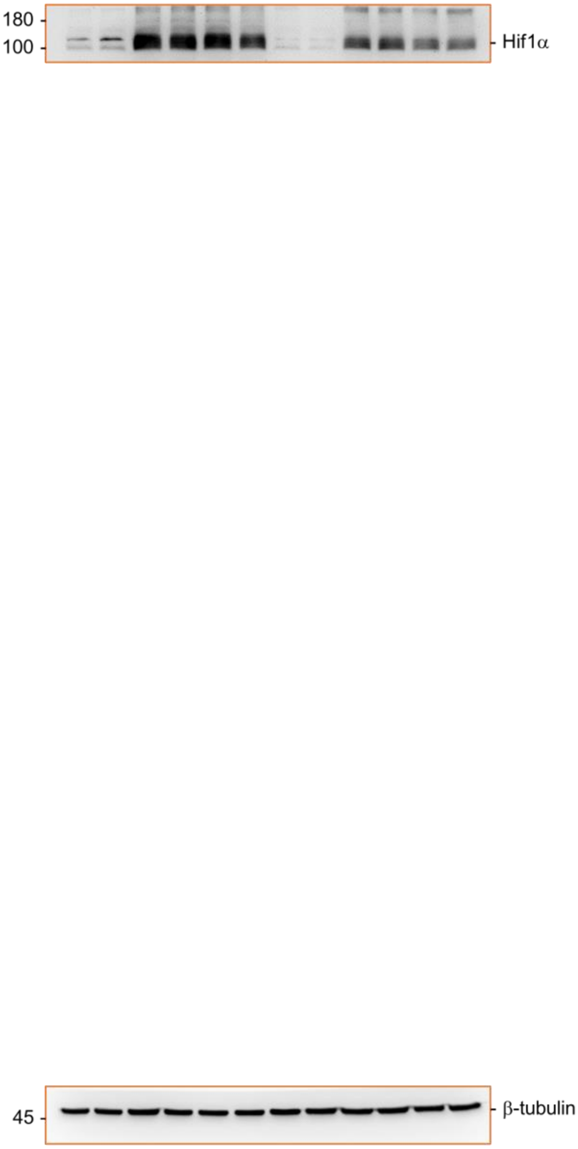

Unprocessed Western blots of Figure S3a

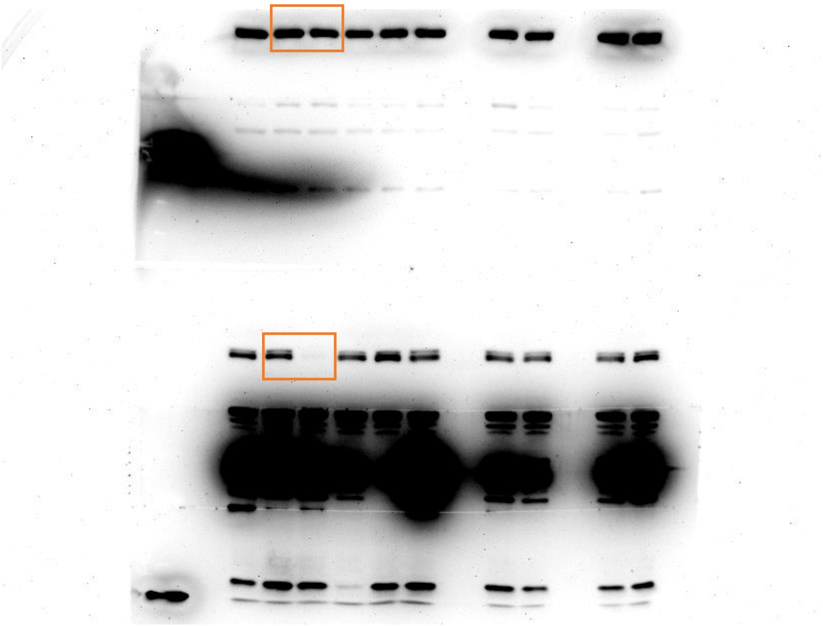

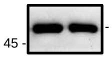

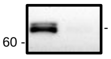

Unprocessed Western blots of Figure S3a (continue)

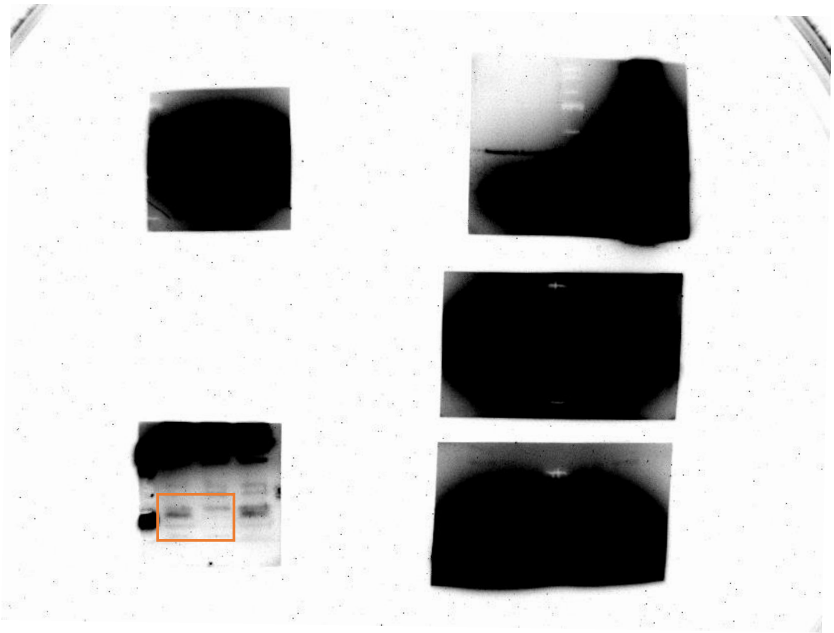

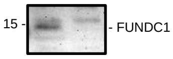

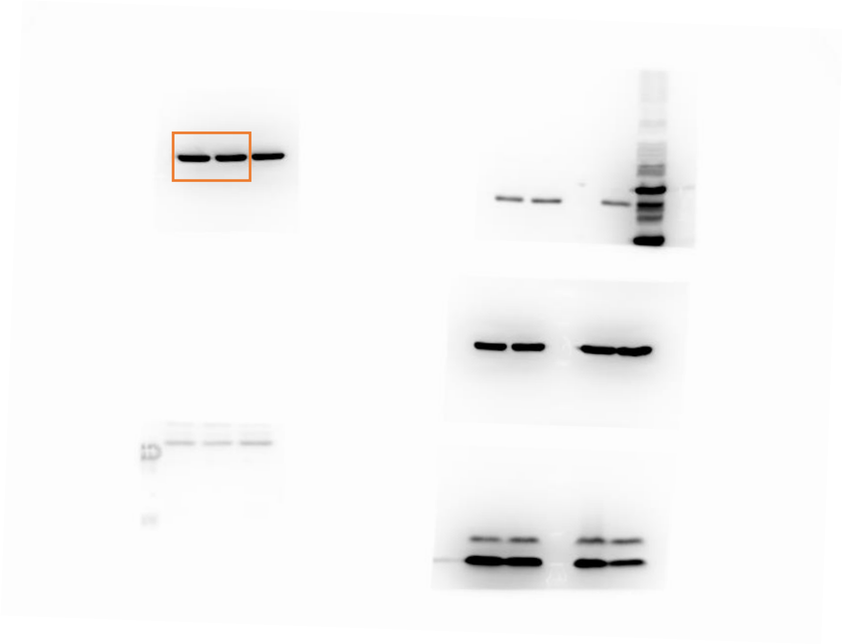

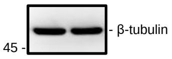

Unprocessed Western blots of Figure S3c

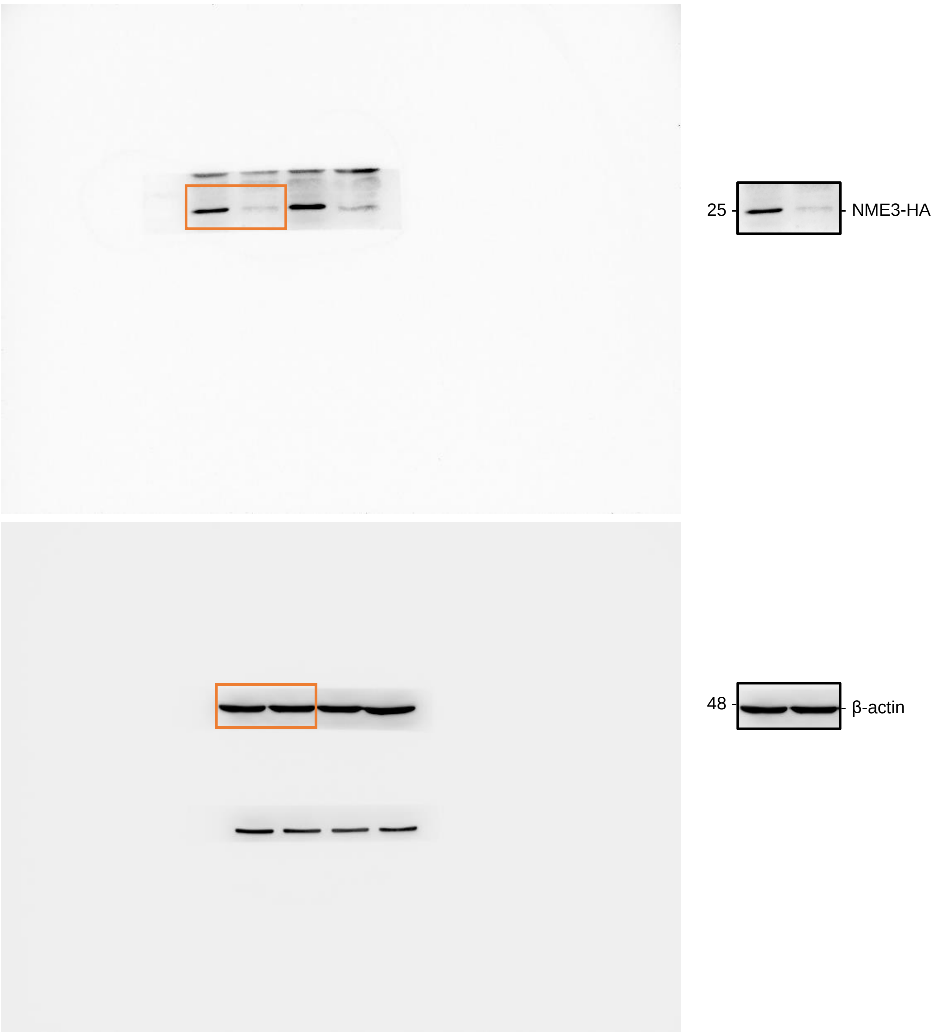

Unprocessed Western blots of Figure S3d

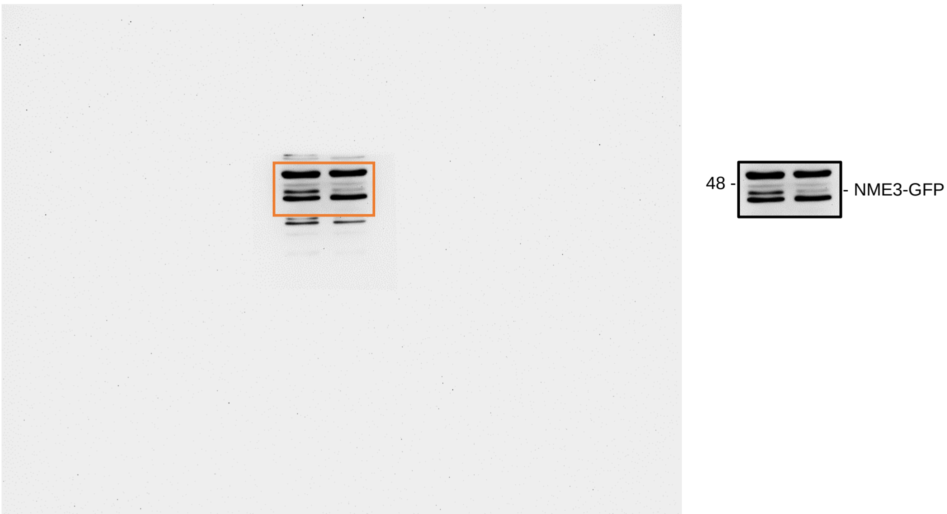

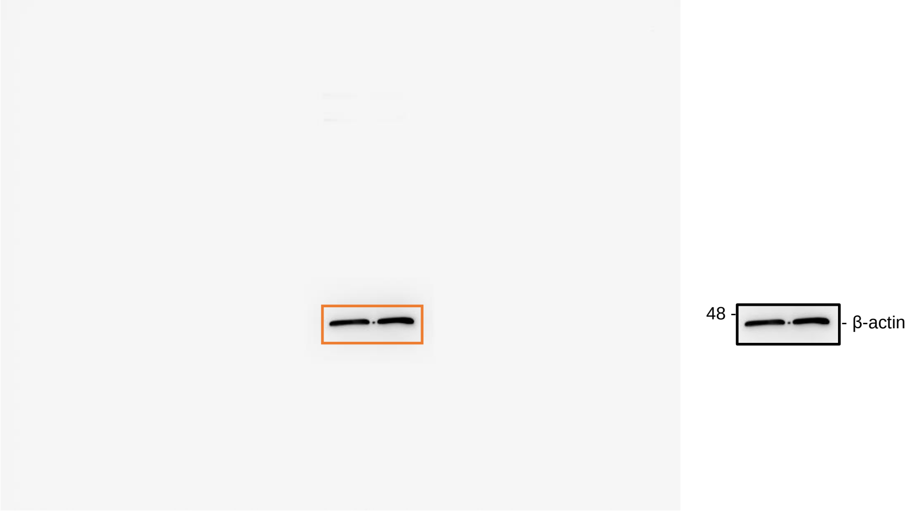

Unprocessed Western blots of Figure S4b

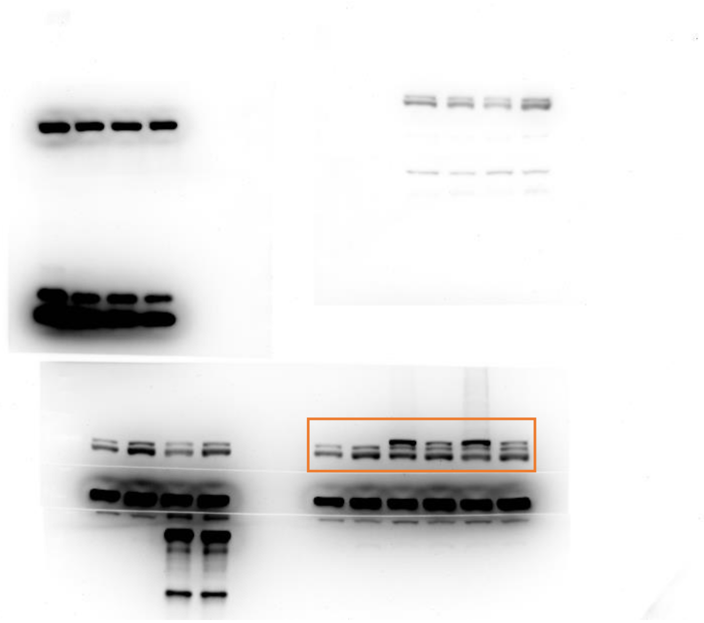

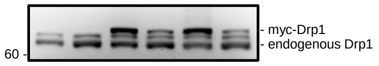

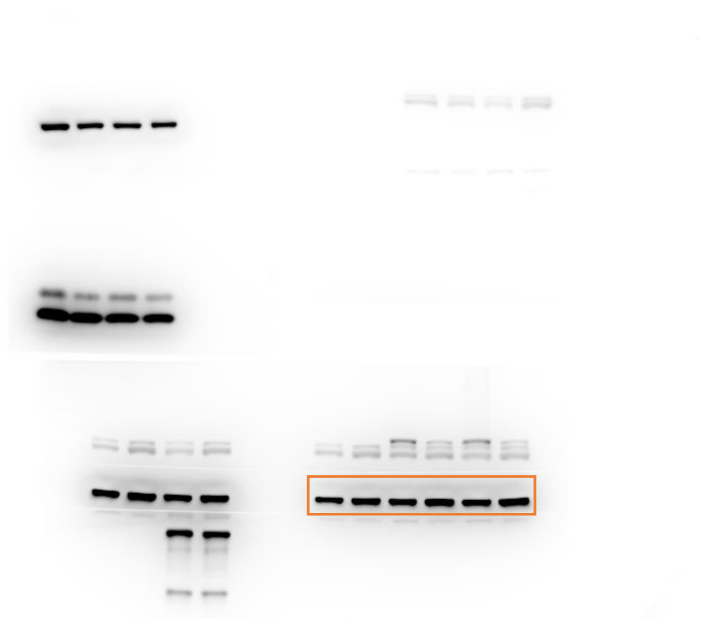

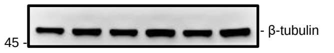

Unprocessed Western blots of Figure S5b

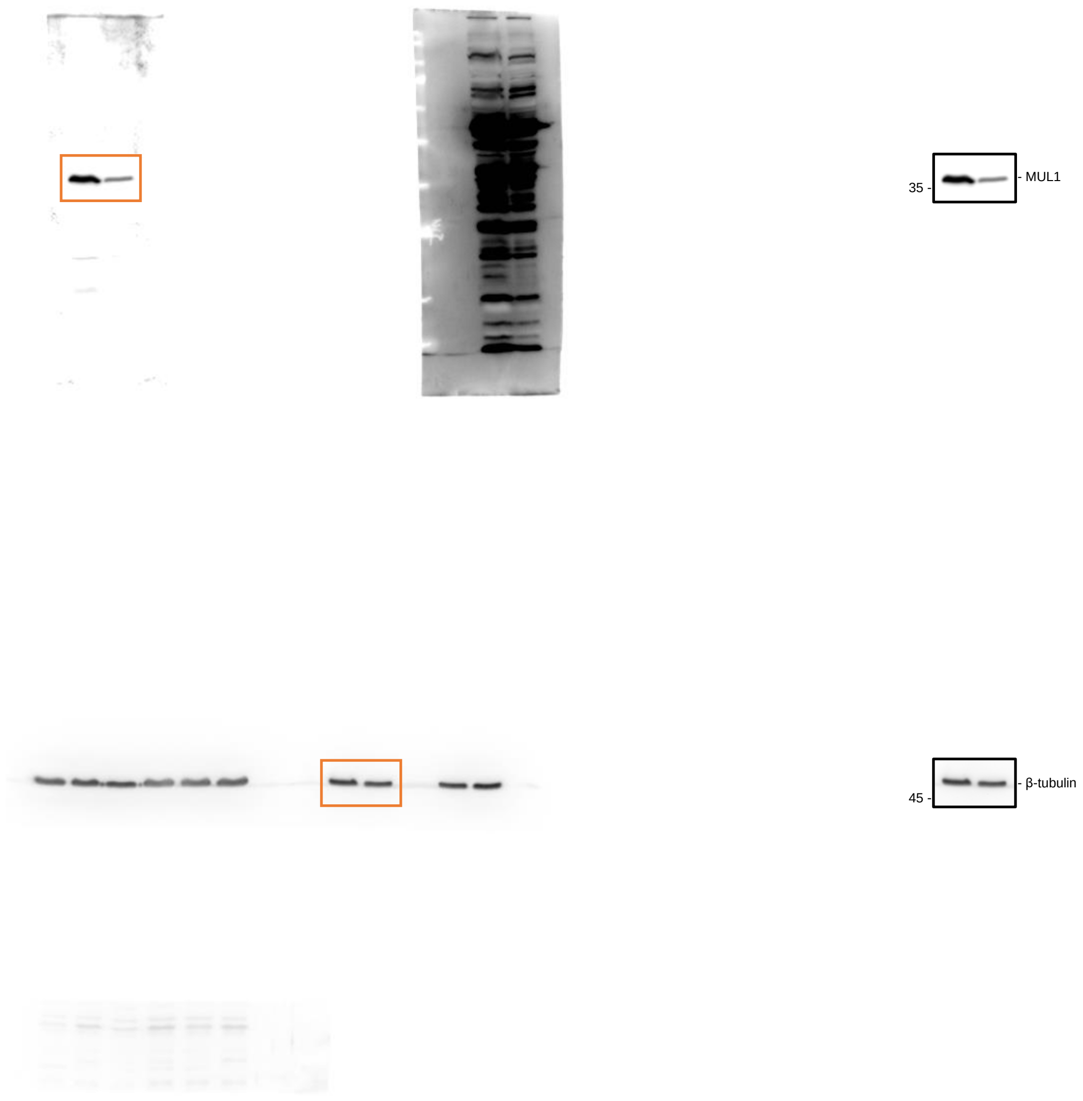

Unprocessed Western blots of Figure S5b (contiune)

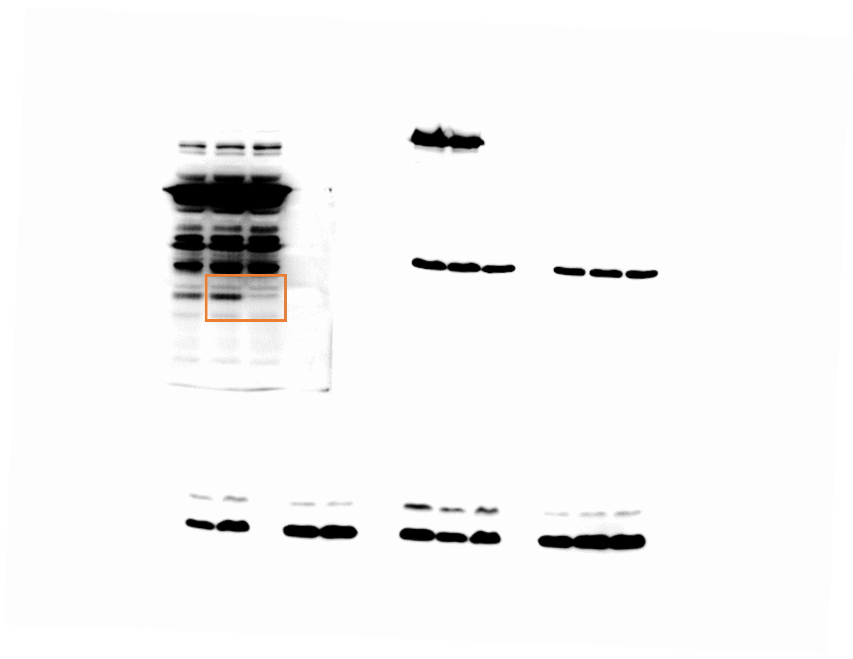

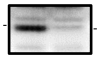

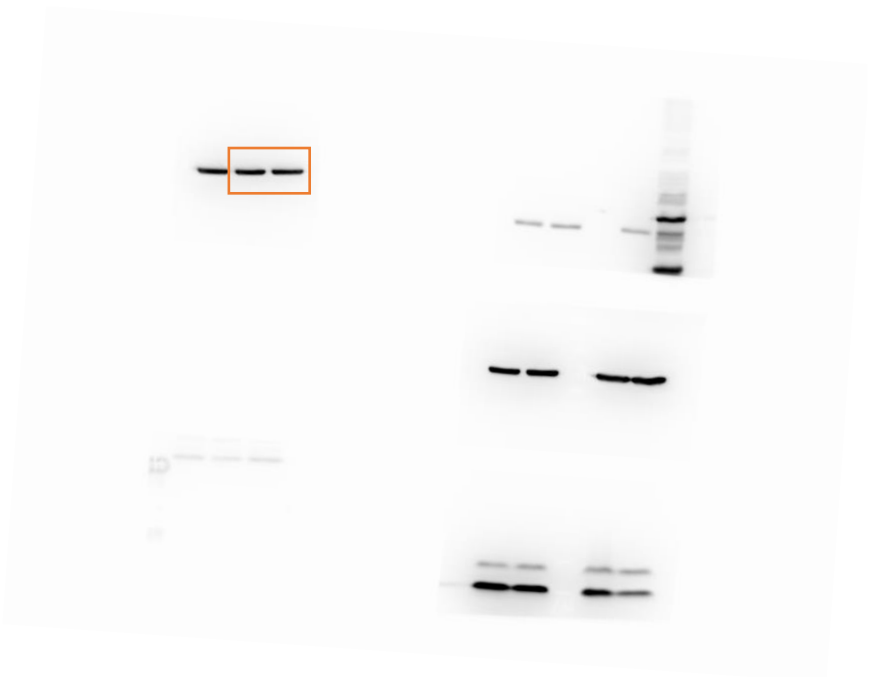

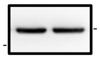

Unprocessed Western blots of Figure S5c

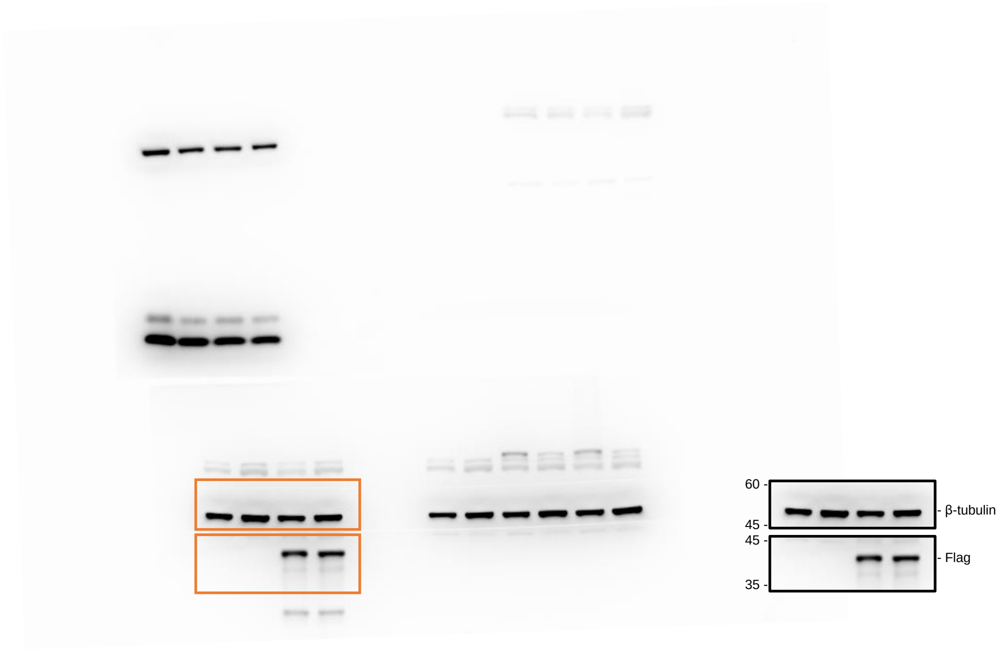

Unprocessed Western blots of Figure S5d

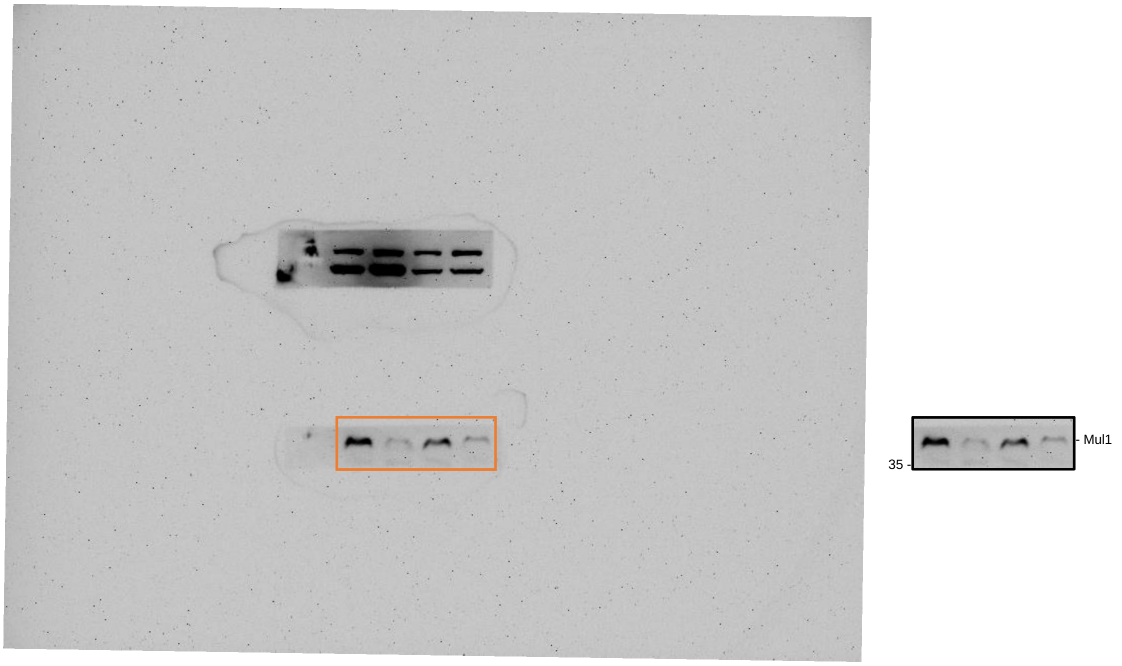

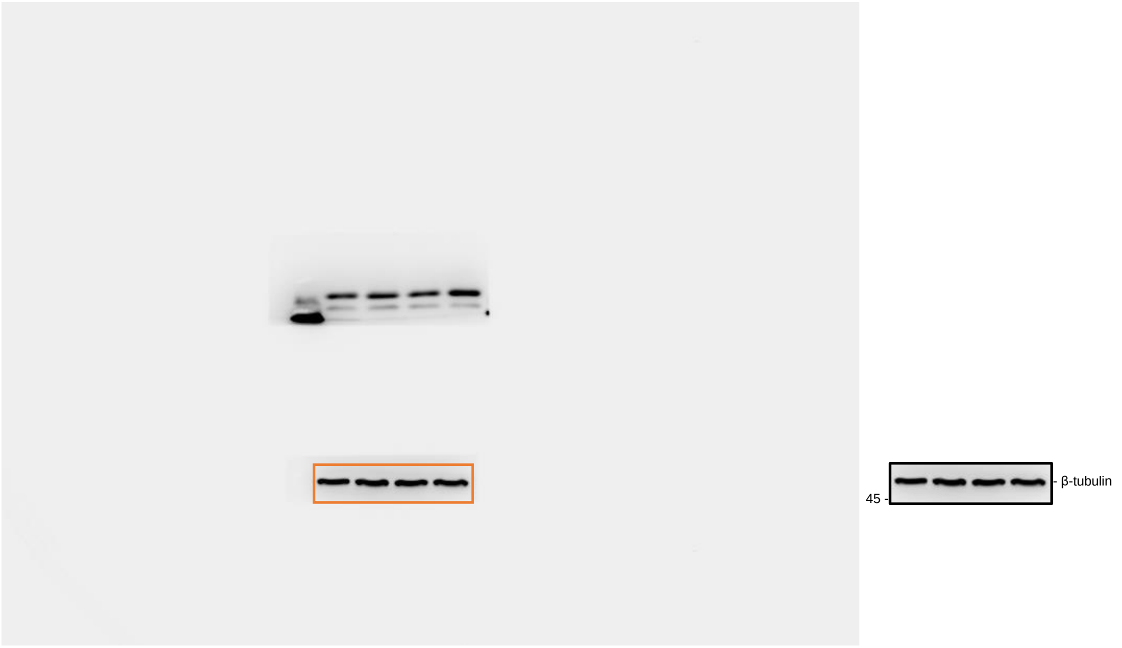

Unprocessed Western blots of Figure S5e

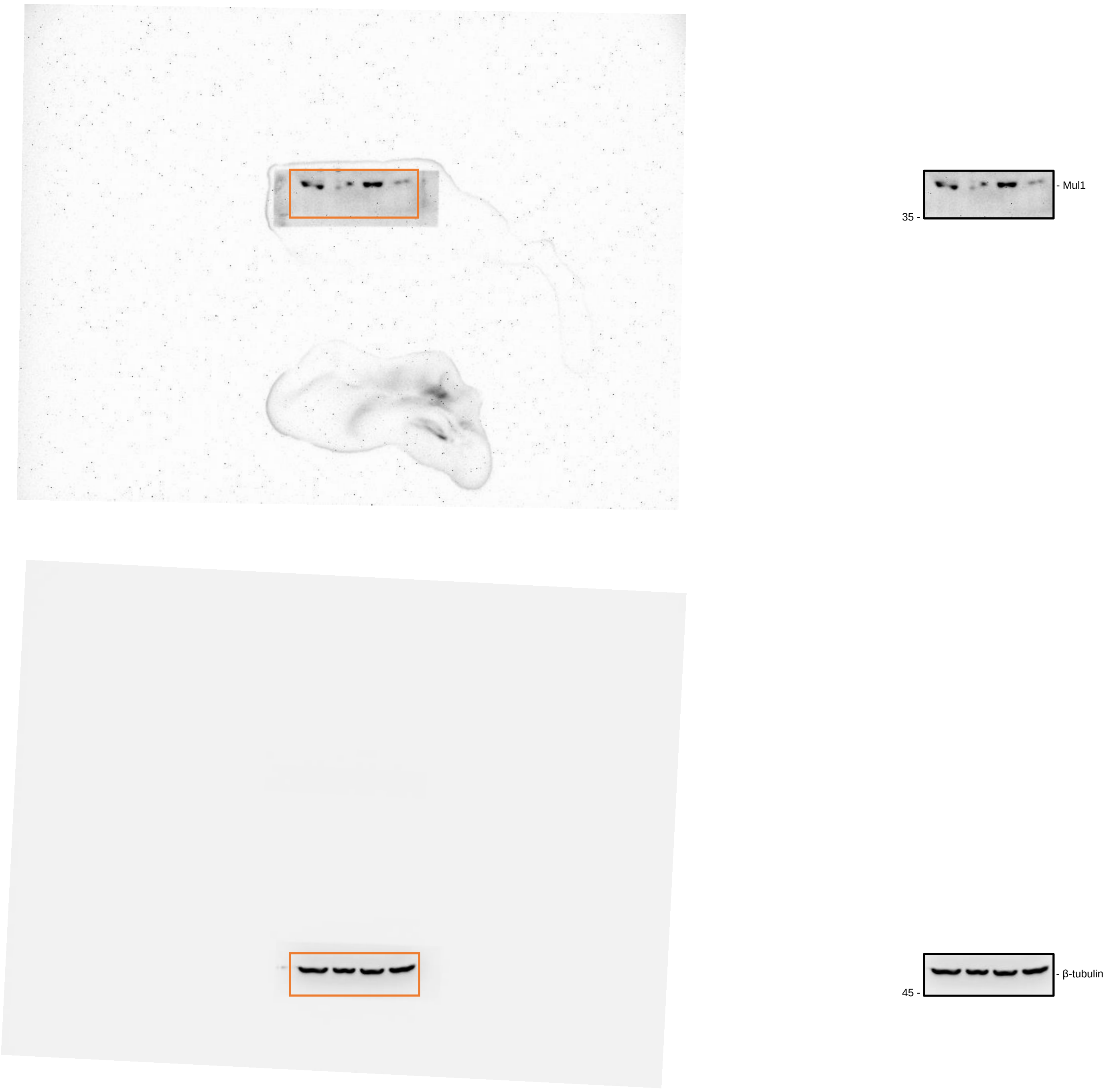

Unprocessed western blots of Figure S5f

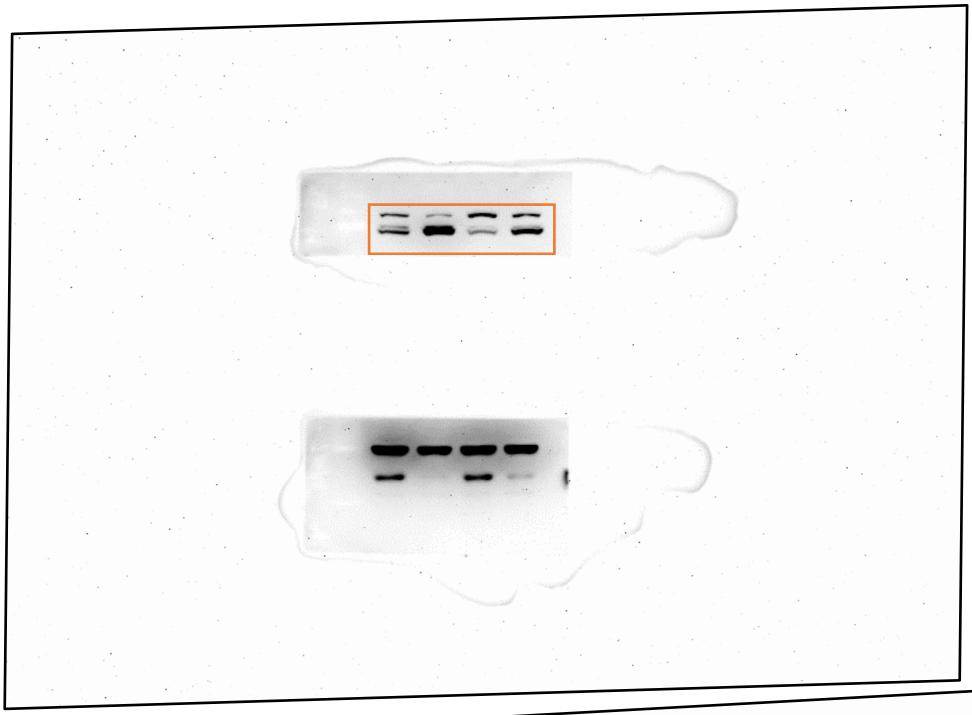

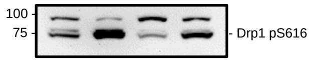

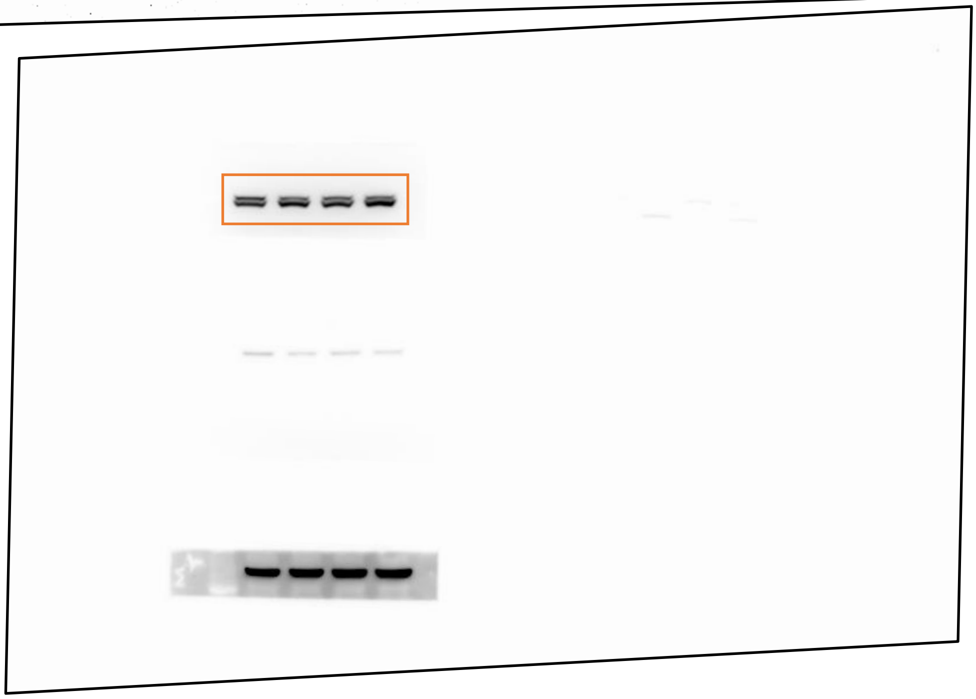

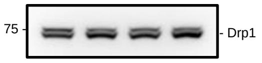

Unprocessed western blots of Figure S5f (continue)

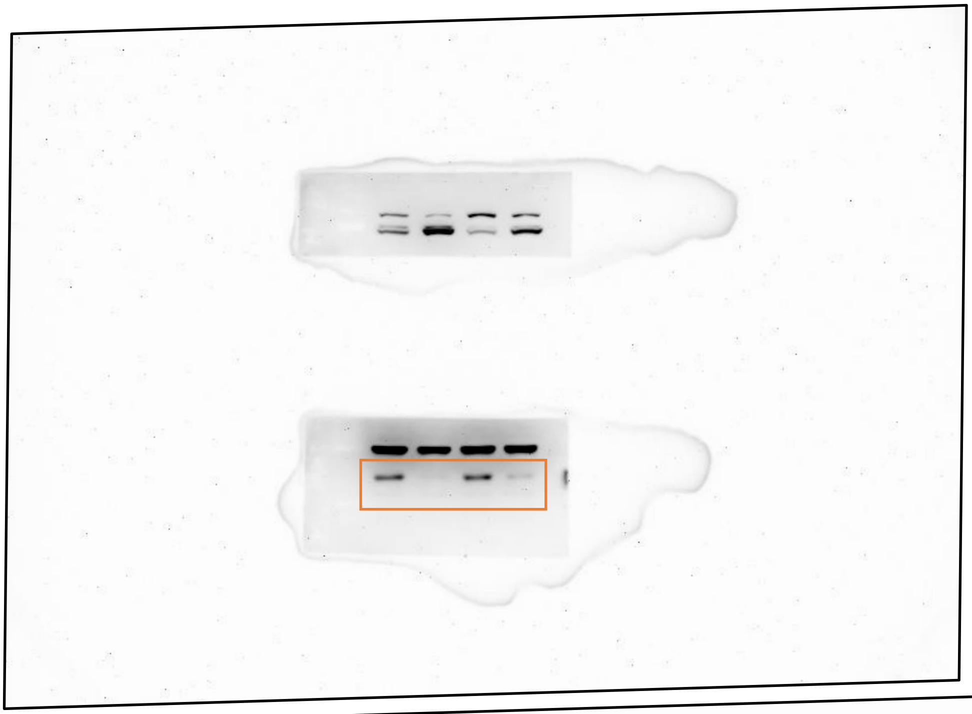

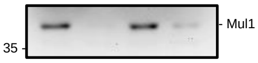

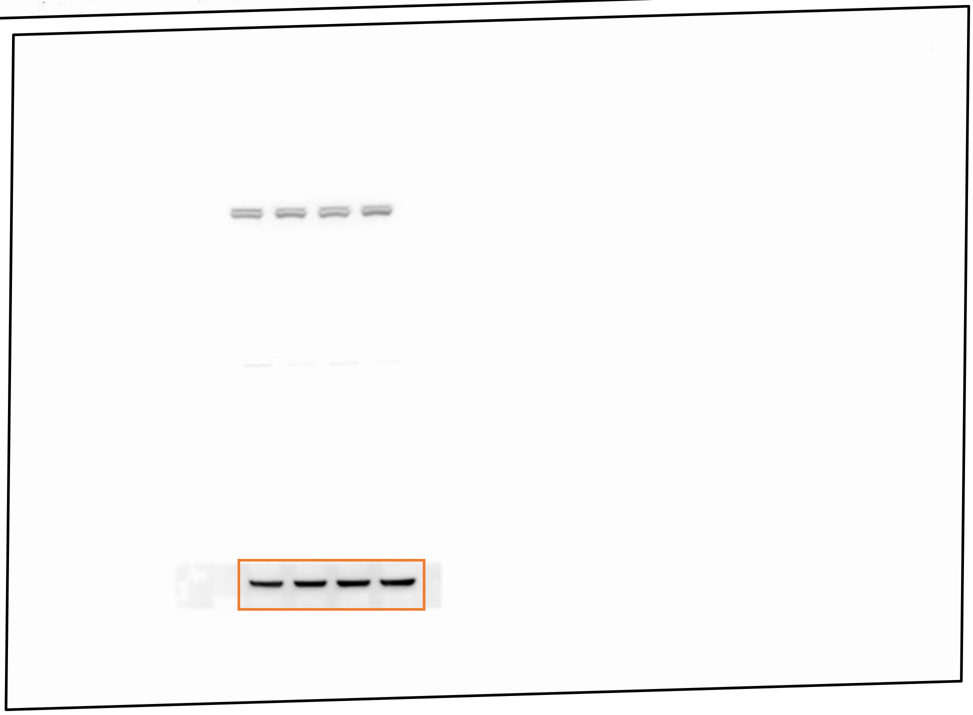

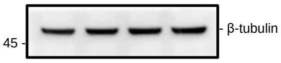

Unprocessed Western blots of Figure S6b

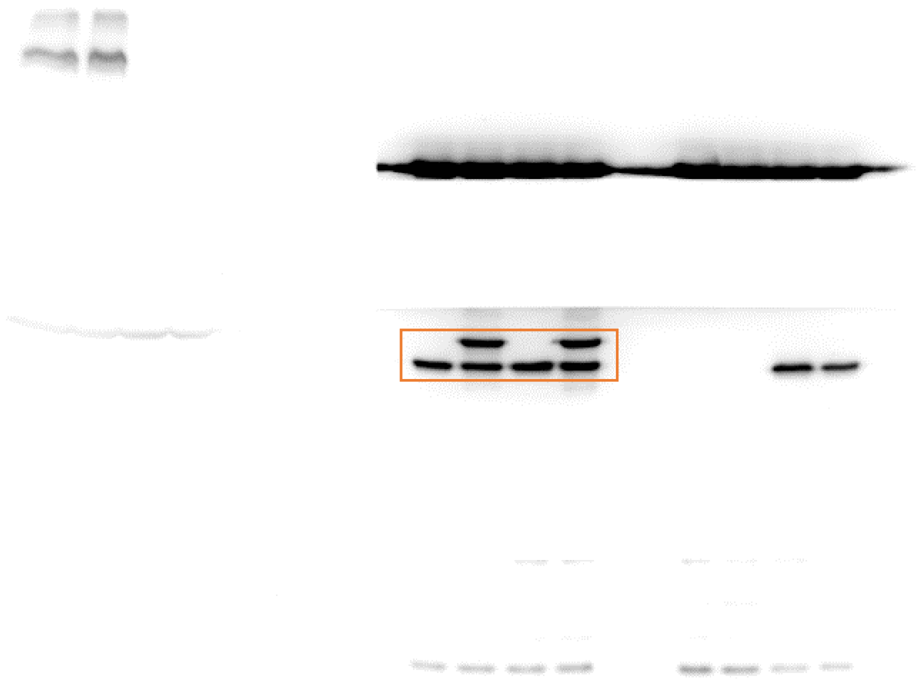

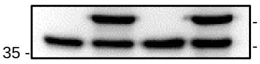

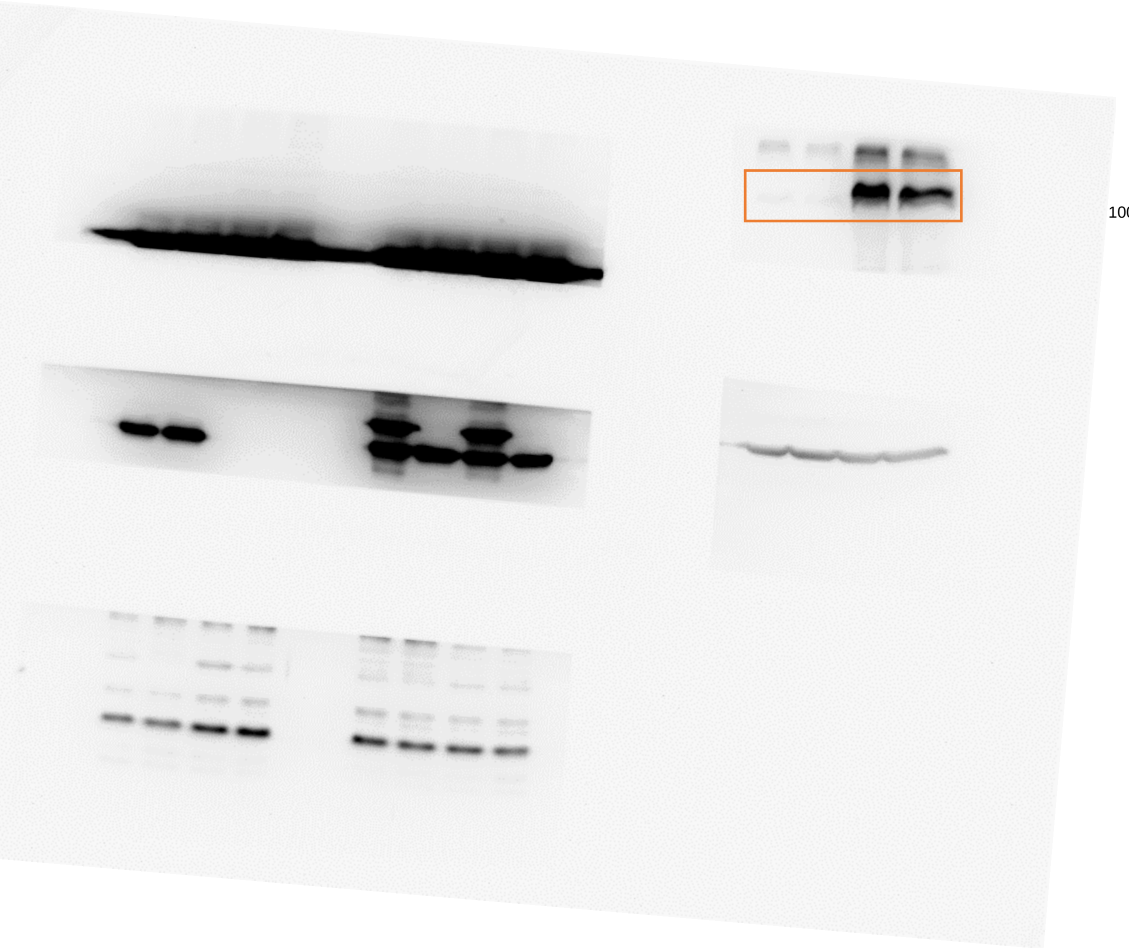

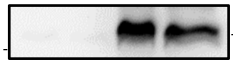

Unprocessed Western blots of Figure S6b (continue)

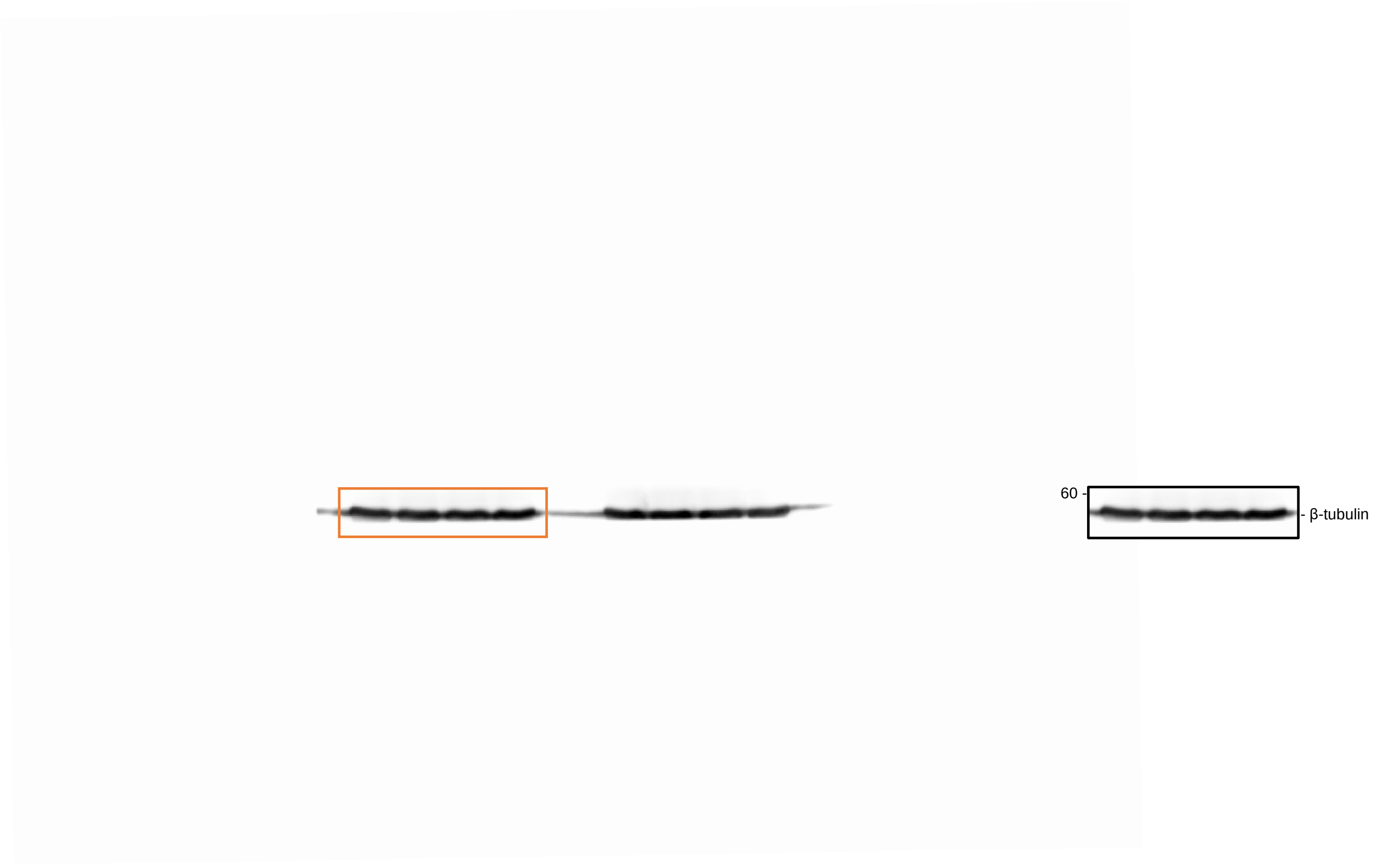

Unprocessed Western blots of Figure S6d

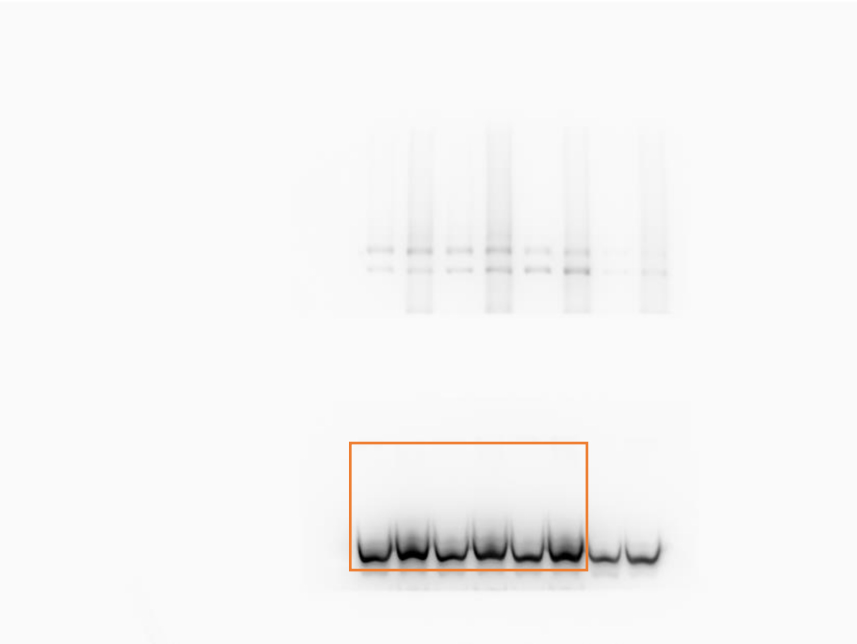

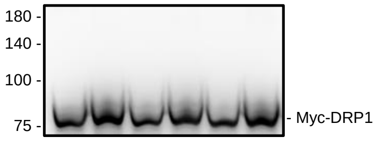

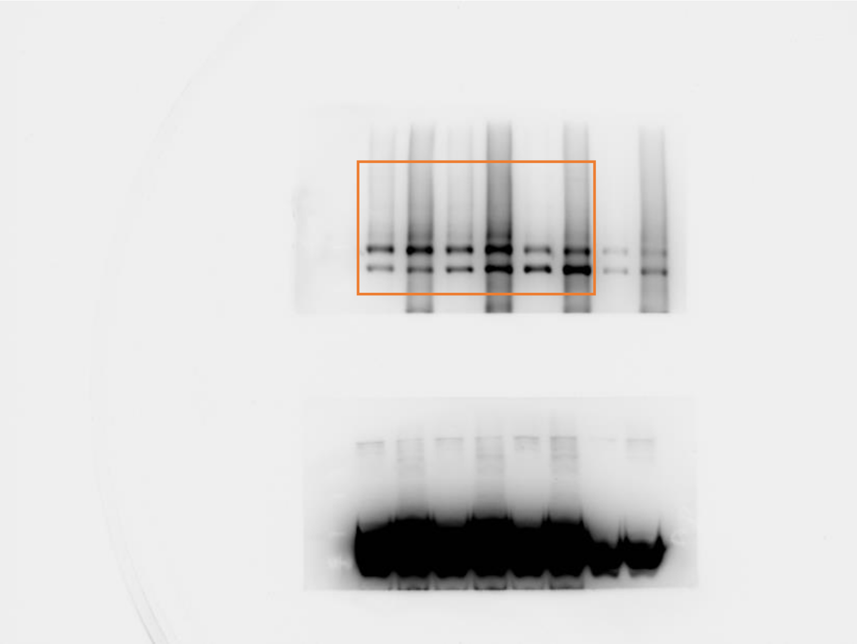

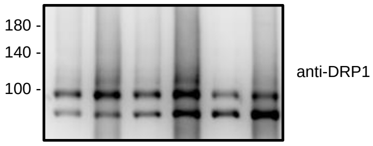

Unprocessed Western blots of Figure S6d (continue)

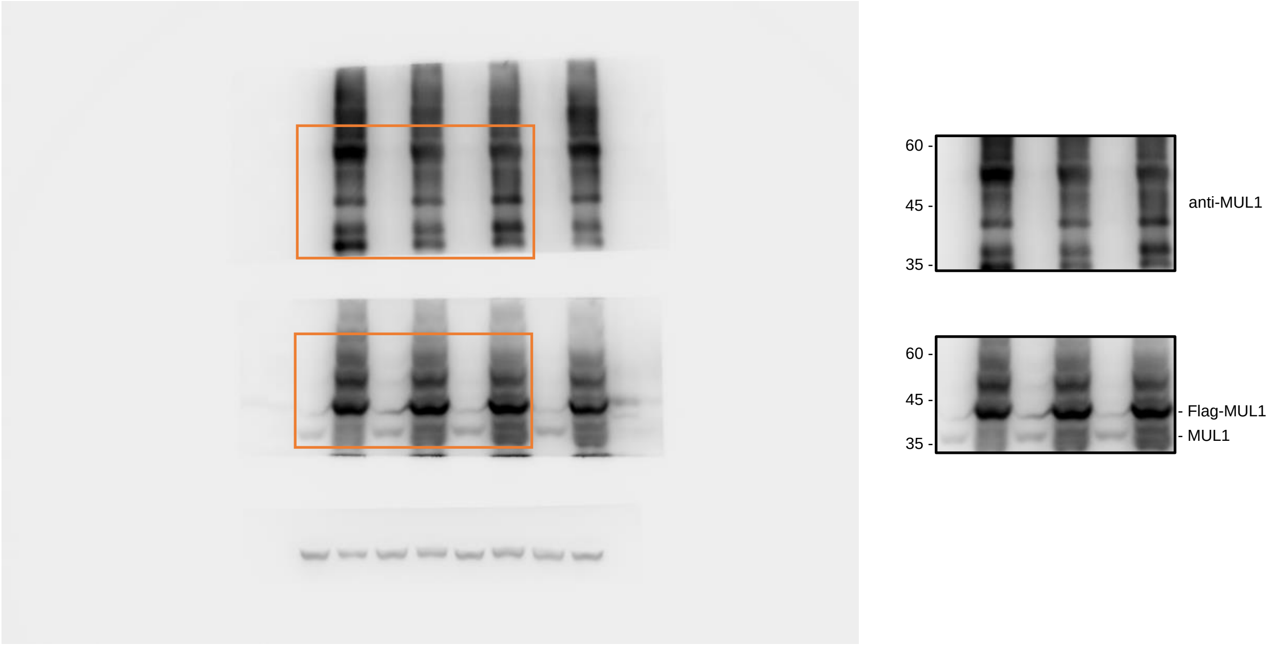

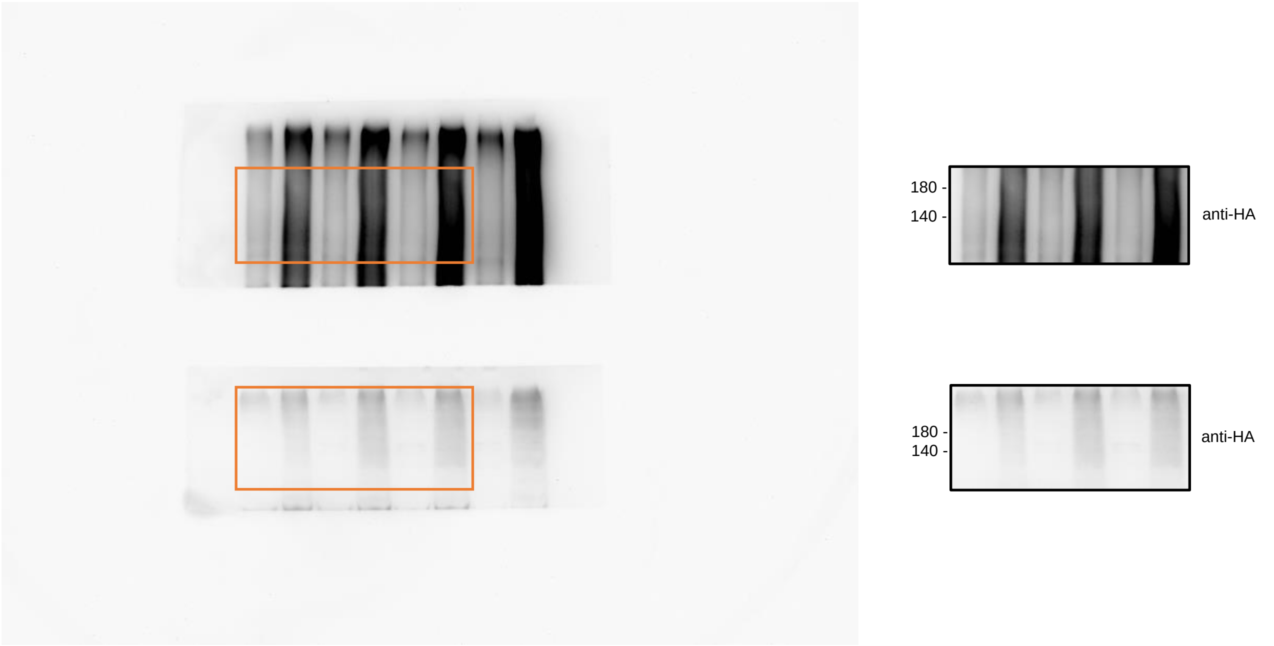

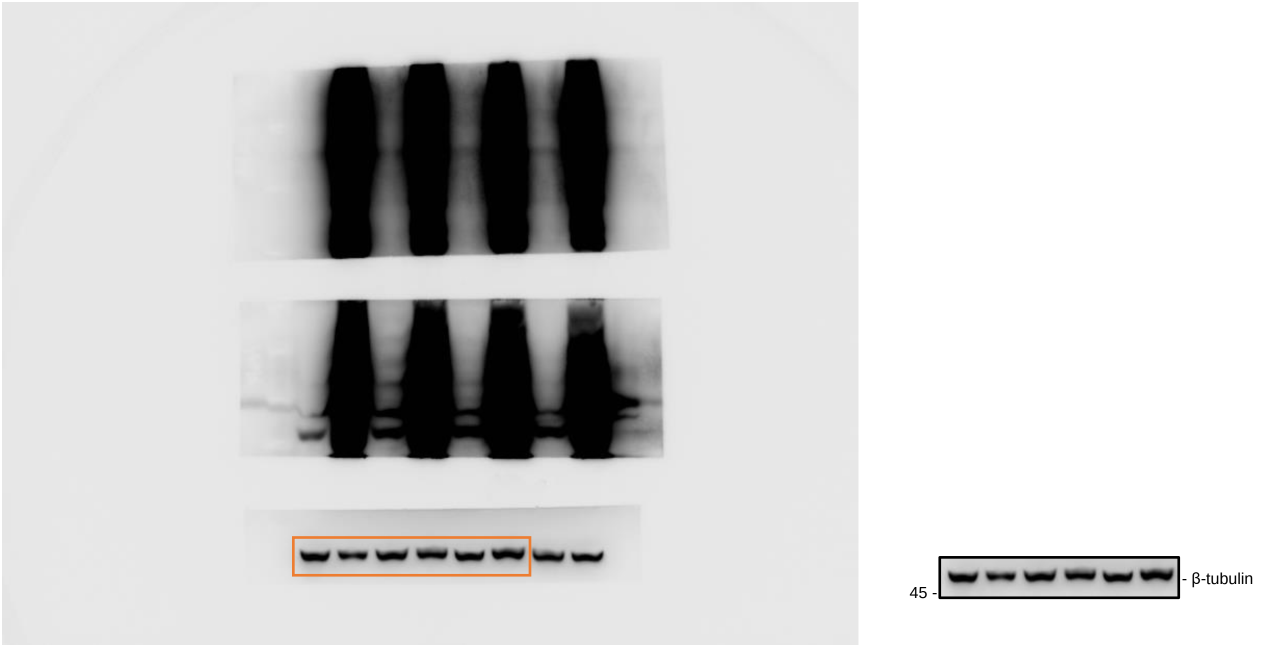

Unprocessed Western blots of Figure S6d (continue)

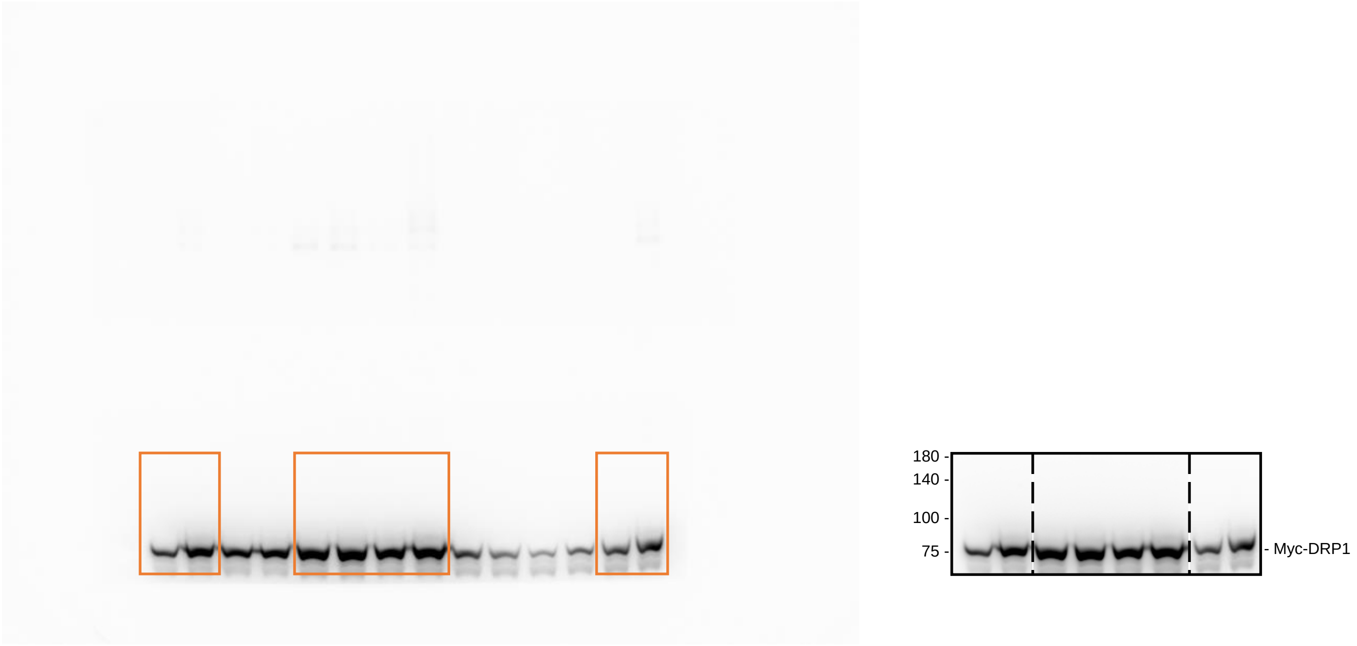

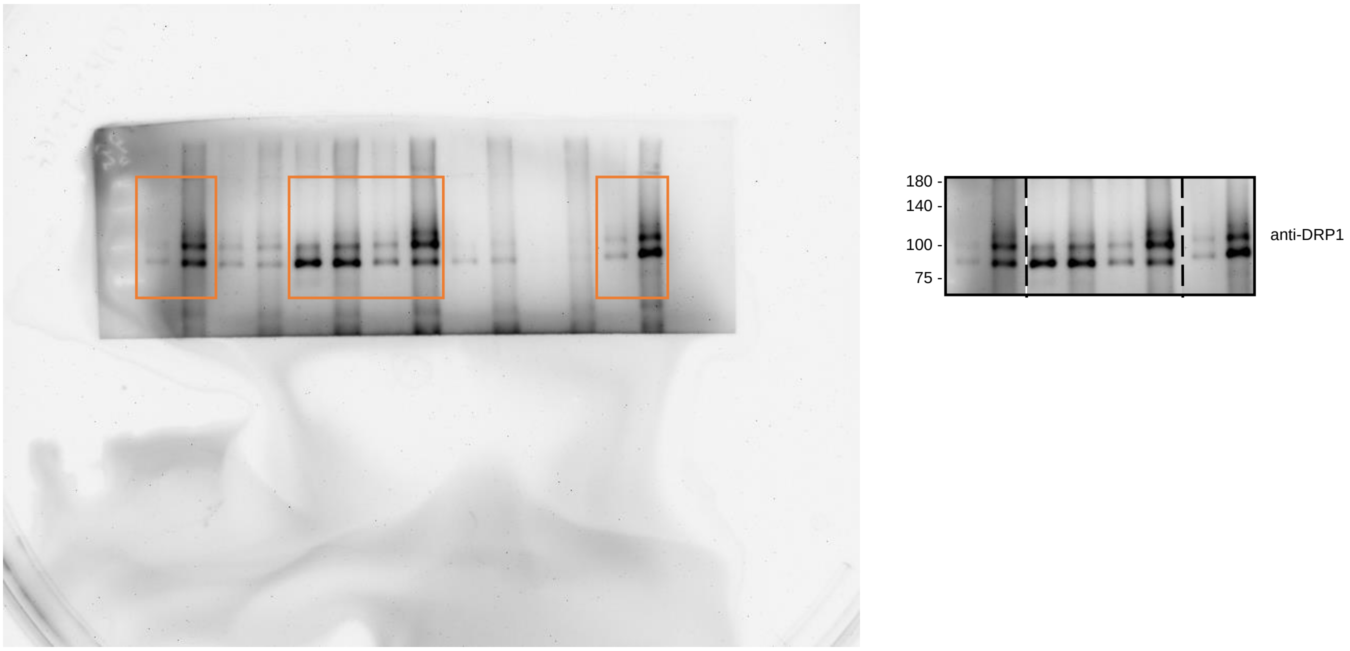

Unprocessed Western blots of Figure S6d (continue)

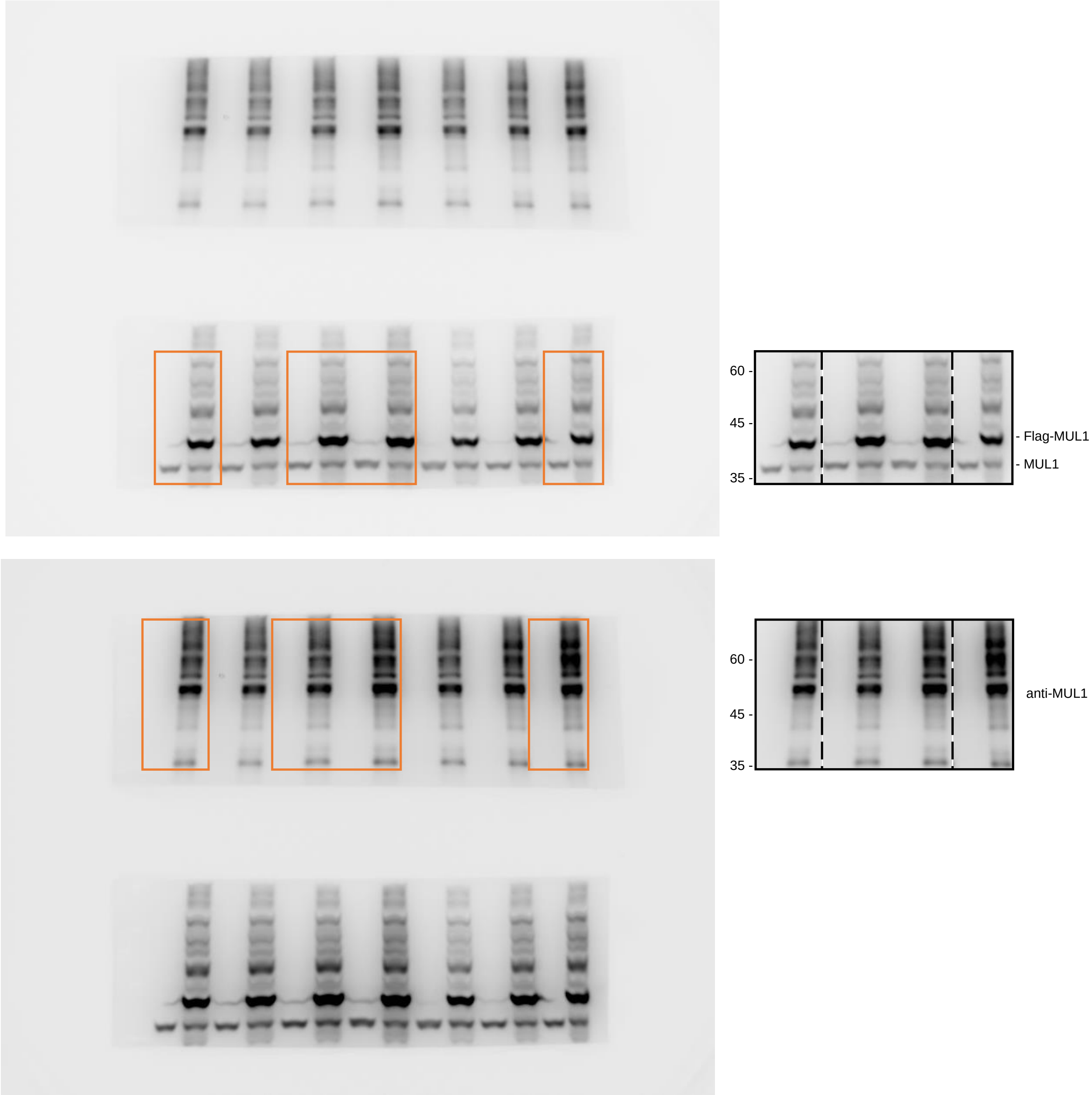

Unprocessed Western blots of Figure S6d (continue)

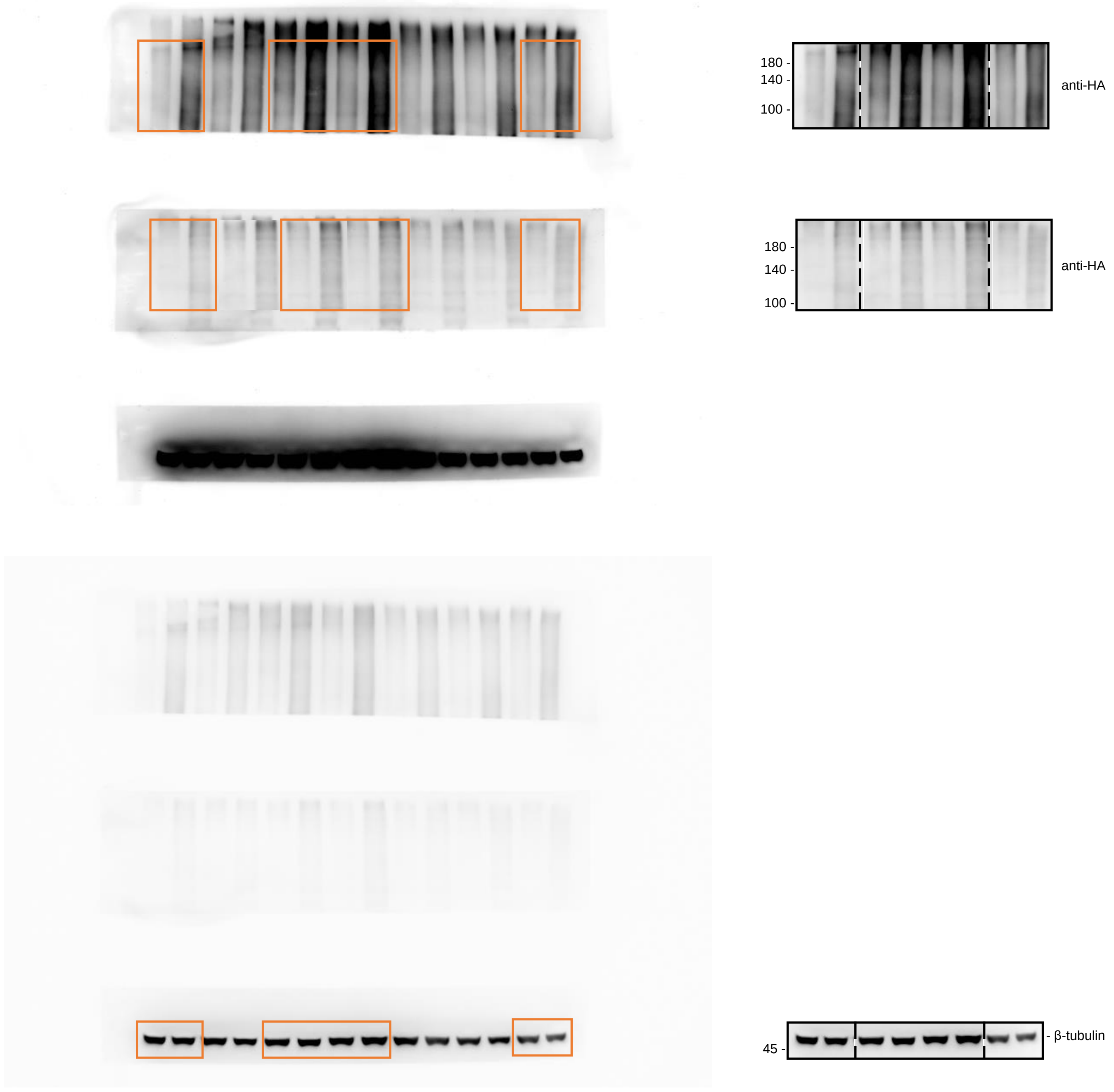

Unprocessed Western blots of Figure S6d (continue)

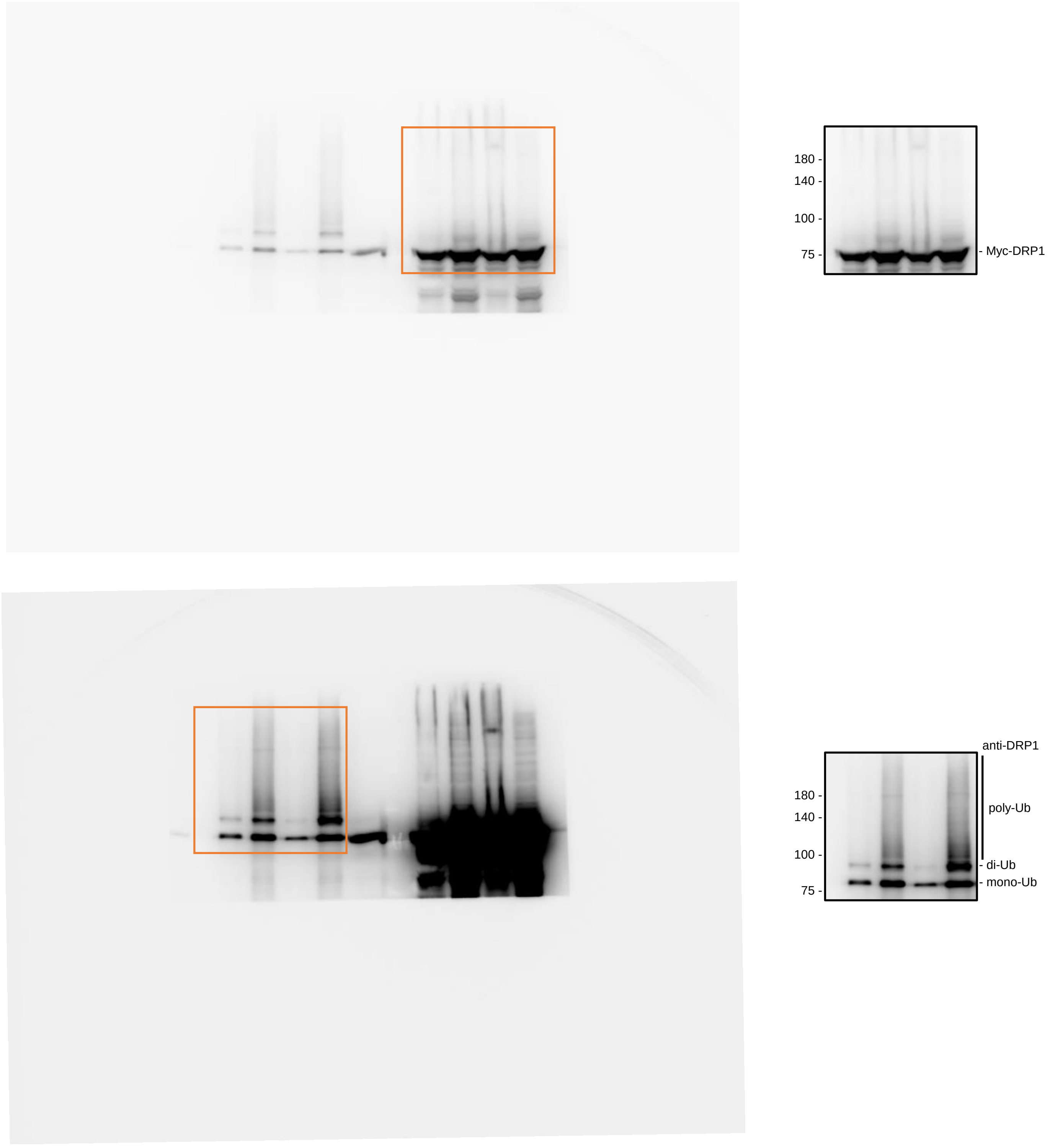

Unprocessed Western blots of Figure S6d (continue)

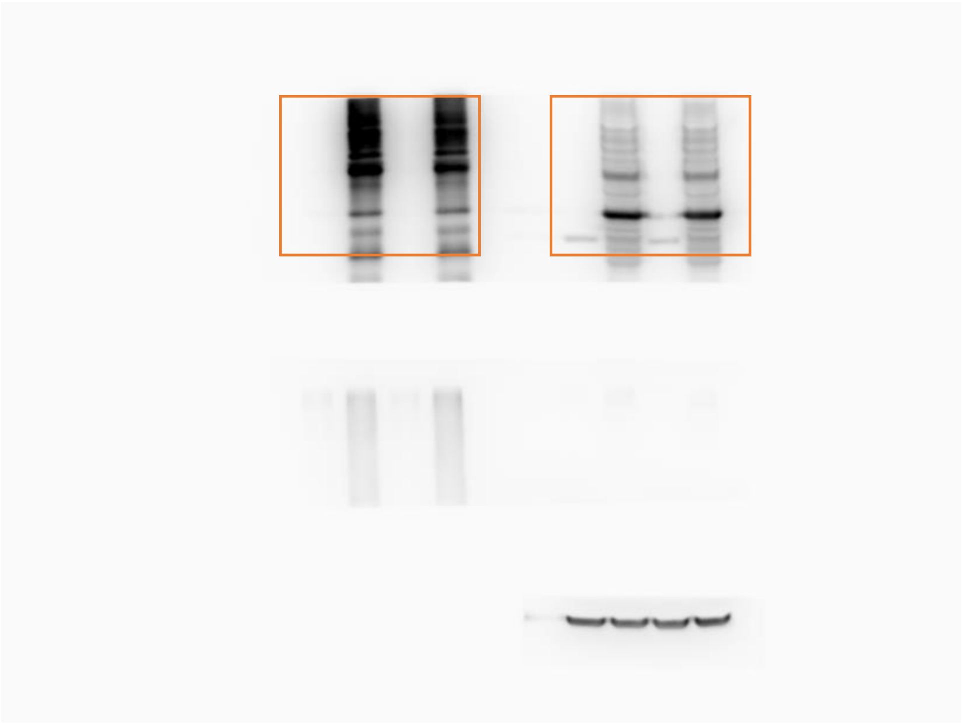

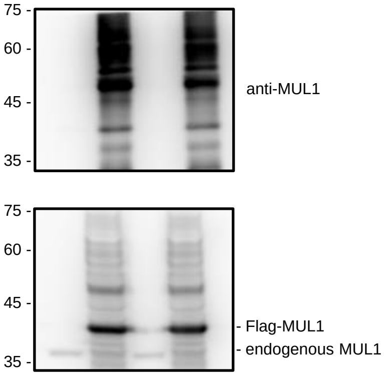

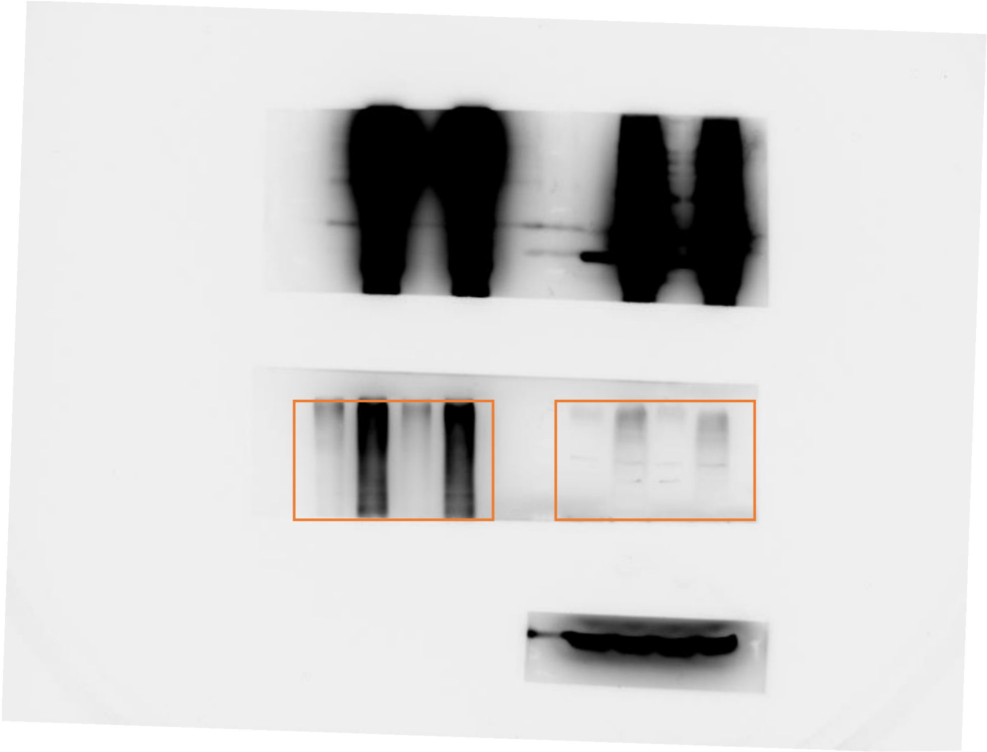

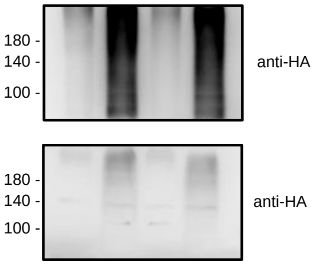

Unprocessed Western blots of Figure S6d (continue)

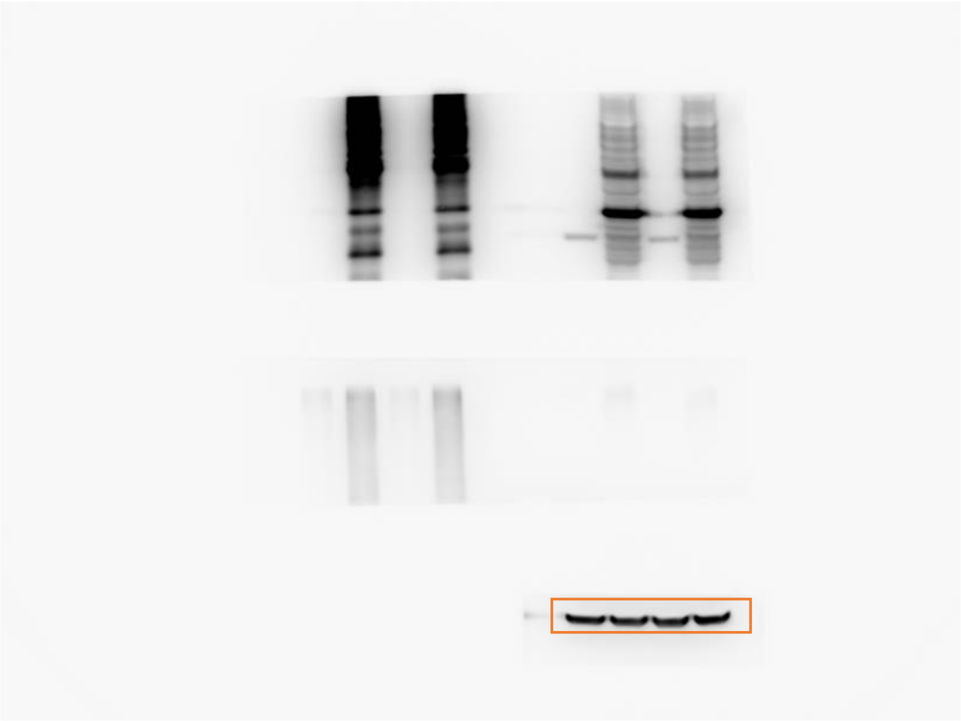

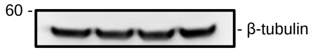

Unprocessed Western blots of Figure S6e

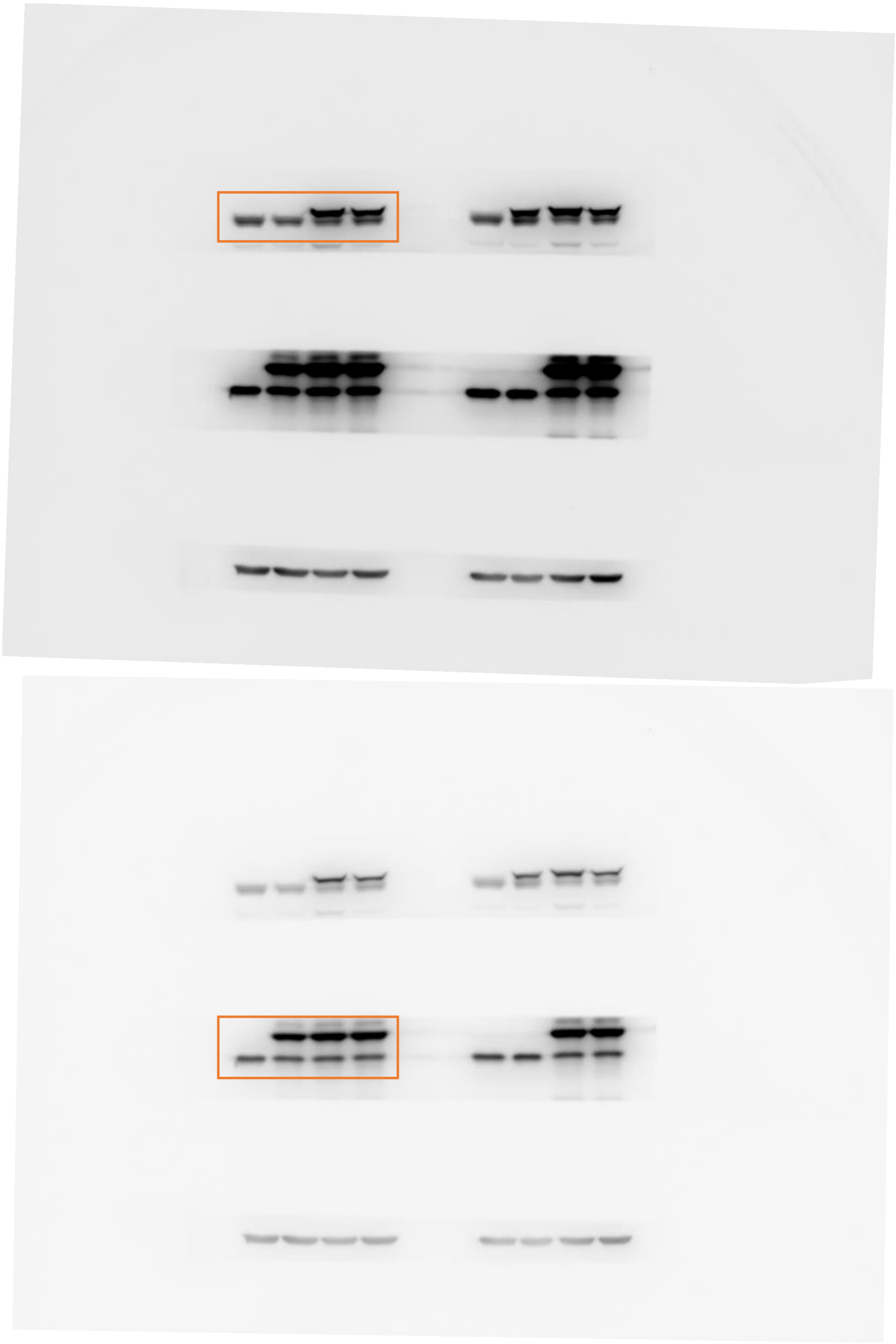

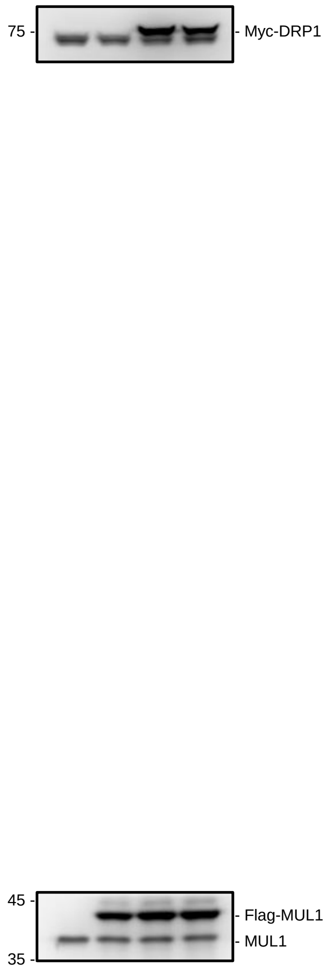

Unprocessed Western blots of Figure S6e (continue)

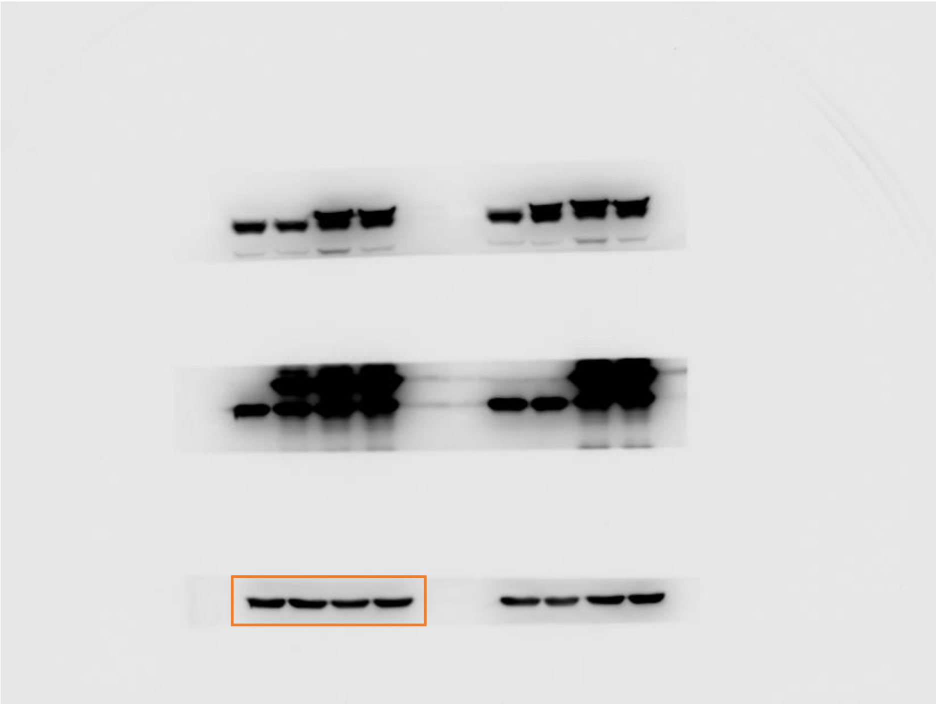

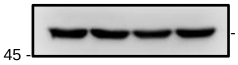

Supplement: Supplementary file 7 — Source Data [file 41467_2024_46385_MOESM7_ESM.zip › Original Western blots.pdf]
